# Unravelling the mechanisms of antibiotic and heavy metal resistance co-selection in environmental bacteria

**DOI:** 10.1093/femsre/fuae017

**Published:** 2024-06-19

**Authors:** Brodie F Gillieatt, Nicholas V Coleman

**Affiliations:** School of Life and Environmental Sciences, The University of Sydney, F22 - LEES Building, NSW 2006, Australia; School of Natural Sciences, and ARC Centre of Excellence in Synthetic Biology, Macquarie University, 6 Wally’s Walk, Macquarie Park, NSW 2109, Australia

**Keywords:** metals, antibiotics, resistance, selection, evolution, plasmids

## Abstract

The co-selective pressure of heavy metals is a contributor to the dissemination and persistence of antibiotic resistance genes in environmental reservoirs. The overlapping range of antibiotic and metal contamination and similarities in their resistance mechanisms point to an intertwined evolutionary history. Metal resistance genes are known to be genetically linked to antibiotic resistance genes, with plasmids, transposons, and integrons involved in the assembly and horizontal transfer of the resistance elements. Models of co-selection between metals and antibiotics have been proposed, however, the molecular aspects of these phenomena are in many cases not defined or quantified and the importance of specific metals, environments, bacterial taxa, mobile genetic elements, and other abiotic or biotic conditions are not clear. Co-resistance is often suggested as a dominant mechanism, but interpretations are beset with correlational bias. Proof of principle examples of cross-resistance and co-regulation has been described but more in-depth characterizations are needed, using methodologies that confirm the functional expression of resistance genes and that connect genes with specific bacterial hosts. Here, we comprehensively evaluate the recent evidence for different models of co-selection from pure culture and metagenomic studies in environmental contexts and we highlight outstanding questions.

## Teaser

Co-selection between metal and antibiotics results in the persistence of resistance genes, but more work is needed to understand the relative roles of co-resistance, cross-resistance and co-regulation mechanisms in causing co-selection in the environment.

## Introduction

The proliferation of antibiotic resistance genes (ARGs) amongst bacterial pathogens underpins the problem of antimicrobial resistance. This is becoming a leading cause of mortality, associated with 4.95 million deaths globally in 2019 and projected to become 10 million deaths annually in 2050 (Murray et al. 2022). The widespread use and misuse of antibiotics are known to cause a large part of the antimicrobial resistance problem, but beyond the clinical setting, an interplay between resistance genes for antibiotics, other selective agents like metals, and mobile resistance elements (MGEs) is also occurring. This warrants a ‘One Health’ approach to this problem (Durso and Cook 2019). The topic of antibiotic resistance is frequently reviewed, and the reader is directed to recent reviews that focus on the more environmental aspects (Skandalis et al. 2021, Larsson and Flach 2022). What is less well-understood, and the subject of this review, is how other selective agents, namely heavy metals, impact the acquisition and transmission of ARGs.

In the broadest sense, co-selection is where exposure to one selective agent allows adaptation to a second selective agent, in this context where one antimicrobial selects for a resistance mechanism for both itself and also another antimicrobial (Di Cesare et al. 2016a). There are three recognized genetic models of co-selection: co-resistance, cross-resistance, and co-regulation (Baker-Austin et al. 2006); these are detailed in subsequent sections. A fourth model, co-selection via biofilm formation, is not a genetic model and does not form part of this review.

There are several potential co-selective agents for antibiotic resistance including detergents (Grenni and Corno 2019), biocides (Wales and Davies 2015), polyaromatic hydrocarbons, polychlorinated biphenyls (Gorovtsov et al. 2018), and nanoparticles (Chen et al. 2019b, Zhang et al. 2019b). Here, we will focus just on the role of metals in the co-selection of antimicrobial resistance since metals have the largest sphere of influence due to their diverse anthropogenic and natural sources, and their persistence in environmental niches. Individual resistance genes are common in environmental bacteria irrespective of anthropogenic pollutant exposure (Lo Giudice et al. 2013, Farias et al. 2015) and certainly predate anthropogenic pollution due to natural exposure to antibiotics and metals (Mindlin et al. 2005, Petrova et al. 2011). However, the increased dissemination and abundance of these resistance genes over human history correlates with the anthropogenic use of mercury (Poulain et al. 2015), copper (Staehlin et al. 2016), and antibiotics (Knapp et al. 2010). Moreover, parallel responses in the increased persistence and diversity of resistance genes, and in the assembly of multiple resistance genes into single MGEs, have been observed in recent decades (Hughes and Datta 1983, van Hoek et al. 2011).

This review comprehensively evaluates an outstanding gap in knowledge, i.e. what is the evidence supporting different mechanisms of heavy metal and antibiotic co-selection? Building on previous seminal reviews (Baker-Austin et al. 2006, Seiler and Berendonk 2012, Pal et al. 2017, Vats et al. 2022), we offer an in-depth picture of co-selective mechanisms. Knowing these mechanisms is important for ameliorating the rise in resistance, as several clinical or environmental solutions to this problem have been proposed based on co-selection theories (Vats et al. 2022). Following an introduction of key concepts including heavy metal contamination, resistance mechanisms, horizontal gene transfer (HGT) and MGEs, and co-selective models and concepts, literature will be discussed that support each of the models. Special focus will be given to research on cadmium, copper, and zinc since these metals are often associated with co-selection. To emphasize modern molecular approaches, the review is focused on recent papers (2010 onwards) (Table S1), with the inclusion of some older studies that made fundamental advances. Common findings including correlational networks across the literature have been summarized to point out potential links that warrant mechanistic investigation. This is concluded with a discussion of what we do know and gaps in knowledge that are yet to be unravelled.

## Heavy metals, a bacterial perspective

### Heavy metal sources

Heavy metals are defined as naturally occurring metals of an atomic number greater than 20 and density greater than 5 g cm^−3^ (Ali and Khan 2018). As this review is concerned with eco-toxicity, this definition will be extended to include toxic metalloids such as arsenic and tellurium and the nonmetal selenium.

Heavy metals can enter the environment from natural sources, such as geothermal activity, fires, weathering, and erosion, but these are minor contributors compared to anthropogenic sources, such as industry, agriculture, and healthcare (Swaine 1994, Gillings and Paulsen 2014). Mineral-based industries, including mining, metal refining, and fossil fuel combustion, are obvious anthropogenic sources of heavy metal contamination. The waterways, sediments, and soils near these industries often have high levels of arsenic, cadmium, chromium, copper, lead, manganese, mercury, nickel, and zinc (Rowe et al. 2002, Thomas et al. 2020, Yang et al. 2021). In agriculture, zinc and copper are common livestock feed supplements for infection control and growth promotion (Yu et al. 2017, Yue et al. 2020). Furthermore, heavy metals are common components of fertilizers, pesticides, and fungicides (Yu et al. 2017, Grenni and Corno 2019). In healthcare, heavy metal use has largely been supplanted by antibiotics, however, it is still retained for some topical treatments and antimicrobial coatings (Pal et al. 2017). The waste streams from these sectors are increasing, and are the major source of metals in waterways, sediments, and soils (Seiler and Berendonk 2012, Silva et al. 2021, Palm et al. 2022).

Metal and antibiotic waste streams often overlap, posing significant co-contamination and microbial toxicity in waterways, sediments, and soils. Later sections will discuss this overlap also with respect to resistance genes. Akin to heavy metals, the manufacture of pharmaceutical antibiotics have introduced a source of antibiotic emissions of unprecedented concentration and scale (Larsson and Flach 2022). In China, which is the largest antibiotic producer and consumer globally, the antibiotics most in use by weight are macrolides, β-lactams, fluoroquinolones, tetracyclines, and sulfonamides (Zhou et al. 2017, Zhao et al. 2018). An estimated 53 800 tonnes of antibiotics were released into the environment after human or animal use in China alone in 2013 (Zhang et al. 2015). It is well known that the discharge of active antibiotics in waste streams imposes selective pressures on surrounding ecosystems (Zhang et al. 2015, Larsson and Flach 2022).

### The microbial toxicity of metals

Heavy metals have a dynamic role in biology, and many are essential for cellular function (Nies 1999). Essential metals such as copper, zinc, cobalt, and nickel are toxic at higher concentrations, and others such as cadmium and mercury have no known biological function (Seiler and Berendonk 2012). Metal toxicity triggers the release of reactive oxygen species, which cause DNA mutation and other cellular damage (Yu et al. 2017).

The microbial impact of metal pollution is dependent on numerous factors, including concentration, ion valency, bioavailability, and environmental context. Polyvalent metal ions have different solubilities, reactive potentials and hence toxicities. For example, Cr^6+^ and As^3+^ are more soluble, reactive and toxic than Cr^3+^ and As^5+^ (Nies 1999). The toxic impacts of metals are often ameliorated in solid matrices (sediments and soils) since most metal ions in these niches are bound to anionic residues on the soil surface and are not readily bioavailable (Peltier et al. 2010, Song et al. 2017, Hung et al. 2022). In general, the solubility of free metal ions and organometals is inversely proportional to the pH, which directly impacts uptake into the bacterium, and therefore toxicity (Olaniran et al. 2013). There are exceptions, however, e.g. hydroxo-zinc, cadmium, or nickel groups are soluble at higher pH (Olaniran et al. 2013). Bioavailability is further influenced by the mineral content (Gorovtsov et al. 2018), redox potential, oxygenation, and organic content (Schulz-Zunkel and Krueger 2009). These factors must be considered when determining the toxic or selective effects of metals on microbes.

Heavy metals potentially have a larger sphere of influence than antibiotics. Many heavy metals (arsenic, cadmium, chromium, copper, iron, lead, manganese, nickel, and zinc) are released at concentrations several orders of magnitude higher than antibiotics (Baker-Austin et al. 2006, Yang et al. 2021), they are more widespread, and they do not biodegrade (Grenni and Corno 2019). Furthermore, some metals are known to have lower sorption potential than antibiotics, e.g. zinc and copper compared to tetracycline (Song et al. 2017), thus boosting their bioavailability in soils and sediments (Schulz-Zunkel and Krueger 2009).

## The resistome

The clinical definition of ‘resistance’ refers to the growth of a bacterial strain at higher than the antimicrobial breakpoint value, the breakpoint value being the concentration that determines whether a strain is considered resistant or susceptible. ‘Tolerance’ is clinically defined as an increase in minimum bactericidal concentration without an increase in minimum inhibitory concentration (MIC) (Wales and Davies 2015). These terms cannot be used in their strict clinical sense for most heavy metals, as breakpoint values and MICs may not exist. So it is important to note that when we use the word ‘resistance’ here, this has a more general meaning, i.e. being able to grow at a heavy metal concentration that would normally inhibit growth (Wales and Davies 2015).

Antibiotics and heavy metals exert a powerful selective pressure on microorganisms, which has led to the evolution of various resistance and homeostatic mechanisms (Staehlin et al. 2016, Squadrone 2020). The collection of resistance genes in a given environment is known as the ‘resistome’. The antibiotic and heavy metal resistomes are diverse and ARGs and metal resistance genes (MRGs) are common in most environments, with a range of 10^−5^–10^−1^ ARGs per copy of 16S rDNA in bacterial genomes and metagenomes (Pal et al. 2016, Zhao et al. 2018, Thomas et al. 2020), and 86% of complete genomes contain potential MRGs (Pal et al. 2015). While ARGs generally are *bona fide* resistance genes, many MRGs may also play roles in maintaining the homeostasis of essential metals, only providing resistance at higher metal concentrations. It is the latter capacity that we will focus on here. The more predominant MRGs and ARGs are shown in Tables 1 and 2, respectively.

**Table 1.** Examples of heavy metal resistance mechanisms, genes, targets, and bacterial hosts taxa. Gene names are synonymous with protein product.

Mechanism	Gene/protein	Target	Taxa	References
Chemical modification	*cue*	Cu	*Pseudomonadota*	Rensing and Grass (2003)
	*mer*	Hg	*Actinomycetota, Bacillota, Pseudomonadota*	Barkay et al. (2003)
Chemical modification, efflux	*ars*	As, Sb	*Actinomycetota, Bacillota*, CFB Group, *Pseudomonadota*	Ben Fekih et al. (2018)
	*cop*	Cu	*Bacillota, Pseudomonadota*	Monchy et al. (2006)
	*pco*	Cu	*γ-Proteobacteria*	Staehlin et al. (2016)
Efflux	*chr*	Cr	*Actinomycetota, Bacillota*, CFB Group, *Pseudomonadota*	Branco et al. (2008)
	*cnr*	Co, Ni	*Pseudomonadota, Planctomycetes*	Marrero et al. (2007)
	*cus*	Ag, Cu	*Pseudomonadota*	Staehlin et al. (2016)
	*czc*	Cd, Co, Zn	CFB Group, *Pseudomonadota*	Nies (1995)
	*czr*	Cd, Zn	*Actinomycetota, Bacillota, Pseudomonadota*	Hassan et al. (1999)
	*ncc*	Ni, Co, Cd	*Pseudomonadota*	Schmidt and Schlegel (1994)
	*sil*	Ag	*Pseudomonadota*	Gupta et al. (1999)
	*tcr*	Cu	*Bacillota*	Hasman and Aarestrup (2002)
	*znt*	Zn	*Pseudomonadota*	Takahashi et al. (2015)
Efflux, sequestration	*pbr*	Pb	*Bacillota, Pseudomonadota*	Borremans et al. (2001)

**Table 2.** Examples of antibiotics resistance mechanisms, genes, targets, and bacterial host taxa. Protein name only indicates when it differs from the gene name.

Mechanism	Gene/protein	Target	Taxa	References
Chemical modification	*aac*	Aminoglycosides	*Actinomycetota, Bacillota, Pseudomonadota*	Shaw et al. (1993)
	*ant*	Aminoglycosides	*Actinomycetota, Bacillota*, CFB Group, *Pseudomonadota*	Shaw et al. (1993)
	*aph*	Aminoglycosides	*Actinomycetota, Bacillota, Pseudomonadota*	Shaw et al. (1993)
	*bla*	β-lactams	*Actinomycetota, Bacillota, Pseudomonadota*	King et al. (2016)
	*cat*	Phenicols	*Actinomycetota, Bacillota, Pseudomonadota*	Schwarz and Cardoso (1991)
	*ere*	Macrolides	*Actinomycetota, Bacillota*, CFB Group, *Pseudomonadota*	Morar et al. (2012)
	*mph*	Macrolides	*Bacillota, Pseudomonadota*	Pawlowski et al. (2018)
	*vat*	Streptogramins	*Bacillota, Pseudomonadota*	Allignet et al. (1993)
Efflux	*acr*	Aminoglycosides, β-lactams, fluoroquinolones, macrolides, sulfonamides	*Pseudomonadota*	Chowdhury et al. (2019)
	*cml*	Phenicols	*Actinomycetota, Bacillota, Pseudomonadota*	Bissonnette et al. (1991)
	*flo*	Phenicols	*Pseudomonadota*	Poole (2005)
	*lmr*	Aminoglycosides, β-lactams, fluoroquinolones, lincosamides, tetracyclines	*Actinomycetota, Bacillota, Pseudomonadota*	Poole (2005)
	*mdt*	Multidrug	*Bacillota, Pseudomonadota*	Nishino et al. (2007)
	*mef*	Macrolides	*Bacillota, Pseudomonadota*	Poole (2005)
	*msr*	Macrolides, streptogramin-B	*Bacillota, Pseudomonadota*	Poole (2005)
	*qep*	Fluoroquinolones	*Pseudomonadota*	Strahilevitz et al. (2009)
	*vga*	Lincosamides, streptogramin-A	*Bacillota, Pseudomonadota*	Chesneau et al. (2005)
Chemical modification, efflux	*otr*	Tetracyclines	*Actinomycetota*	van Hoek et al. (2011)
Chemical modification, efflux, target modification	*tet*	Tetracyclines	*Actinomycetota, Bacillota*, CFB Group, *Pseudomonadota*	Poole (2005)
Target modification	*erm*	Macrolides, lincosamides, streptogramin-B	*Actinomycetota, Bacillota*, CFB Group, *Pseudomonadota*	van Hoek et al. (2011)
	*gyr*-	Fluoroquinolones	All	Hooper and Jacoby (2015)
	*par-*	Fluoroquinolones	All	Hooper and Jacoby (2015)
	*qnr*	Fluoroquinolones	*Pseudomonadota*	Strahilevitz et al. (2009)
Target replacement	*dfr*	Trimethoprim	*Actinomycetota, Bacillota*, CFB Group, *Pseudomonadota*	Sköld (2001)
	*mec/*PBP2a	β-lactams	*Bacillota*	Deurenberg and Stobberingh (2008)
	*sul*	Sulfonamides	*Pseudomonadota*	Sköld (2001)
	*van*	Glycopeptides	*Bacillota, Pseudomonadota*	Depardieu et al. (2007)

### Parallels in resistance mechanism: metals versus antibiotics

The mechanisms of metal resistance (Nies 1999) and antibiotic resistance (van Hoek et al. 2011) have been thoroughly reviewed, but it is worth highlighting here the similarities between mechanisms, namely efflux, chemical modification, and sequestration (Baker-Austin et al. 2006). In contrast, some mechanisms that solely apply to antibiotics include modified membrane permeability (Perron et al. 2004), target modification (Timms et al. 1992), or target replacement (Deurenberg and Stobberingh 2008). These mechanisms are not seen for heavy metals as metals present broad toxicity to many cellular systems whereas antibiotics have more specific modes of action and targets in the bacterial cell. Other resistance mechanisms such as general stress responses (e.g. DNA repair and ROS scavengers) do assist in cellular resistance to metals and antibiotics, however, this review will just deal with mechanisms specific to metal ions and antibiotics.

**Figure 1.**
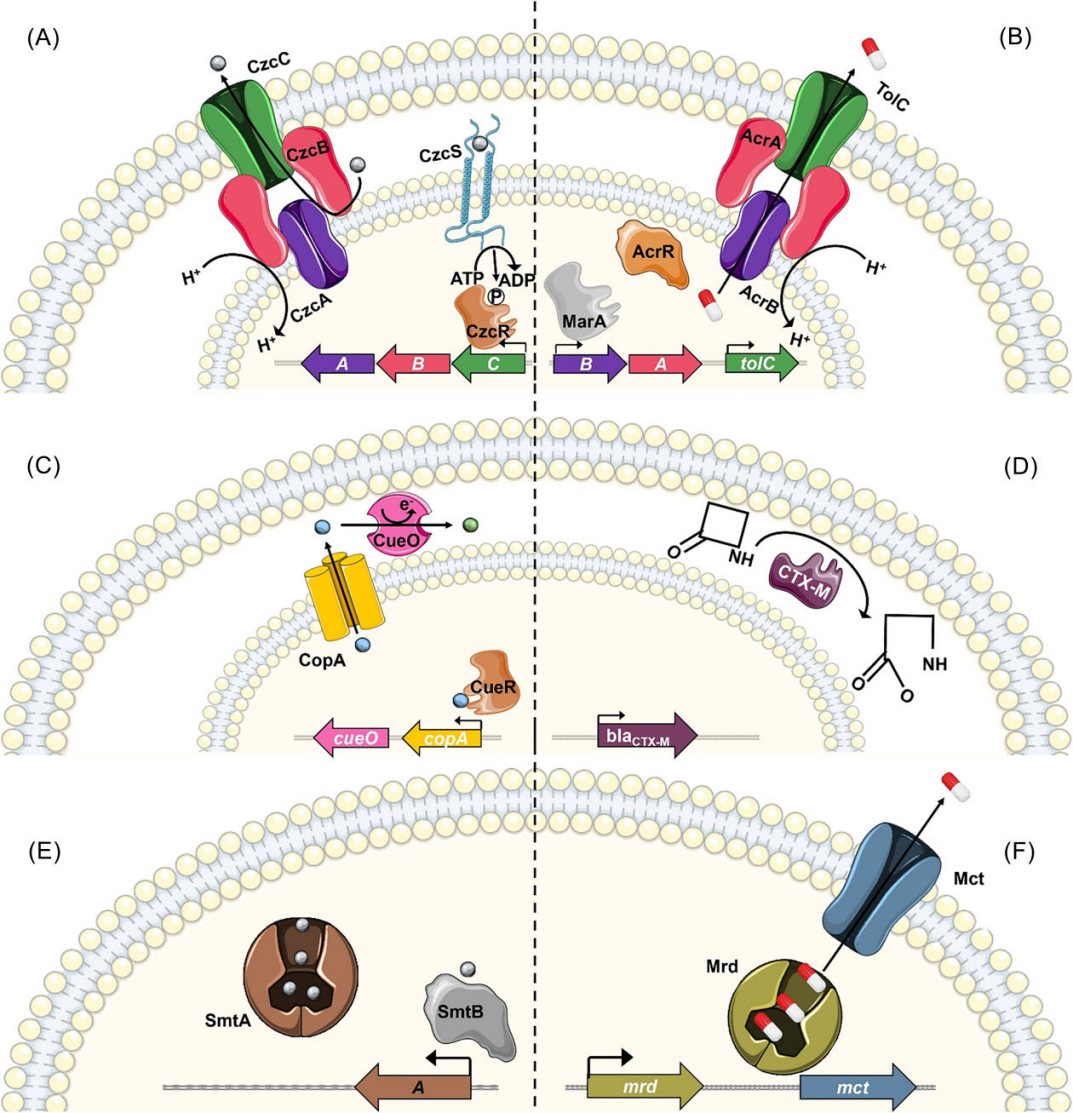
Bacterial sensing and resistance mechanisms for metals and antibiotics. (A) Metal efflux by the Czc of *Cupriavidus metallidurans* CH34. (B) Antibiotic efflux by AcrAB–TolC from *Escherichia coli*. (C) Chemical modification by Cue and Cus from *E. coli*. (D) Cleavage of β-lactam antibiotic by CTX-M in *Enterobacteriaceae*. (E) Sequestration of metal by Smt of *Synechococcus* PCC 7942. (F) Sequestration of mitomycin by Mrd, followed by export by Mct in *Streptomyces lavendulae*. Metal ions are shown as small spheres, coloured grey (Zn^2+^), blue (Cu^+^), or green (Cu^2+^). The pill symbol (red/white) represents antibiotics, specifically azithromycin, ciprofloxacin, gentamicin, and β-lactams in (B), β-lactams in (D), and mitomycin in (F). Genes are shown as block arrows with the corresponding proteins in the same colour. Icons created with smart.servier.com.

The effector proteins for MRGs and ARGs have striking similarities, although there are differences in terms of gene regulation. Efflux pumps are the most common MRG (Squadrone 2020), and are very common ARGs as well (Poole 2005). These export unwanted molecules to the cell exterior. An MRG efflux pump example is the CzcCBA resistance nodulation division (RND) divalent cation efflux pump (Nies 1995). The two-component regulator CzcRS senses cadmium, zinc, and cobalt ions, which upregulates the expression of *czcCBA* (Fig. 1A). The inner membrane transporter (CzcA), periplasmic linker (CzcB), and outer membrane protein (CzcC) are structurally homologous to the AcrAB–TolC antibiotic efflux system. AcrB forms an inner membrane transporter with the AcrA periplasmic linker to efflux antibiotics through the TolC outer membrane protein following the alleviation of AcrR repressor by global transcriptional activator MarA (Fig. 1B) (White et al. 1997, Chowdhury et al. 2019).

Chemical modification is employed as a detoxification mechanism in both antibiotic and heavy metal resistance systems, although here systems are functionally analogous rather than posing any structural homology. Ameliorating metal toxicity through reduction is limited to metals with a redox potential within the physiological range of cells (Nies 1999). For example, in the copper resistance system Cue, originally characterzied in *Escherichia coli*, sensing of Cu^1+^ causes CueR to upregulate CopA and CueO. CopA exports Cu^1+^ to the periplasm, where CueO oxidizes it to Cu^2+^ (Fig. 1C) (Rensing and Grass 2003). In contrast, antibiotics offer more diverse options for degradation or modification via hydrolysis or additions of various chemical groups. An example is the hydrolytic cleavage of β-lactams by CTX-M (Fig. 1D) (King et al. 2016).

Another method used for both metal and antibiotic resistance is intracellular or extracellular sequestration. In *Synechococcus*, the detection of high levels of zinc by the SmtB regulator results in expression of the metallothionein SmtA, which binds zinc via internal thiol groups (Fig. 1E) (Blindauer et al. 2001). An analogous system for antibiotics is the sequestration of mitomycin by Mrd protein in *Streptomyces lavendulae*. Efflux is later employed for mitomycin by the Mct transporter as there is no homeostatic need to store mitomycin in the cell as opposed to zinc and other metals (Fig. 1F) (Sheldon et al. 1999).

A final point worth noting when comparing antibiotic and metal resistance strategies in bacteria is the difference in the typical regulatory systems involved. The MRG effector genes tend to be actively regulated (induced or repressed), while some of the ARG examples above are constitutively expressed (Sheldon et al. 1997, Cantón et al. 2012). This may reflect the fact that for the metals, the cell is attempting to maintain homeostasis across environments containing variable metal concentrations, while for antibiotics, the cell is just attempting to remove all of the chemicals at all times.

## The mobilome

Many ARGs and MRGs have environmental origins (Gillings and Paulsen 2014, Larsson and Flach 2022), but how are these genes mobilized from environmental bacteria into clinical pathogens? Answering this question requires a detailed understanding of the ‘mobilome’, which is defined as the total genetic information stored in MGEs in a microbial community. MGEs are discrete sections of DNA that can transfer independent of chromosomal replication. These include mobile plasmids, insertion sequences (IS), transposons, integrons, integrative conjugative elements, and phages. The mobilome clearly overlaps with the resistome discussed above.

The speed and range of resistance acquisition in bacterial communities are largely governed by plasmids. These extrachromosomal elements transmit DNA between cells, resulting in HGT within or across taxonomic barriers including from Gram-negatives into Gram-positives (Wang et al. 2020) and niche barriers such as from environmental to clinical contexts (Martins et al. 2014, Silveira et al. 2014). This is typically achieved via conjugation (direct cell–cell plasmid transfer) (Zhang et al. 2018b), transformation (direct uptake of ‘naked DNA’) (Xu et al. 2017), or transduction (via viruses as intermediates) (Mašlaňová et al. 2016). Most clinically relevant resistance genes have been found on conjugative plasmids (Palm et al. 2022), but mobilizable plasmids (can transfer in the presence of a conjugative plasmid) and nonmobilizable plasmids (vertical inheritance only) also contribute via transformation. Resistance plasmids can carry multiple ARGs and/or MRGs, which increase the bacterial host’s ability to resist multiple antimicrobial stresses. Some chromosomal MGEs can also catalyse HGT in a similar manner to plasmids; these include genomic islands (Arsene-Ploetze et al. 2018) and integrative conjugative elements (Song et al. 2013).

Other distinct groups of MGEs have roles in the intracellular movement of resistance genes, such as IS, transposons, and integrons. These elements are abundant in bacteria, with one study reporting their prevalence in soil metagenomes as 10^−3^–10^−2^ copies per 16S rRNA gene (Zhao et al. 2019). Transposons encode transposase and/or recombinase enzymes that facilitate either cut-and-paste or copy-and-paste insertion of the whole element at new locations in the genome (Babakhani and Oloomi 2018). Integrons enable the site-specific excision, integration, shuffling, and expression of their partner elements, known as gene cassettes (Labbate et al. 2009). Class 1 integrons are very common in Gram-negative clinical isolates (Labbate et al. 2009), but they can also be readily detected in environmental bacteria, even in relatively pristine locations (Gillings et al. 2008).

While IS elements, transposons and integrons cannot self-transfer between cells, their interaction with plasmids enables this to occur. ARGs and MRGs can thus move between the chromosomes of unrelated bacteria via the agencies of multiple interacting MGEs. The case of transposon Tn*21* in plasmid NR1 provides an excellent example of genetic linkage and HGT of ARGs and MRGs (Liebert et al. 1999).

## Co-selection of metal and antibiotic resistance

### Models of co-selection

**Figure 2.**
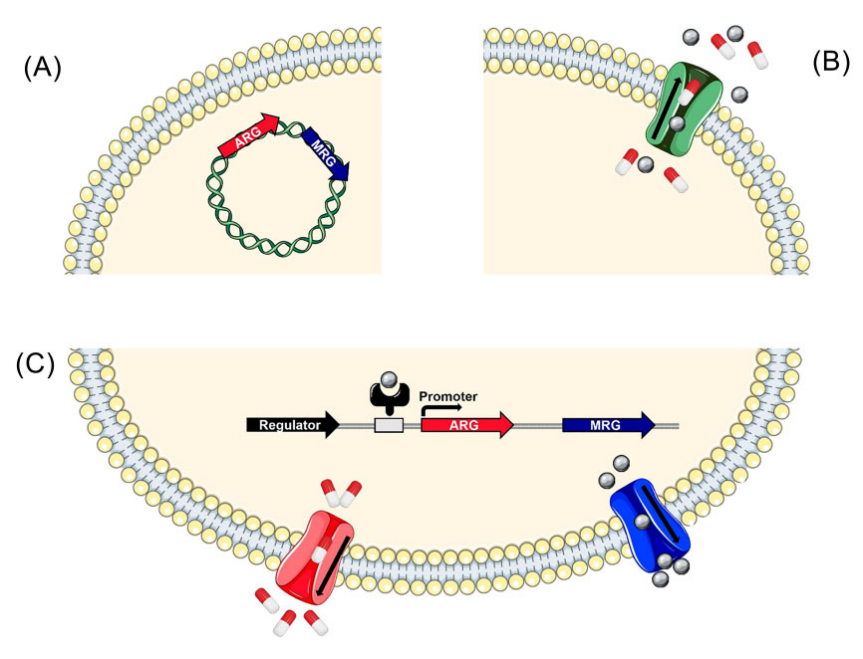
Comparison of co-selection mechanisms. (A) Co-resistance demonstrated by ARG and MRG genes residing on the same plasmid, and thus are inherited simultaneously. (B) Cross-resistance demonstrated by an efflux pump ejecting both antibiotics and metals. (C) Co-regulation demonstrated by an ARG and MRG expressed together after induction by a metal. Grey spheres represent metal ions, and red and white pills represent antibiotics. Icons created with smart.servier.com.

As mentioned prior, there are three recognized co-selection models: co-resistance, cross-resistance, and co-regulation. Co-resistance involves resistance genes being physically linked, most often on the same MGE, resulting in simultaneous inheritance as one package (Fig. 2A). Cross-resistance involves a single mechanism conferring resistance to both agents, for example an efflux pump removing both antibiotics and metals (Fig. 2B). Finally, co-regulation involves resistance genes that share a promoter or regulator, resulting in a unified transcriptional response (Fig. 2C) (Baker-Austin et al. 2006). These mechanisms are not mutually exclusive. Any of these co-selection methods could allow ARGs, and therefore resistant phenotypes to persist under the selection of heavy metals regardless of antibiotic exposure.

**Figure 3.**
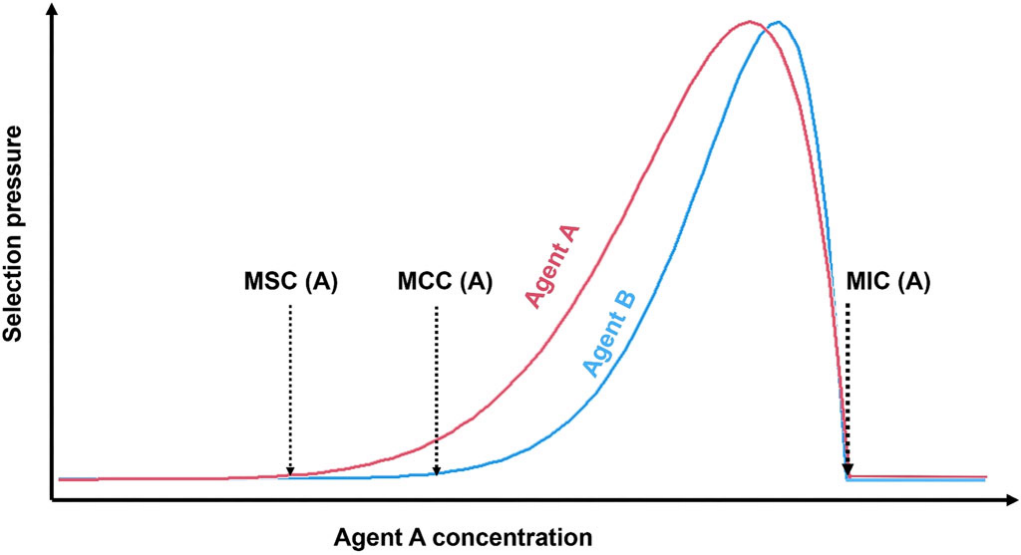
Relationships between MIC, MSC, and MCC as a function of concentration. Selection pressure from agent A increases with concentration from the MSC until it approaches the MIC, after which it rapidly declines due to cytotoxicity. Co-selection pressure on agent B increases from the agent A MCC until the MIC is reached.

Some relevant concepts for understanding co-selection between metals and antibiotics include the MIC, the minimum selective concentration (MSC), and the minimum co-selective concentration (MCC). The MIC is the lowest concentration of an agent that inhibits the growth of a specific microbe (Seiler and Berendonk 2012). The MSC is the lowest concentration of an agent, where at which the fitness benefit of resistance outweighs the fitness cost (Yu et al. 2017). The MCC is the lowest concentration of one agent that will select for resistance towards another agent (Arya et al. 2021). There are very few examples of defined MSC or MCC values, but in theory, they should be equal to or lower than MIC values. This is significant since although metals or antibiotics rarely reach the MIC in the environment (Wales and Davies 2015, Zhang et al. 2018b), they likely exceed the MSC (Gullberg et al. 2014, Zhang et al. 2018b) or MCC (Seiler and Berendonk 2012, Song et al. 2017). The MSC and MCC values will vary greatly depending on the combination of agents, the type of microbial community and the environmental conditions (Gullberg et al. 2014, Arya et al. 2021). The qualitative relationships between MIC, MSC, and MCC are shown in Fig. 3.

## Methodological advances in the study of co-selection

The methodology used to study co-selection over the years has become vastly more sensitive and scalable. The first evidence for co-selection between heavy metals and antibiotics was published in 1964 and described the co-transduction of penicillin and mercury resistance in *Staphylococcus*, such that the vast majority of transductants under penicillin selection also acquired mercury resistance, and vice versa (Richmond and John 1964). Since then, many studies using diverse methods (genetic mutation, transformation, and conjugation) have revealed examples of linked antibiotic and metal resistance in different bacterial lineages including *Salmonella* (Ghosh et al. 2000), *Enterococcus* (Hasman and Aarestrup 2002), and *Burkholderia* (Hayashi et al. 2000). This led to the seminal review by Baker-Austin et al. (2006), which recognized the importance of metals in antibiotic co-selection and outlined the co-selection models, but the relative contributions of particular co-selection mechanisms in specific environments is not clear, and requires further study.

**Figure 4.**
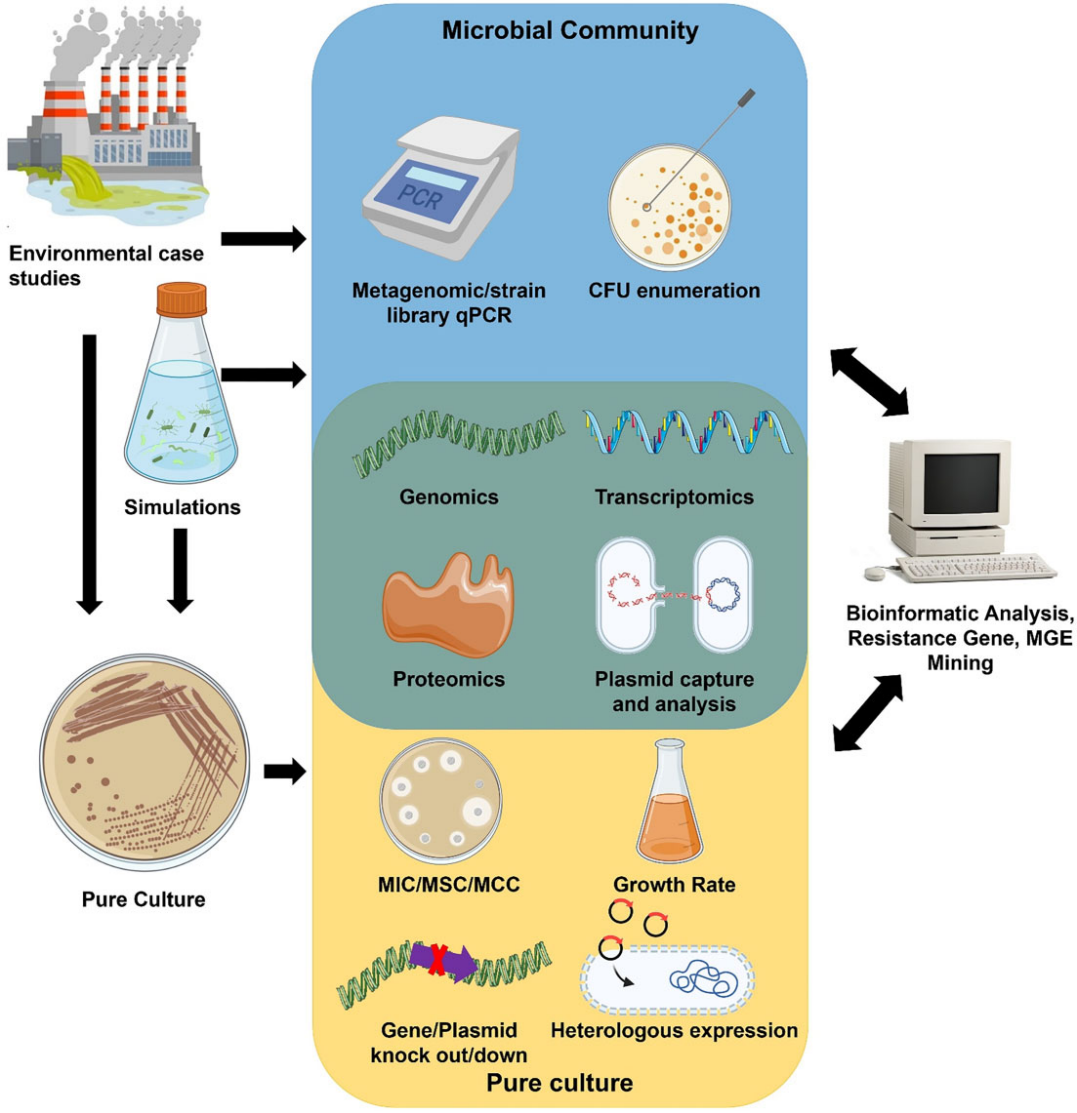
Methodologies for studies investigating co-selection. Environmental samples or environmental simulations (microcosms and mesocosms) are sources of communities or pure cultures for study. qPCR measures relative gene abundance, while the culturable resistant isolates can be enumerated via plating. Pure cultures can have MIC, MSC, or MCC profiled through disk diffusion, microbroth dilution, or E tests. Growth rates and yields under varying selective pressures can be quantified. Gene knockouts or knockdowns can assign genotypes to phenotypes. The occurrence and genetic context of resistance genes can be revealed through DNA sequencing, while expression levels of these genes can be tested via transcriptomics, RT-qPCR, microarrays, proteomics, enzyme assays, ELISA, or western blots. Plasmid capture and/or plasmid curing experiments can help to infer the function of plasmid-encoded genes. Finally, bioinformatic data can be mined for resistance genes and MGEs, which in turn informs experimental characterization. Icons were created with BioRender.com and smart.servier.com.

Advances in molecular biotechnologies such as high-throughput DNA sequencing have enabled a more mechanistic understanding of co-selection. The genetic basis of co-selection can now extend beyond genetically characterized lab strains to the culture-independent analysis of whole microbial communities (Li et al. 2020, Huang et al. 2023), with simultaneous quantification of hundreds of resistance genes (Mazhar et al. 2021, Huang et al. 2022), and in-depth characterization of the mobilome (Kothari et al. 2019, Perez et al. 2020). Greater sequencing capabilities have massively expanded bioinformatic databases focused on resistance genes and MGEs. These databases include CARD (McArthur et al. 2013), ResFinder (Florensa et al. 2022), MEGARes (Lakin et al. 2017), ARG-ANNOT (Gupta et al. 1999), ARDB (Liu and Pop 2009), Resqu (Resqu 2023), BacMet (Pal et al. 2014), AMRFinder (Feldgarden et al. 2021), ACLAME (Leplae et al. 2004), INTEGRALL (Moura et al. 2009), ISFinder (Siguier et al. 2006), and PlasmidFinder (Carattoli et al. 2014). These databases are invaluable for informing subsequent hands-on research, e.g. gene knockouts (Wang and Fierke 2013, Bischofberger et al. 2020), heterologous expression (Conroy et al. 2010, Shi et al. 2019), and plasmid capture assays (Fig. 4) (Wang et al. 2020, Pu et al. 2021).

Metagenomic data on ARGs, MRGs, and MGEs has allowed new insights into co-selection. For example, Pal et al. (2017) raised again the important question of how resistance genes are often maintained in the absence of apparent selection, and they challenged the importance of metals as factors driving HGT rather than other inherent properties of the MGEs themselves. More manipulative simulations of heavy metal exposure (such as microcosms) were recommended to delve into mechanisms that would counterbalance the abundance of environmental case studies. The environmental origin of resistance genes and the selective effects of subinhibitory levels of metals are now well known, and this One Health understanding has been used to propose methods of clinical intervention (metal chelators and efflux blockers) and site rehabilitation (bioremediation or biosorption) (Vats et al. 2022).

## Evidence for co-resistance

**Figure 5.**
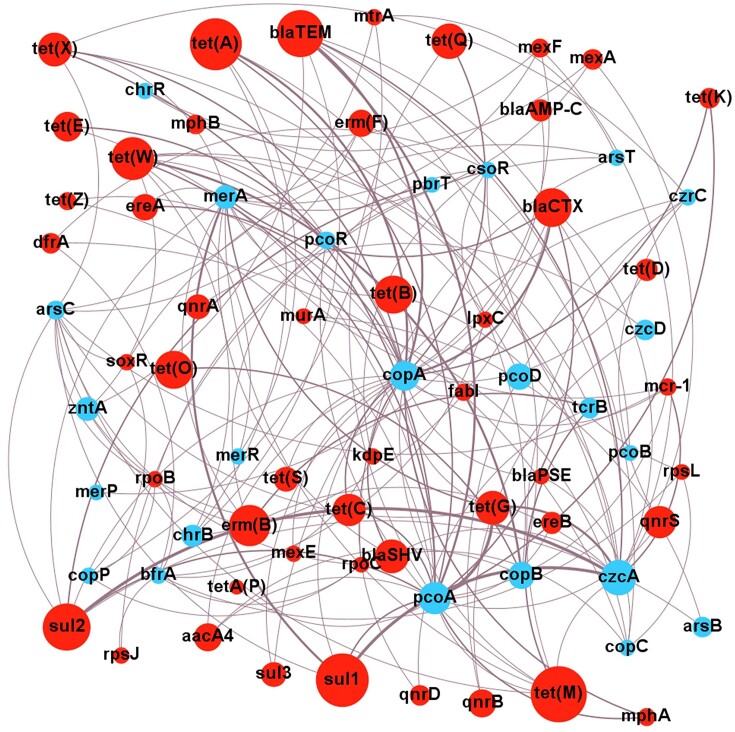
Network analysis of correlations between MRGs and ARGs. Analysis based on correlations of ARGs and MRGs from co-selection studies in environmental contexts from 2010 onwards. Size of nodes is proportional to the number of primary studies investigating that specific resistance gene. Weight of edges is proportional to the number of studies finding a positive correlation. Only positive correlations are shown. Blue indicates ARG, and red indicates MRG. Network visualized with Gephi v 0.9.7.

Most evidence for the co-resistance of antibiotics and metals is correlational. Finding ARGs in close proximity to MRGs in a genome is consistent with these genes being selected as a combined entity. The co-resistance model also implies a vehicle of resistance gene transfer, so correlations with integrons, transposons, or plasmids lend support to the model. Although correlational evidence is far from conclusive, it is a good starting point to reveal what the likely relevant ARG and MRG gene combinations are that warrant closer attention. To help identify these ARG and MRG combinations of special interest, the correlations from the literature (Table S1) have been presented as a metagenomic network analysis (Fig. 5).

The network analysis emphasizes consistent connections between *czcA* and *sul1, sul2*, or *tet*(G); *copA* or *pcoA* and *bla_TEM_; merA* and *sul1*; and *pcoA* and *tet*(G). These combinations warrant further mechanistic investigations to confirm co-resistance since all these studies are impacted by biases of sampling and study design, such as the environments sampled, the culturing and/or molecular methods used, and the antibiotic or metal agent(s) chosen for study. Particular resistance genes may be more numerous across the bacterial community, or are easier to study, both of which would result in positive correlations. As an example, consider the correlation of *merA* and *sul1* (Fig. 5); while *merA* homologues are found in both Gram-positives and Gram-negatives, they are strongly correlated with *sul1* only in Gram-negatives (Sköld 2001, Christakis et al. 2021). Thus, a study on a Gram-negative collection is likely to find this correlation, whereas a wider study may not. Proof of co-resistance ideally requires the experimental introduction of MRGs and ARGs into recipient test bacteria. This functional confirmation is important, especially for MRGs, many of which may serve homeostatic rather than resistance functions.

### Observational studies of co-resistance

#### Pristine environments

ARGs and MRGs are common in environmental bacteria, even in environments with minimal anthropogenic exposure. In metal-enriched water surrounding deep-sea hydrothermal vents, sequenced plasmids carrying MRGs were prevalent, and many of these plasmids also carried ARGs, including β-lactam and fluoroquinolone resistance genes (Farias et al. 2015). At least one of these deep-sea resistance plasmids possessed conjugation genes, indicating strong potential for HGT, which was also supported by a phylogenetic comparison of the strains. Metagenomic studies of naturally metal-rich high-altitude lakes in the Himalayas (Sharma et al. 2022) and Andes (Perez et al. 2020) have also yielded extensive correlations between MRGs and ARGs, with relative abundance comparable to urban wastewater. In a survey of subsurface soils (>1 m depth) from diverse locations including both pristine and contaminated environments, high concentrations of metals (copper, lead, and zinc) increased ARG and MRG relative abundance and diversity more than any other factor (Wang et al. 2021b). A linear positive correlation of MRGs with ARGs was seen, consistent with associated inheritance. These studies collectively support the hypothesis that environments that are naturally rich in metals select for MRGs and ARGs but require further validation for co-resistance within a single organism.

#### Agricultural environments

Fertilized (either manure or inorganic-based) agricultural soils contain both heavy metals and antibiotics (Yu et al. 2017), and so are potential hot spots for co-selection. The agricultural context is also critical for study from a One Health perspective since this is a location where plants, animals, humans, and their feeds and wastes intersect. The metagenomic total ARG distribution in manure-fertilized soil at poultry farms was influenced by metals, particularly cadmium, with 5.7% of ARG variance explained due to metals alone and 32% by metals in combination with other factors (Mazhar et al. 2021). Notably, these numbers are higher than the ARG variances attributed to antibiotics (0.7% alone and 10% combined with other factors). The co-resistance model was supported in the poultry farm study via the frequent correlation of ARGs with MGEs (*intI1*, IS*613*, and Tn*24*). Copper-enriched soils from olive tree farms showed statistically significant co-occurrences of resistance genes to copper, tetracycline, and β-lactams and *zntA* co-occurring with *tet*(C) in recovered isolate genomes (Glibota et al. 2020). The exact genomic context was not provided here, but this is a step up from metagenomic correlations as it was established that these genes reside in the same genome. Furthermore, *intI1* was present in resistant isolate genomes, offering a potential mechanism for the acquisition of the resistance genes. In livestock wastewater, metagenomic qPCR uncovered a strong correlation between the relative abundance of *copA* and plasmid-borne *mcr-1*, which is consistent with these genes being on the same plasmid (Yuan et al. 2018).

#### Industrial environments

Industrial environments are typically metal-enriched but devoid of antibiotic pollution (with the notable exception of the pharmaceutical industry). These heavily impacted environments have provided many opportunities to study the effects of metals on ARGs, MRGs, and MGEs. Mining is an acute source of heavy metal contamination and may promote the mobilization of resistance genes. In soil impacted by a gold mine, metagenomic community qPCR found that ARG abundances were correlated to levels of copper, manganese, nickel, and zinc (Yan et al. 2020). This and another study (Huang et al. 2023) revealed that *Actinomycetota* and *Pseudomonadota* were associated with a range of ARGs, MRGs, and MGEs (IS*26, istA5, tnpA2*, and ISRj1), which is consistent of co-transfer of ARGs and MRGs by HGT. One river polluted with mine tailings yielded a *Lysinibacillus* isolate with plasmid-borne copper and streptomycin resistance (Chihomvu et al. 2015). This assessment was made through curing experiments rather than plasmid sequencing, so details of the involved plasmids were not determined. One interesting metagenomic study used a clone library approach with DNA from an acid mine drainage site to find a DNA fragment that conferred antibiotic resistance in *E. coli* (Arsene-Ploetze et al. 2018). This metagenomic DNA encoded a CusAB-like efflux pump that increased the MIC for gentamicin, kanamycin, and rifampicin upon heterologous expression in *E. coli*. The authors could not confirm that this gene also facilitated copper resistance, so it is unclear whether this is an example of co-resistance or cross-resistance.

Heavy metal contamination from industries other than mining also facilitates metal co-resistance. Lake sediment cores from a heavily industrialized area in the UK found a correlation between sedimentary zinc concentration and the proportion of culturable isolates with zinc resistance, oxacillin resistance, and trimethoprim resistance (Dickinson et al. 2019). These resistant phenotypes were additionally correlated to metagenomic *intI1* abundance measured via qPCR, suggesting integron involvement. In electroplating wastewater, whole genome sequencing of a nickel-resistant *Shewanella* sp. revealed the close proximity of IS elements with nickel and cobalt MRGs in addition to *blaR1* and erythromycin ARGs (Cai et al. 2019). A clear example of plasmid-mediated co-resistance was seen in a *Pseudomonas aeruginosa* isolate obtained from freshwater near chemical industries. This isolate carried a conjugative plasmid carrying tetracycline and copper resistance genes *tet*(A), *copA*, and *copB* (Martins et al. 2014), which was able to conjugate into *E. coli*.

Wastewater treatment plants and sewage sludge have been heavily studied for their role in propagating MRGs, ARGs, and MGEs, and are another hotspot for co-selection. Positive correlations of MRGs with ARGs and MGEs in wastewater are common (Lin et al. 2019, Murray et al. 2019), with *intI1* correlated with *czcA, sul2, arsB* (Di Cesare et al. 2016b), and *mpbH* (Yuan et al. 2018) revealed by metagenomic qPCR. Integrons are correlated with MRGs at many sites, but these correlations must be interpreted with care. MRGs have never been seen as integron gene cassettes, the more likely hypothesis is that integrons and MRGs are carried by the same transposons and plasmids.

Consistent correlations of MRGs with ARGs and MGEs have been observed across entire catchment systems. The Xiangjiang river in China has been investigated by several studies measuring ARGs at mining discharge points (Xu et al. 2017, 2019). Bacteria isolated from the river contained numerous β-lactamase genes and MRGs, and their MIC for ampicillin increased up to eight-fold near the mining discharge points (Wang et al. 2021a). Two co-resistance plasmids were discovered in *Bacillus megaterium* and *Shewanella oneidensis*. One plasmid contained *czcD, qnrA*, and *qnrB* and the second contained *copB, merR, tet*(A), and *tet*(W) (Xu et al. 2017). In a metagenomic qPCR study of Indian and UK metal-impacted waters and sediments, total metal concentration was positively correlated to MRGs, ARGs, and integron relative abundance, to the extent that it explained 83% of resistance gene distribution (Gupta et al. 2022), and 92% of bacterial community composition (Gupta et al. 2023). In those studies, network analysis of metagenomic qPCR data suggested that resistance genes *tet*(W), *bla_TEM_, mefA, zntA*, and *chrA* were likely residing in the same bacterial host, but the nature of any MGEs involved is unknown. Genome analysis of a *Comamonas* isolate from Melbourne sediment revealed a class 1 integron and kanamycin gene cassette adjacent to a chromate resistance transposon. The sediment metagenome strongly correlated class 1 integrons with heavy metals (Rosewarne et al. 2010). This is a nice example of the interplay of different MGEs, ARGs, and MRGs that can be involved in co-selection.

One unusual industrial environment notable for co-resistance is dense atmospheric urban smog. In a fascinating study by Pal et al. (2016), the metagenomic diversity of both ARGs and MRGs in smog from Beijing was found to be higher than in any other sampled environment. The abundance of ARGs and MRGs was equivalent to wastewater, which is generally considered to be the most significant hotspot for resistance gene spread. Based on the high abundances and diversity of MRGs and ARGs detected, including notable carbapenem ARGs, the smog microbiome seems likely to be another important location where co-resistance in bacteria can arise.

### Manipulative studies of co-resistance

#### Microcosms

Characterization of the resistome and mobilome before and after controlled heavy metal or antibiotic exposure in microcosms allows the gathering of more rigorous evidence for co-resistance than correlations and genomic sequencing. One striking finding to emerge from such studies is that high concentrations of metals can promote HGT, and this effect can manifest rapidly. For example, relative proportions of antibiotic-resistant bacteria in a biofilter community increased after just 6 hours of copper (100 mg l ^−1^) exposure. In addition, metagenomic qPCR revealed an increase in the relative abundance of *cusCBA, tet*(B), *tet*(G), *tet*(L), *mexF, sedB* transposons, and integrases (Zhang et al. 2018a). Although cross-resistance was suggested by the authors, there was no evidence for copper-export genes other than *cusCBA*, so the responsible mechanism is still under question. Concerningly, in this study, acquired resistance to vancomycin, erythromycin, and lincomycin was maintained in the absence of selective agents for at least seven days.

Studies using combinations of metals and antibiotics can give useful insights on possible synergy but are unfortunately rare in the literature. In wastewater microcosms exposed to copper or zinc (1 mg l^−1^) plus tetracycline or ampicillin (0.5 mg l^−1^), several ARGs and MRGs increased in absolute abundance under all exposure combinations. Metagenomic qPCR revealed that the transposase gene *tnpA* increased in absolute abundance and was strongly correlated with resistance genes *copA* and *chrB*, which may mean these MRGs are carried by transposons (Zhao et al. 2021). This study also linked specific bacterial types to specific MRGs and ARGs through correlational network analysis, with one notable candidate being *Mycobacterium* spp. carrying *bla_TEM_, copA, copB, pcoD*, and *zntA*. Experiments using microcosms of wetland sediments demonstrated through metagenomic qPCR that doxycycline (50 mg l^−1^) and cadmium (0.5–5 mg l^−1^) exposure for 3 months increased MRG and ARG relative abundance more than either agent on their own (Yu et al. 2022). In this study, both agents were positively correlated with individual resistance genes and bacterial genera, with *Acinetobacter* being one notable predicted host for MRGs, ARGs, and integrons. Both of these studies reveal clinically important genera possessing MRGs and ARGs.

Microcosm studies of wastewater or sludge have revealed close relationships between metals, ARGs, and MRGs. A common finding in bacterial communities is that heavy metals (Tan et al. 2023) or MRGs (Zhang et al. 2019a) have a much greater impact than antibiotics on the abundance and types of ARGs, although community composition is typically the most dominant factor in determining ARG types (Zhang et al. 2016, 2017). The relative abundance of ARGs and MRGs in the metagenome of wastewater microcosms have been correlated to *intI1* via qPCR, revealing *intI1* correlations with both ARGs and MRGs, again consistent with co-resistance (Zhang et al. 2016, 2019a, Tan et al. 2023). In soil microcosms, 10 mg kg^−1^ sulfamethoxazole was determined to be the MSC for MRGs, ARGs, and MGEs at a community level (Li et al. 2023). In that study, each MGE was correlated with multiple ARGs, consistent with co-carriage.

#### Studies with plasmids

Plasmids are the primary transfer vehicle for MRGs and ARGs, and co-residence of ARGs and MRGs on the same transferable plasmid is a mechanism of co-resistance. Furthermore, metals can stimulate plasmid transfer. The frequency of conjugation of heavy metal and antibiotic co-resistance plasmids from *B. megaterium* (Xu et al. 2017) or *Pseudomonas monteilii* (Wang et al. 2021a) into *E. coli* HB101 increased by one to two orders of magnitude in the presence of copper (0–20 µg l^−1^) or zinc (0–30 µg l^−1^). In a clear display of acquired co-resistance, the recipient *E. coli* gained resistance to both heavy metals and antibiotics upon transformation or conjugative uptake of the plasmid (Xu et al. 2017, Wang et al. 2021a). In one of the few studies investigating the MSCs for both metals and antibiotics, *E. coli* MG1655 hosting a 220-kb extended spectrum β-lactamase (ESBL) resistance plasmid was exposed to subinhibitory levels of metals and antibiotics (Gullberg et al. 2014). When the bacteria were exposed to various combinations of arsenite, tetracycline, and trimethoprim, the MSC decreased with all agents when compared to individual exposure. The reported MSCs were at environmentally relevant concentrations, giving weight to the argument that even trace residues of antimicrobials may potentially have profound impacts on selection.

Genotypic or phenotypic verification of plasmid recipients demonstrates co-resistance in action. The transferability of resistance to mercury, β-lactams, and quinolones was examined in ESBL-containing isolates from the Yamuna River in New Delhi (Siddiqui et al. 2020). Conjugation from the arsenic and mercury-resistant isolates into *E. coli* revealed simultaneous acquisition of metal and antibiotic resistance, with transconjugants receiving ESBL, *qnrS, merB, merP*, and *merT*. Transfer of ARGs and MRGs together has also been observed in Gram-positives, such as in *Enterococcus* lab strains which acquired *tcrB, cueO, aadE, erm*(B), *tet*(L), *tet*(M), and *vanA* from mating assays with copper resistant environmental *Enterococcus* spp. (Silveira et al. 2014). Subsequent transconjugants inherited phenotypic resistance to both copper and multiple antibiotic families. In plasmid capture experiments, it is important to phenotypically verify resistance acquisition, since the transfer of an ARG or MRG alone does not guarantee a resistance phenotype. For instance, a multiresistance plasmid carrying *copAB* in *P. aeruginosa* did not confer copper resistance to *E. coli*, despite detection of *copAB* in the transconjugants (Martins et al. 2014). Although these authors did not probe this finding, it could be due to the multigene nature of the *cop* system, assuming the other genes required (*copC* and *copD*) (Pal et al. 2017) were not co-transferred with *copAB*.

### Bioinformatic studies of co-resistance

The expanding size of genome databases gives us increasing power to analyse the relationships between MRGs and ARGs. In one study of 2522 complete genomes from the 2014 NCBI bacterial genome database (Pal et al. 2015), 17% contained both ARGs and MRGs and genomes containing an MRG were 10-fold more likely to contain an ARG compared to those with no MRGs. Two years later, the same database contained 5436 complete bacterial genomes, with half containing both ARGs and MRGs (Li et al. 2017). This seemingly dramatic shift is largely due to the increased representation of clinically significant taxa (especially *Enterobacteriaceae*) in bioinformatic databases. The multiresistance genomes were found much more often in human or animal microbiota and pathogens rather than in the environment (Pal et al. 2015, Li et al. 2017). Based on analysis of the 2014 NCBI database, 5% of plasmids contained both ARGs and MRGs (Pal et al. 2015), with those resistance plasmids tending to be large (median 76 kb), conjugative, and contain toxin–antitoxin systems, indicating that they can both self-transfer and fix themselves into new hosts.

Mining of genome databases suggests that MRG–ARG pairings are frequent and that some specific pairs of MRG–ARG are more common than others. This favours co-resistance over cross-resistance as a mechanism, because in cross-resistance, the expectation would be that a few individual genes with a broad substrate range would dominate. Instead, it seems that groups of diverse genes with more specific activities have been assembled. The most common associations seen in chromosomes are mercury MRGs with aminoglycoside, phenicol, sulfonamide, and tetracycline ARGs (Pal et al. 2015); zinc MRGs with β-lactam, bacitracin, and polymyxin ARGs; copper MRGs with β-lactams, kasugamycin, and bacitracin ARGs; and arsenic MRGs with β-lactam, bacitracin, and fosfomycin ARGs (Li et al. 2017). Some of these correlations differ from those shown in Fig. 5. This is likely due to the different states of the literature and databases in 2016 versus 2023. Nonetheless, many similar dominant correlations are seen, such as mercury ARGs with sulfonamide and tetracycline ARGs; copper MRGs with β-lactam ARGs; and zinc MRGs with β-lactam and polymyxin ARGs.

In the context of MGEs, recurring associations of MRGs and ARGs seen in plasmids include the cadmium resistance gene *cadD* with aminoglycoside or macrolide resistance genes; mercury resistance genes with sulfonamide, aminoglycoside, β-lactam, phenicol, or trimethoprim resistance genes; and bacitracin with copper or zinc resistance genes (Pal et al. 2015, Li et al. 2017). Integrons are more associated with MRG and ARG pairs than transposons (Li et al. 2017). Integron and IS*CR* genes are on 10% and 7% of plasmids carrying resistance genes, respectively, and *intI1* and IS*CR* had a strong correlation with mercury, aminoglycoside, phenicol, sulfonamide, and tetracycline resistance genes (Pal et al. 2015).

## Evidence for cross-resistance

**Figure 6.**
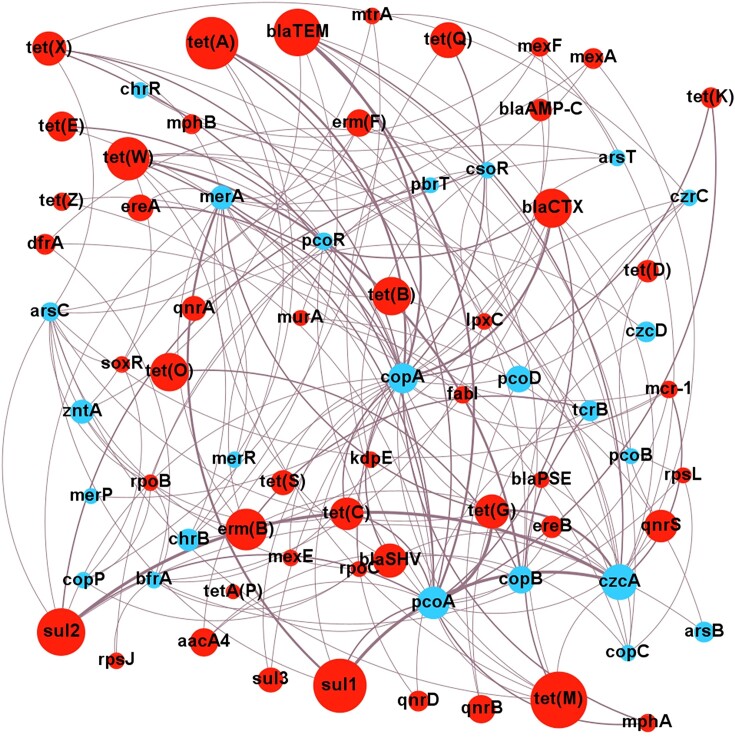
Network analysis of correlations between heavy metals and ARGs. Analysis based on correlations of ARGs and MRGs from co-selection studies in environmental contexts from 2010 onwards. Size of nodes is proportional to the number of primary studies investigating that specific resistance gene. Weight of edges is proportional to the number of studies finding a positive correlation. Only positive correlations are shown. Blue indicates ARG, and red indicates heavy metal. Network visualized with Gephi v 0.9.7.

Cross-resistance exists where a single mechanism provides resistance to both a metal and antibiotics. Robust evidence for cross-resistance can be derived from experimental characterization in pure cultures, but the majority of studies lack this level of rigour and just report correlations. The limited functional information about most open reading frames in genome databases further exacerbates this issue. Despite these problems, we can still navigate a useful path by being guided by correlational studies as indicators for which metal/antibiotic/ARG/MRG combinations deserve more detailed mechanistic investigation. Zinc and copper resistances dominate studies of cross-resistance, with consistent correlations of these metals to *aacA4, bla_OXA_, bla_TEM_, erm*(B), *erm*(F), *sul1, sul2, tet*(M), and *tet*(W) (Fig. 6). Other metals which correlate highly to ARGs include arsenic, cadmium, chromium, manganese, nickel, and lead. As expected, metal and ARG correlations are not universal, and some yield negative correlations, so cross-selection is limited to certain chemical and enzyme combinations. Further indirect evidence for cross-resistance comes from the predominance of broad-spectrum efflux pumps in the resistome (Thomas et al. 2020, Liu et al. 2021, Wang et al. 2021b, Furlan et al. 2022), these provide efflux of multiple diverse targets.

Further speculative evidence for cross-resistance comes from the many studies that report discrepancies between detected resistance genes and the resistance phenotypes. Some of these may represent cases where resistance genes have a broader range of action than previously documented. For example, in a study on marine biofilms growing on boat hulls treated with copper and zinc-based paint, tetracycline-resistant colonies from the metal-treated surfaces were significantly more numerous compared to nontreated surfaces despite a reduction in the relative abundance of *tet* genes (Flach et al. 2017). In this study, metagenomic sequencing revealed copper and zinc MRGs did increase four–five-fold, which raises the question whether the MRGs may be responsible for the tetracycline resistance. Similar unexplained metal resistance phenotypes, where ARGs have been detected, but not the expected MRGs also occur in the literature (Vignaroli et al. 2018, Zhou et al. 2019, Jia et al. 2021). These cases may also indicate the presence of novel resistance genes that have yet to be identified. There are still many hypothetical proteins even in well-characterized genomes like *E. coli* that await assignation of functions, and the proportion of these hypothetical open reading frames is higher yet on MGEs (Hatfull 2008).

### Molecular genetic studies in pure cultures

More robust evidence from cross-resistance stems from heterologous expression or knockout methods, which link specific resistance genes to phenotypes. Making knockouts in environmental bacteria is not easy, and expression in standard hosts has its own technical difficulties (Rosano and Ceccarelli 2014, Kaur et al. 2018), thus, only a few studies have taken these more rigorous molecular approaches for studying cross-resistance, as described below. While a variety of metal and antibiotic cross-resistance mechanisms exist, efflux pumps seem to play the largest role.

One efflux system known to provide cross-resistance is the RND pump MdtABC. Copper and zinc induce this efflux system, which is involved in the export of antibiotics (β-lactams, novobiocin) and metals (copper and zinc) in *Salmonella* and *E. coli* as demonstrated via by an MIC alteration following gene overexpression or deletion (Nishino et al. 2007, Wang and Fierke 2013). This was confirmed by the significantly higher intracellular zinc ion content following *mdtA* or *mdtC* deletion compared to the wild type (Wang and Fierke 2013). Other known or likely cross-resistance pumps identified by knockout methods include MacAB in *Agrobacterium tumefaciens*, the deletion of which caused an accumulation of intracellular arsenite and a 2–16 fold MIC reduction to erythromycin, various penicillins and arsenite (Shi et al. 2019). As another example, deletion of efflux transporter MdrL in *Listeria monocytogenes* decreased MICs 2–10-fold towards erythromycin, josamycin, clindamycin, cefotaxime, cobalt, chromate, and zinc (Mata et al. 2000).

Heterologous expression also provided evidence for cross-resistance in the case of MacAB above (Shi et al. 2019), with expression of this gene in *E. coli* giving a two-fold MIC increase to macrolides, penicillins, and arsenite. Similarly, heterologous expression of the GesAB gold efflux pump from *Salmonella* in *E. coli* significantly increased MICs to phenicols and β-lactams (Conroy et al. 2010). Overexpression of the *E. coli* copper and silver RND efflux pump system, *cusCFBA*, provided three-fold enhanced resistance to fosfomycin (Nishino and Yamaguchi 2001) and moderate resistance to sulfamethoxazole (Conroy et al. 2010). Thus, data from many bacterial genera confirm that there are diverse efflux pump systems that give resistance to both antibiotics and metals.

Other cross-resistance mechanisms include extracellular polysaccharide production and enzyme-mediated detoxification. The extracellular polysaccharide produced by *Enterobacter cloacae* P2B can sequester both metals and antibiotics (Naik et al. 2012), resulting in multiresistance to lead, cadmium, mercury, β-lactams, macrolides, chloramphenicol, and sulfamethoxazole–trimethoprim. This polysaccharide production is comparable to the chemical agent protection offered by biofilms, and hence biofilm production can also be viewed as a form of cross-resistance. Few chemical modification enzymes have shown evidence for cross-resistance, but one example is DsbA–DsbB, a periplasmic disulfide bond oxidoreductase. Deleting this enzyme system in *Burkholderia cepacia* decreased their MIC to cadmium, zinc, and a range of antibiotics (Hayashi et al. 2000). It is possible that this effect of DsbA–DsbB is because both cations and antibiotics may react with thiol residues, however, this mechanism was not verified.

## Evidence for co-regulation

Co-regulation is the least frequently reported metal and antibiotic resistance mechanism (Bazzi et al. 2020). This mechanism is indirect, where exposure to one agent triggers regulatory events that result in resistance to another. Many instances of co-regulation also encompass elements of co- and cross-resistance. Due to this complexity, determining a co-regulatory mechanism of resistance is difficult with metagenomic or microcosm data and requires pure culture manipulations.

### Co-regulation in pure cultures

In *E. coli* MG1655, exposure to zinc (0.2–1 mM) or copper (2 mM) led to a global regulatory response affecting 122 genes, with BasRS (Lee et al. 2005) and BaeRS (Nishino et al. 2007) as potential global regulators. Upregulated genes included the efflux pumps *mdtABC*, discussed above also in the context of cross-resistance. Exposure of *P. aeruginosa* to zinc (5 mM) activated the CzcR–CzcS regulatory system, which led to both the expression of the efflux pump CzcCBA and the reduced expression of membrane porin OprD, with the latter conveying carbapenem resistance (Perron et al. 2004).

Comparison of the growth rates of cultures exposed to metals, antibiotics, and combinations implies co-regulation if the metal ameliorates the inhibitory growth effects of the antibiotic. One study that took this approach with an *Enterobacteriaceae* isolate found that growth was faster in media containing tetracycline plus subinhibitory arsenate, copper, or zinc compared to tetracycline alone, and arsenate was found to induce the *tet*(34) and *emrD* genes (Chen et al. 2015). Another kind of co-regulation can arise if metals stimulate general stress responses that minimize the toxicity of the antibiotic. Evidence for this comes from studies with *Pseudomonas fluorescens*, which exhibited higher growth rates in media containing cefradine (1 mg l^−1^) and zinc (<160 mg l^−1^) than with cefradine alone (Xu et al. 2015a). This was proposed to be due to differences in reactive oxygen species management in the cultures, including superoxide dismutase (Xu et al. 2015a) and nitric oxide synthase activities (Xu et al. 2015b). Care must be taken to differentiate co-regulation from other kinds of antagonism between metal and antibiotics. For example, zinc acts as a cofactor for metallo-β-lactamases (Gupta et al. 2023).

### Plasmid-focused studies of co-regulation

One intriguing area of study has been the impact of metals and antibiotics on the movement of MGEs, e.g. conjugation. This represents an intersection of co-resistance and co-regulation. The response of *E. coli* to metals appears to facilitate higher conjugation rates via increased cell membrane permeability and the upregulation of Omp porin proteins (Zhang et al. 2018b, 2019b, Wang et al. 2020, Pu et al. 2021). In addition, stress response genes including the SOS pathway, *soxRS* and *oxyR* are upregulated in response to metal exposure. This is an interesting observation since such systems are also inducers for (or induced by) MGE movement, such as in conjugation and integron recombination (Guerin et al. 2009, Baharoglu et al. 2010).

The broad host range 60 kb IncP-1α antibiotic resistance plasmid RP4 (also known as R18, R68, RK2, and RP1; Pansegrau et al. 1994) has been used for studying the effect of metals on conjugation. Transfer of RP4 is stimulated by environmentally relevant levels (10–300 µg l^−1^) of zinc, chromate, silver, and copper in both *E. coli* (Zhang et al. 2018b) and *Pseudomonas putida* (Zhang et al. 2019b). Experiments using whole communities of freshwater bacteria also showed increased RP4 conjugation at 5–100 µg l^−1^ of zinc, copper, and lead (Wang et al. 2020). Interestingly, cadmium gave somewhat different effects, with high levels (10–100 mg l^−1^) required to stimulate conjugation in freshwater bacteria (Pu et al. 2021). In the IncP-1 plasmids like RP4, metal exposure indirectly decreases expression of the *korA* and *korB* repressor genes, which switches on expression of proconjugation *traC, traF, trbB*, and *trfA* pilus and relaxosome genes. This increased conjugation frequency is likely attributed to reactive oxygen species stimulated DNA repair responses and increased membrane permeability (Zhang et al. 2018b, 2019b, Pu et al. 2021). A key thing to keep in mind here is that if plasmid conjugation frequency is increased by metals, this means that all the other MGEs, ARGs, and MRGs embedded within the plasmid are also transferred at increased frequency.

Promotion of conjugation by metals has also been seen in IncP-1ε resistance plasmids in anaerobic sludge exposed to arsenic (0.1 mM), mercury (5 µM), or lead (0.5 mM) (Lin et al. 2019). These plasmids also carried two-component transcriptional factors, secretion systems and efflux pumps implicated in heavy metal resistance. It is important to note that the stimulation of conjugation by metals is not a universal effect, and decreased conjugation rates were observed for IncP-1ε plasmids in *Pseudomonas, Aeromonas, Escherichia*, and *Enterobacter* in the presence of cadmium, copper, or zinc (Lin et al. 2019). Similar findings were also seen for IncF plasmids in *E. coli* treated with copper, arsenate, or zinc (Palm et al. 2022). Neither of these studies tested whether the transconjugants had enhanced MICs, so it is hard to say whether this represents an example of co-regulation in the sense of metals impacting antibiotic resistance.

## An updated understanding of co-selection

Mechanistic insights on co-selection have progressed in recent decades, but more combinatorial approaches to genetic and phenotypic assessments are required to attain a comprehensive understanding. The distinction of co-resistance requires an appreciation of genomic context, MGEs, and the conditions that facilitate HGT. An assessment of cross-resistance demands characterization of multiple substrates that a resistance gene enacts upon. Co-regulation is even more complex to rigorously identify, requiring detailed molecular studies of gene expression. It is acknowledged that many studies fall short of the level of evidence required to confirm mechanisms of co-selection in microbial communities and the environmental conditions that facilitate this (Pal et al. 2016, Yue et al. 2020). Renewed efforts are needed since a better understanding of co-selection mechanisms will inform predictions and control strategies for antimicrobial resistance.

### Mechanisms driving co-selection

The studies discussed above demonstrate that co-resistance, cross-resistance, and co-regulation are real phenomena, not just in controlled laboratory settings, but *in situ* in the environment also. The evidence for co-resistance to date is greater than for cross-resistance or co-regulation, however, this may be because the evidence for the latter two is more difficult to obtain. The notable MRGs *czcA, copA, pcoA*, and ARGs *aacA4, blaOXA, blaTEM, ermB, ermF, sul1, sul2, tetM*, and *tetW* frequently exhibit correlations with one another. Although individual studies may have certain biases, these MRG–ARG combinations do stand out as probable true cases of co-resistance. Many studies have focused only on a limited number of MRGs and ARGs (Fig. 5). This needs to be expanded with high-throughput methods. Plasmids are frequently found containing both ARG and MRG combinations in metal-contaminated environments, and the HGT of these is enhanced under metal stress. Increasing evidence suggests *intI1* may play a role in co-resistance as well, despite the fact that a specific integron gene cassette conferring metal resistance is yet to be found. Cross-resistance and co-regulation are less reported in the literature, although proof of concept examples have been well-characterized such as MacAB (Shi et al. 2019) and Czc with OprD (Perron et al. 2004).

We do not know how co-selection operates in complex communities, as most studies involve species in isolation (Karkman et al. 2018). Complex communities are more difficult to study, since one species may modify the effect of a metal on another, e.g. oxidation/reduction reactions that change the metal’s toxicity and/or bioavailability. The use of mixed bacterial communities allows HGT to be studied, but only a few studies have explored this (Pal et al. 2017). In some community studies, bacterial community structure is the factor with the greatest impact on ARGs, implying that resistance genes are still taxonomically segregated in some cases, despite manifold correlations between MRGs and ARGs (Yan et al. 2020, Sun et al. 2021, Yang et al. 2021, Huang et al. 2022). This offers an alternative explanation for co-selection, where taxonomic shifts within communities caused by heavy metal exposure promote species that incidentally possess ARGs (Pal et al. 2017). This kind of indirect selection may also underpin positive or negative correlations reported in other studies.

In order to unravel the mechanisms at play, and develop useful predictive models, studies that measure changes to the resistome and mobilome after applying precise selective pressures are needed (Grenni and Corno 2019, Li et al. 2020), and these should aim to obtain multiple lines of evidence at different molecular levels (DNA, RNA, and/or protein). Microcosm experiments offer a practical and effective way to address many of these gaps. Ideally, these experiments need to be long-term (months or years) to effectively document the players involved, their interactions, and the effects of selection. Metagenomic analyses should extend to more than just qPCR as this technique offers little information on genomic context or genetic expression. Genome-level resolution is needed not only to ascribe co-resistance functions but also to determine the likely stability, mobility and expression levels of genes. The movement of genes from plasmids into chromosomes tends to increase their stability (Gullberg et al. 2014), but the reverse process tends to increase the strength of resistance through higher copy number (Shen et al. 2020). Expression measurements (e.g. microarrays, transcriptomics, and RT-qPCR) are especially useful as a counterpart to ‘omics’ studies and can enable insights into whether a putative resistance gene is serving homeostatic or resistance functions. Further confirmation of MRG and ARG expression can be obtained with proteomic techniques such as enzyme activity assays, western blots, or ELISA (Fig. 4). Targeted functional assessments on putative resistance genes can be performed through plasmid capture and characterization, heterologous expression, or gene knock out/down approaches. These approaches are critical for expanding our knowledge of gene functions of novel environmental resistance genes. This is particularly pertinent for efflux pumps, which are often hypothesized to be contributors to cross-resistance, but only rarely have complete substrate range experimentally verified.

To complement the experimental approaches, urgent revision of bioinformatic databases is required. The environmental resistome and mobilome are highly diverse and abundant (Pal et al. 2016), but only a tiny fraction of this diversity is represented in well-curated and well-annotated database entries (Pal et al. 2015, 2017, Perez et al. 2020). The many mismatches between expected genotypes and phenotypes that can be found in the co-selection literature illustrate that our current knowledge of resistance is far from comprehensive. Hits to resistance genes in such databases are further complicated by the fact that many MRGs are part of normal homeostasis machinery and may not be serving a resistance function. Resistance genes that arise from point mutations may be hard to distinguish from wild-type genes. Unfortunately, many resistance gene databases are either no longer curated (ARDB, MEGARes, and BacMet), or they do not distinguish between experimentally verified vs. predicted resistance genes (CARD and ARG-ANNOT) (Bengtsson-Palme et al. 2017). It is likely that there are many undetected or misannotated resistance genes and MGEs in public databases.

### Environmental conditions promoting co-selection

The frequencies and impacts of resistance gene acquisition depend on a complex interplay of factors including the setting (clinical, environmental, urban, and rural) (Pal et al. 2016, Xu et al. 2017), physicochemical factors (Chen et al. 2019a, Zhong et al. 2021), bacterial taxa present (Ma et al. 2019, Huang et al. 2022), types of MGEs present (Pal et al. 2015, Mazhar et al. 2021), and of course, which selective agents are present, and their concentrations (Gao et al. 2015, Xu et al. 2015a)

Although there are strong contenders for heavy metals that have co-selective capacity, further refinement on the concentration and conditions required to achieve is needed. Studies on environmental bacterial communities frequently conclude that exposure to heavy metals has a greater selective effect on ARGs than exposure to antibiotics themselves (Ji et al. 2012, Hubeny et al. 2021, Mazhar et al. 2021). However, this assessment does have the complication that many antibiotics degrade rapidly, so their effect may last far longer than their detectable presence. This can be addressed in comparative simulations, where one microcosm is exposed to only heavy metals and the other to only antibiotics. The co-selective potential of zinc and copper from agricultural or industrial sources has frequently been cited, but there is still no consensus on their relative contributions or importance under real-world conditions. Meanwhile, arsenic, cadmium, and manganese are comparatively understudied but are consistently correlated with ARG dissemination (Knapp et al. 2011, Yan et al. 2020, Zhao et al. 2020) (Fig. 6). The impact of co-selection is concentration dependent (Zhang et al. 2018b), but the MSC or MCC (Arya et al. 2021) for most metals is unknown. Furthermore, the MSC is subject to other factors such as bioavailability (Peltier et al. 2010, Zhong et al. 2021), pH (Sui et al. 2019, Chen et al. 2019a), matrix structure (Zhang et al. 2019a, Hung et al. 2022), and bacterial community composition (Yan et al. 2020, Huang et al. 2022). Most studies do not measure the bioavailable fraction of heavy metals; this can be highly variable depending on the sample type (Olaniran et al. 2013). This makes it hard to compare the relationships between ARGs and heavy metals between studies. Laboratory-based studies typically focus on single selective agent treatments, however, this is not an accurate reflection of real-world conditions, so should be complemented with selective agents combinations to analyse the potential synergistic interactions (Gullberg et al. 2014, Zhao et al. 2018) that can impact bioavailability and MCCs (Song et al. 2017, Arya et al. 2021).

Identifying environmental reservoirs of resistance genes and determining how they are maintained and transmitted is fundamental for improving antimicrobial stewardship. Sediments are now recognized as fertile environments for the co-accumulation of heavy metals, MRGs, and ARGs (Nguyen et al. 2019). In agricultural soils, the addition of animal manure or wastewater sludge adds both antibiotics and metals (Grenni and Corno 2019). This makes these a potential hotspot for co-selection, but it also makes it difficult for us to distinguish the impact of the two separate kinds of agents. To unravel these factors, it would be helpful to have more data from metal-contaminated environments that have minimal antibiotic co-contamination, e.g. industrial and mining wastes. This would help to redress the balance of research, which is currently tipped heavily towards clinical environments, antibiotics, and ARGs.

Knowing which kinds of bacteria harbour specific resistance genes is critical for identifying bacterial agents of concern (Pal et al. 2015, Karkman et al. 2018).The MRG content and MSC/MCC for heavy metals are not known for many bacteria (Gullberg et al. 2014, Song et al. 2017, Arya et al. 2021). Gram-negative bacteria are generalized to be more metal-resistant (Seiler and Berendonk 2012, Nguyen et al. 2019), however, large variations in resistance rates have been observed within individual species (Seiler and Berendonk 2012). The *Enterobacteriaceae* have received considerable focus due to their ease of culture, their role in human disease, and their known carriage of resistance plasmids and class 1 integrons (Li et al. 2017, Nguyen et al. 2019). This over-representation is in some ways warranted, but it has skewed meta-analyses that aim to determine the origins and mechanisms of spread of resistance genes.

Metagenomic investigations can reveal the resistome of both unculturable and culturable bacteria, however, attributing gene to the host can be challenging for metagenomes. *Actinomycetota* and *Pseudomonadota* phyla harbour the largest resistome in environmental communities (Yan et al. 2020, Zhao et al. 2021, Wang et al. 2021b, Huang et al. 2023) (see also Tables 1 and 2), but resistance genes are found throughout the bacterial domain, notably in *Bacteroidota, Bacillota, Planctomycetota, Verrucomicrobiota, Acidobacteriota, Gemmatimonadota*, and *Chloroflexota* (Kothari et al. 2019, Stalder et al. 2019, Zhao et al. 2021, Wang et al. 2021b, Tan et al. 2023). Although many metagenomic analyses do not identify the hosts of resistance genes, it has been estimated that the proportion of plasmids containing both ARGs and MRGs is higher for uncultured bacteria than culturable genera (Pal et al. 2015).

Linking resistance genes to hosts in a nonculture-dependent way can be done with molecular techniques such as epicPCR (emulsion paired-isolation and concatenation PCR), Hi-C, or fluorescent-activated cell sorting (FACS). EpicPCR segregates single cells and then performs single-cell PCR that amplifies both 16S rDNA and target gene and then fuses them together (Spencer et al. 2016). This has been used to taxonomically link ARGs and class 1 integrons in wastewater (Hultman et al. 2018) and sediments (Roman et al. 2021). OilPCR acts in a similar way via single-cell segregation and has enabled the linkage of β-lactamase containing plasmids to specific hosts (Diebold et al. 2021). Hi-C cross-links adjacent DNA, enabling plasmids to be physically bound to chromosomes. This has been applied in environmental communities to link ARGs, plasmids and class 1 integrons to hosts (Stalder et al. 2019). If the MGE and/or host can be genetically manipulated, fluorescent markers may be incorporated and then the movement of the MGE, ARGs, and MRGS can be monitored with FACS. Sequencing of the sorted fluorescent fraction may yield novel recipients that cannot be cultured. Indeed, this technique has discovered ARG-carrying IncP plasmids being transferred to 11 different phyla (Klümper et al. 2015).

Along with knowing which bacterial taxa harbour particular ARGs and MRGs, identifying the MGE vectors for these genes is also important. Our understanding of the types and functions of MGEs in the environment is limited, and databases and bioinformatic methods need to be improved to help organize the vast diversity of MGEs in genomes and metagenomes (Jorgensen et al. 2015, Delaney et al. 2018). It is important to be aware that some biases exist in sequence databases, especially those arising from metagenomic studies. Small, high-copy-number plasmids are probably over-represented because they survive DNA extraction better, are easier to assemble, and are preferentially amplified by multiple displacement amplification (Jorgensen et al. 2015, Kothari et al. 2019). The sequencing technologies can also introduce biases, such as high GC-rich regions impacting sequencing methods (Chen et al. 2013). Our understanding of most plasmid biology arises from the study of plasmids that are compatible with standard lab hosts. This systematic bias warrants more work to develop an accurate picture of the plasmidome of environmental bacterial communities, so this can be better utilized to combat resistance.

## Conclusions

A holistic approach is needed to understand how ARGs and MRGs interact in bacterial communities to enable better management strategies for multidrug-resistant bacteria. The use and disposal of heavy metals must be critically reviewed to mitigate not only their direct toxicity but also their other effects on the acquisition and persistence of antimicrobial resistance genes. There are enough correlational studies to warrant further, more mechanistic investigations into heavy metal and antibiotic co-selection. The intricacies of how different combinations and concentrations of metals, bacterial species, and other factors interact need to be explored. It remains unknown to what extent the models of co-resistance, cross-resistance, or co-regulation occur in bacterial communities, but it seems plausible that all contribute to co-selection. The development of better models of co-selection requires a greater understanding of the MGEs involved and the rates of different HGT processes, especially as these influence the mobilization of resistance markers from environmental organisms to pathogens.

## Supplementary Material

fuae017_Supplemental_File

## References

[bib3] Ali H, Khan E. What are heavy metals? Long-standing controversy over the scientific use of the term ‘heavy metals’—proposal of a comprehensive definition. Toxicol Environ Chem. 2018;100:6–19.

[bib4] Allignet J, Loncle V, Simenel C et al. Sequence of a staphylococcal gene, *vat*, encoding an acetyltransferase inactivating the A-type compounds of virginiamycin-like antibiotics. Gene. 1993;130:91–8. 8344533 10.1016/0378-1119(93)90350-c

[bib5] Arsene-Ploetze F, Chiboub O, Lievremont D et al. Adaptation in toxic environments: comparative genomics of loci carrying antibiotic resistance genes derived from acid mine drainage waters. Environ Sci Pollut Res. 2018;25:1470–83. 10.1007/s11356-017-0535-8 29090447

[bib6] Arya S, Williams A, Reina SV et al. Towards a general model for predicting minimal metal concentrations co-selecting for antibiotic resistance plasmids. Environ Pollut. 2021;275:116602. 33582634 10.1016/j.envpol.2021.116602

[bib7] Babakhani S, Oloomi M. Transposons: the agents of antibiotic resistance in bacteria. J Basic Microbiol. 2018;58:905–17. 30113080 10.1002/jobm.201800204

[bib8] Baharoglu Z, Bikard D, Mazel D. Conjugative DNA transfer induces the bacterial SOS response and promotes antibiotic resistance development through integron activation. PLos Genet. 2010;6:e1001165. 20975940 10.1371/journal.pgen.1001165 PMC2958807

[bib9] Baker-Austin C, Wright MS, Stepanauskas R et al. Co-selection of antibiotic and metal resistance. Trends Microbiol. 2006;14:176–82. 16537105 10.1016/j.tim.2006.02.006

[bib11] Barkay T, Miller SM, Summers AO. Bacterial mercury resistance from atoms to ecosystems. FEMS Microbiol Rev. 2003;27:355–84. 12829275 10.1016/S0168-6445(03)00046-9

[bib12] Bazzi W, Abou Fayad AG, Nasser A et al. Heavy metal toxicity in armed conflicts potentiates AMR in *A. baumannii* by selecting for antibiotic and heavy metal co-resistance mechanisms. Front Microbiol. 2020;11:68. 32117111 10.3389/fmicb.2020.00068 PMC7008767

[bib13] Ben Fekih I, Zhang CK, Li YP et al. Distribution of arsenic resistance genes in prokaryotes. Front Microbiol. 2018;9:2473. 30405552 10.3389/fmicb.2018.02473 PMC6205960

[bib14] Bengtsson-Palme J, Larsson DGJ, Kristiansson E. Using metagenomics to investigate human and environmental resistomes. J Antimicrob Chemother. 2017;72:2690–703. 28673041 10.1093/jac/dkx199

[bib15] Bischofberger AM, Baumgartner M, Pfrunder-Cardozo KR et al. Associations between sensitivity to antibiotics, disinfectants and heavy metals in natural, clinical and laboratory isolates of *Escherichia coli*. Environ Microbiol. 2020;22:2664–79. 32162766 10.1111/1462-2920.14986 PMC7384044

[bib16] Bissonnette L, Champetier S, Buisson JP et al. Characterization of the nonenzymatic chloramphenicol resistance (*cmlA*) gene of the In4 integron of Tn*1696*: similarity of the product to transmembrane transport proteins. J Bacteriol. 1991;173:4493–502. 1648560 10.1128/jb.173.14.4493-4502.1991 PMC208113

[bib17] Blindauer CA, Harrison MD, Parkinson JA et al. A metallothionein containing a zinc finger within a four-metal cluster protects a bacterium from zinc toxicity. Proc Natl Acad Sci USA. 2001;98:9593–8. 11493688 10.1073/pnas.171120098 PMC55497

[bib18] Borremans B, Hobman JL, Provoost A et al. Cloning and functional analysis of the *pbr* lead resistance determinant of *Ralstonia metallidurans* CH34. J Bacteriol. 2001;183:5651–8. 11544228 10.1128/JB.183.19.5651-5658.2001 PMC95457

[bib20] Branco R, Chung AP, Johnston T et al. The chromate-inducible *chrBACF* operon from the transposable element Tn*OtChr* confers resistance to chromium(VI) and superoxide. J Bacteriol. 2008;190:6996–7003. 18776016 10.1128/JB.00289-08 PMC2580707

[bib21] Cai X, Zheng X, Zhang D et al. Microbial characterization of heavy metal resistant bacterial strains isolated from an electroplating wastewater treatment plant. Ecotoxicol Environ Saf. 2019;181:472–80. 31228823 10.1016/j.ecoenv.2019.06.036

[bib22] Cantón R, González-Alba JM, Galán JC. CTX-M enzymes: origin and diffusion. Front Microbiol. 2012;3:110. 22485109 10.3389/fmicb.2012.00110 PMC3316993

[bib23] Carattoli A, Zankari E, García-Fernández A et al. In silico detection and typing of plasmids using PlasmidFinder and plasmid multilocus sequence typing. Antimicrobial Agents Chemother. 2014;58:3895–903. 10.1128/AAC.02412-14 PMC4068535 24777092

[bib25] Chen J, Li J, Zhang H et al. Bacterial heavy-metal and antibiotic resistance genes in a copper tailing dam area in Northern China. Front Microbiol. 2019a;10:1916. 31481945 10.3389/fmicb.2019.01916 PMC6710345

[bib26] Chen QL, Zhu D, An XL et al. Does nano silver promote the selection of antibiotic resistance genes in soil and plant?. Environ Int. 2019b;128:399–406. 31078874 10.1016/j.envint.2019.04.061

[bib27] Chen S, Li X, Sun G et al. Heavy metal induced antibiotic resistance in bacterium LSJC7. Int J Mol Sci. 2015;16:23390–404. 26426011 10.3390/ijms161023390 PMC4632705

[bib28] Chen YC, Liu T, Yu CH et al. Effects of GC bias in next-generation-sequencing data on de novo genome assembly. PLoS One. 2013;8:e62856. 23638157 10.1371/journal.pone.0062856 PMC3639258

[bib31] Chesneau O, Ligeret H, Hosan-Aghaie N et al. Molecular analysis of resistance to streptogramin A compounds conferred by the Vga proteins of staphylococci. Antimicrob Agents Chemother. 2005;49:973–80. 15728891 10.1128/AAC.49.3.973-980.2005 PMC549225

[bib32] Chihomvu P, Stegmann P, Pillay M. Characterization and structure prediction of partial length protein sequences of *pcoA, pcoR* and *chrB* genes from heavy metal resistant bacteria from the Klip River, South Africa. Int J Mol Sci. 2015;16:7352–74. 25837632 10.3390/ijms16047352 PMC4425021

[bib33] Chowdhury N, Suhani S, Purkaystha A et al. Identification of AcrAB-TolC efflux pump genes and detection of mutation in efflux repressor AcrR from omeprazole responsive multidrug-resistant *Escherichia coli* isolates causing urinary tract infections. Microbiol Insights. 2019;12:1178636119889629. 31839709 10.1177/1178636119889629 PMC6893934

[bib34] Christakis CA, Barkay T, Boyd ES. Expanded diversity and phylogeny of *mer* genes broadens mercury resistance paradigms and reveals an origin for MerA among thermophilic archaea. Front Microbiol. 2021;12:682605. 34248899 10.3389/fmicb.2021.682605 PMC8261052

[bib36] Conroy O, Kim EH, McEvoy MM et al. Differing ability to transport nonmetal substrates by two RND-type metal exporters. FEMS Microbiol Lett. 2010;308:115–22. 20497225 10.1111/j.1574-6968.2010.02006.x PMC2917340

[bib37] Delaney S, Murphy R, Walsh F. A comparison of methods for the extraction of plasmids capable of conferring antibiotic resistance in a human pathogen from complex broiler cecal samples. Front Microbiol. 2018;9:12. 30150971 10.3389/fmicb.2018.01731 PMC6100392

[bib39] Depardieu F, Podglajen I, Leclercq R et al. Modes and modulations of antibiotic resistance gene expression. Clin Microbiol Rev. 2007;20:79–144. 17223624 10.1128/CMR.00015-06 PMC1797629

[bib41] Deurenberg RH, Stobberingh EE. The evolution of *Staphylococcus aureus*. Infect Genet Evol. 2008;8:747–63. 18718557 10.1016/j.meegid.2008.07.007

[bib42] Di Cesare A, Eckert EM, Corno G. Co-selection of antibiotic and heavy metal resistance in freshwater bacteria. J Limnol. 2016a;75:59–66.

[bib43] Di Cesare A, Eckert EM, D'Urso S et al. Co-occurrence of integrase 1, antibiotic and heavy metal resistance genes in municipal wastewater treatment plants. Water Res. 2016b;94:208–14. 26945964 10.1016/j.watres.2016.02.049

[bib45] Dickinson AW, Power A, Hansen MG et al. Heavy metal pollution and co-selection for antibiotic resistance: a microbial palaeontology approach. Environ Int. 2019;132:105117. 31473413 10.1016/j.envint.2019.105117

[bib46] Diebold PJ, New FN, Hovan M et al. Linking plasmid-based beta-lactamases to their bacterial hosts using single-cell fusion PCR. Elife. 2021;10:e66834. 34282723 10.7554/eLife.66834 PMC8294855

[bib48] Durso LM, Cook KL. One health and antibiotic resistance in agroecosystems. Ecohealth. 2019;16:414–9. 29541967 10.1007/s10393-018-1324-7 PMC6858902

[bib49] Farias P, Santo CE, Branco R et al. Natural hot spots for gain of multiple resistances: arsenic and antibiotic resistances in heterotrophic, aerobic bacteria from marine hydrothermal vent fields. Appl Environ Microbiol. 2015;81:2534–43. 25636836 10.1128/AEM.03240-14 PMC4357944

[bib50] Feldgarden M, Brover V, Gonzalez-Escalona N et al. AMRFinderPlus and the Reference Gene Catalog facilitate examination of the genomic links among antimicrobial resistance, stress response, and virulence. Sci Rep. 2021;11:12728. 34135355 10.1038/s41598-021-91456-0 PMC8208984

[bib51] Flach CF, Pal C, Svensson CJ et al. Does antifouling paint select for antibiotic resistance?. Sci Total Environ. 2017;590-591:461–8. 28284638 10.1016/j.scitotenv.2017.01.213

[bib52] Florensa AF, Kaas RS, Clausen P et al. ResFinder—an open online resource for identification of antimicrobial resistance genes in next-generation sequencing data and prediction of phenotypes from genotypes. Microb Genomics. 2022;8:000748. 10.1099/mgen.0.000748 PMC8914360 35072601

[bib53] Furlan JPR, Gallo IFL, Stehling EG. Genomic characterization of multidrug-resistant extraintestinal pathogenic *Escherichia coli* isolated from grain culture soils. Pedosphere. 2022;32:495–502.

[bib54] Gao P, He S, Huang S et al. Impacts of coexisting antibiotics, antibacterial residues, and heavy metals on the occurrence of erythromycin resistance genes in urban wastewater. Appl Microbiol Biotechnol. 2015;99:3971–80. 25631280 10.1007/s00253-015-6404-9

[bib55] Ghosh A, Singh A, Ramteke PW et al. Characterization of large plasmids encoding resistance to toxic heavy metals in *Salmonella abortus equi*. Biochem Biophys Res Commun. 2000;272:6–11. 10872795 10.1006/bbrc.2000.2727

[bib57] Gillings M, Boucher Y, Labbate M et al. The evolution of class 1 integrons and the rise of antibiotic resistance. J Bacteriol. 2008;190:5095–100. 18487337 10.1128/JB.00152-08 PMC2447024

[bib58] Gillings MR, Paulsen IT. Microbiology of the Anthropocene. Anthropocene. 2014;5:1–8.

[bib59] Glibota N, Grande MJ, Galvez A et al. Genetic determinants for metal tolerance and antimicrobial resistance detected in bacteria isolated from soils of olive tree farms. Antibiotics. 2020;9:476. 32756388 10.3390/antibiotics9080476 PMC7459592

[bib60] Gorovtsov AV, Sazykin IS, Sazykina MA. The influence of heavy metals, polyaromatic hydrocarbons, and polychlorinated biphenyls pollution on the development of antibiotic resistance in soils. Environ Sci Pollut Res. 2018;25:9283–92. 10.1007/s11356-018-1465-9 29453715

[bib61] Grenni P, Corno G. Knowledge gaps and research needs in bacterial co-resistance in the environment. In: Mandal S, Paul D (eds.), Bacterial Adaptation to Co-Resistance. Singapore: Springer, 2019, 39–59.

[bib62] Guerin E, Cambray G, Sanchez-Alberola N et al. The SOS response controls integron recombination. Science. 2009;324:1034. 19460999 10.1126/science.1172914

[bib63] Gullberg E, Albrecht LM, Karlsson C et al. Selection of a multidrug resistance plasmid by sublethal levels of antibiotics and heavy metals. Mbio. 2014;5:e01918–01914. 25293762 10.1128/mBio.01918-14 PMC4196238

[bib64] Gupta A, Matsui K, Lo JF et al. Molecular basis for resistance to silver cations in *Salmonella*. Nat Med. 1999;5:183–8. 9930866 10.1038/5545

[bib65] Gupta S, Graham DW, Sreekrishnan TR et al. Effects of heavy metals pollution on the co-selection of metal and antibiotic resistance in urban rivers in UK and India. Environ Pollut. 2022;306:119326. 35491000 10.1016/j.envpol.2022.119326

[bib66] Gupta S, Graham DW, Sreekrishnan TR et al. Heavy metal and antibiotic resistance in four Indian and UK rivers with different levels and types of water pollution. Sci Total Environ. 2023;857:159059. 36174689 10.1016/j.scitotenv.2022.159059

[bib67] Hasman H, Aarestrup FM. *tcrB*, a gene conferring transferable copper resistance in *Enterococcus faecium*: occurrence, transferability, and linkage to macrolide and glycopeptide resistance. Antimicrob Agents Chemother. 2002;46:1410–6. 11959576 10.1128/AAC.46.5.1410-1416.2002 PMC127162

[bib68] Hassan MET, van der Lelie D, Springael D et al. Identification of a gene cluster, *czr*, involved in cadmium and zinc resistance in *Pseudomonas aeruginosa*. Gene. 1999;238:417–25. 10570969 10.1016/s0378-1119(99)00349-2

[bib69] Hatfull GF . Bacteriophage genomics. Curr Opin Microbiol. 2008;11:447–53. 18824125 10.1016/j.mib.2008.09.004 PMC2706577

[bib70] Hayashi S, Abe M, Kimoto M et al. The DsbA-DsbB disulfide bond formation system of *Burkholderia cepacia* is involved in the production of protease and alkaline phosphatase, motility, metal resistance, and multi-drug resistance. Microbiol Immunol. 2000;44:41–50. 10711598 10.1111/j.1348-0421.2000.tb01244.x

[bib73] Hooper DC, Jacoby GA. Mechanisms of drug resistance: quinolone resistance. Ann N Y Acad Sci. 2015;1354:12–31. 26190223 10.1111/nyas.12830 PMC4626314

[bib76] Huang F-Y, Zhou S-Y-D, Zhao Y et al. Dissemination of antibiotic resistance genes from landfill leachate to groundwater. J Hazard Mater. 2022;440:129763. 35985216 10.1016/j.jhazmat.2022.129763

[bib77] Huang Q, Huang Y, Li B et al. Metagenomic analysis characterizes resistomes of an acidic, multimetal(loid)-enriched coal source mine drainage treatment system. J Hazard Mater. 2023;448:130898. 36731323 10.1016/j.jhazmat.2023.130898

[bib78] Hubeny J, Harnisz M, Korzeniewska E et al. Industrialization as a source of heavy metals and antibiotics which can enhance the antibiotic resistance in wastewater, sewage sludge and river water. PLoS One. 2021;16:e0252691. 34086804 10.1371/journal.pone.0252691 PMC8177550

[bib79] Hughes VM, Datta N. Conjugative plasmids in bacteria of the pre-antibiotic era. Nature. 1983;302:725–6. 6835408 10.1038/302725a0

[bib80] Hultman J, Tamminen M, Pärnänen K et al. Host range of antibiotic resistance genes in wastewater treatment plant influent and effluent. FEMS Microbiol Ecol. 2018;94:29514229. 10.1093/femsec/fiy038 PMC5939699 29514229

[bib81] Hung WC, Rugh M, Feraud M et al. Influence of soil characteristics and metal(loid)s on antibiotic resistance genes in green stormwater infrastructure in Southern California. J Hazard Mater. 2022;424:127469. 34655877 10.1016/j.jhazmat.2021.127469

[bib85] Ji X, Shen Q, Liu F et al. Antibiotic resistance gene abundances associated with antibiotics and heavy metals in animal manures and agricultural soils adjacent to feedlots in Shanghai; China. J Hazard Mater. 2012;235-236:178–85. 22868748 10.1016/j.jhazmat.2012.07.040

[bib86] Jia J, Guan YJ, Li XJ et al. Phenotype profiles and adaptive preference of *Acinetobacter johnsonii* isolated from Ba River with different environmental backgrounds. Environ Res. 2021;196:10. 10.1016/j.envres.2021.110913 33639142

[bib88] Jorgensen TS, Kiil AS, Hansen MA et al. Current strategies for mobilome research. Front Microbiol. 2015;5:750. 25657641 10.3389/fmicb.2014.00750 PMC4302988

[bib90] Karkman A, Do TT, Walsh F et al. Antibiotic-resistance genes in waste water. Trends Microbiol. 2018;26:220–8. 29033338 10.1016/j.tim.2017.09.005

[bib91] Kaur J, Kumar A, Kaur J. Strategies for optimization of heterologous protein expression in *E. coli*: roadblocks and reinforcements. Int J Biol Macromol. 2018;106:803–22. 28830778 10.1016/j.ijbiomac.2017.08.080

[bib92] King DT, Sobhanifar S, Strynadka NC. One ring to rule them all: current trends in combating bacterial resistance to the β-lactams. Protein Sci. 2016;25:787–803. 26813250 10.1002/pro.2889 PMC4941212

[bib93] Klümper U, Riber L, Dechesne A et al. Broad host range plasmids can invade an unexpectedly diverse fraction of a soil bacterial community. ISME J. 2015;9:934–45. 25333461 10.1038/ismej.2014.191 PMC4817699

[bib95] Knapp CW, Dolfing J, Ehlert PAI et al. Evidence of increasing antibiotic resistance gene abundances in archived soils since 1940. Environ Sci Technol. 2010;44:580–7. 20025282 10.1021/es901221x

[bib96] Knapp CW, McCluskey SM, Singh BK et al. Antibiotic resistance gene abundances correlate with metal and geochemical conditions in archived Scottish soils. PLoS One. 2011;6:6. 10.1371/journal.pone.0027300 PMC3212566 22096547

[bib97] Kothari A, Wu YW, Chandonia JM et al. Large circular plasmids from groundwater plasmidomes span multiple incompatibility groups and are enriched in multimetal resistance genes. Mbio. 2019;10:e02899–02818. 30808697 10.1128/mBio.02899-18 PMC6391923

[bib99] Labbate M, Case RJ, Stokes HW. The integron/gene cassette system: an active player in bacterial adaptation. In: Gogarten MB, Gogarten JP, Olendzenski LC (eds.), Horizontal Gene Transfer: Genomes in Flux. Totowa: Humana Press, 2009, 103–25. 10.1007/978-1-60327-853-9_6 19271181

[bib101] Lakin SM, Dean C, Noyes NR et al. MEGARes: an antimicrobial resistance database for high throughput sequencing. Nucleic Acids Res. 2017;45:D574–d580. 27899569 10.1093/nar/gkw1009 PMC5210519

[bib102] Larsson DGJ, Flach CF. Antibiotic resistance in the environment. Nat Rev Microbiol. 2022;20:257–69. 34737424 10.1038/s41579-021-00649-x PMC8567979

[bib103] Lee LJ, Barrett JA, Poole RK. Genome-wide transcriptional response of chemostat-cultured *Escherichia coli* to zinc. J Bacteriol. 2005;187:1124–34. 15659689 10.1128/JB.187.3.1124-1134.2005 PMC545701

[bib104] Leplae R, Hebrant A, Wodak SJ et al. ACLAME: a classification of mobile genetic elements. Nucleic Acids Res. 2004;32:D45–49. 14681355 10.1093/nar/gkh084 PMC308818

[bib106] Li LG, Xia Y, Zhang T. Co-occurrence of antibiotic and metal resistance genes revealed in complete genome collection. ISME J. 2017;11:651–62. 27959344 10.1038/ismej.2016.155 PMC5322307

[bib107] Li Y, Chen H, Song L et al. Effects on microbiomes and resistomes and the source-specific ecological risks of heavy metals in the sediments of an urban river. J Hazard Mater. 2020;409:124472. 33199139 10.1016/j.jhazmat.2020.124472

[bib108] Li Z, Wang X, Zhang B et al. Transmission mechanisms of antibiotic resistance genes in arsenic-contaminated soil under sulfamethoxazole stress. Environ Pollut. 2023;326:121488. 36958659 10.1016/j.envpol.2023.121488

[bib109] Liebert CA, Hall RM, Summers AO. Transposon Tn*21*, flagship of the floating genome. Microb Mol Biol Rev. 1999;63:507–22. 10.1128/mmbr.63.3.507-522.1999 PMC103744 10477306

[bib110] Lin H, Jiang LT, Li B et al. Screening and evaluation of heavy metals facilitating antibiotic resistance gene transfer in a sludge bacterial community. Sci Total Environ. 2019;695:133862. 31425984 10.1016/j.scitotenv.2019.133862

[bib111] Liu B, Pop M. ARDB–antibiotic resistance genes database. Nucleic Acids Res. 2009;37:D443–447. 18832362 10.1093/nar/gkn656 PMC2686595

[bib112] Liu CC, Yan HC, Sun Y et al. Contribution of enrofloxacin and Cu^2+^ to the antibiotic resistance of bacterial community in a river biofilm. Environ Pollut. 2021;291:118156. 34530240 10.1016/j.envpol.2021.118156

[bib113] Lo Giudice A, Casella P, Bruni V et al. Response of bacterial isolates from Antarctic shallow sediments towards heavy metals, antibiotics and polychlorinated biphenyls. Ecotoxicol. 2013;22:240–50. 10.1007/s10646-012-1020-2 23184332

[bib114] Mab X, Guo N, Ren S et al. Response of antibiotic resistance to the co-existence of chloramphenicol and copper during bio-electrochemical treatment of antibiotic-containing wastewater. Environ Int. 2019;126:127–33. 30797102 10.1016/j.envint.2019.02.002

[bib116] Marrero J, Auling G, Coto O et al. High-level resistance to cobalt and nickel but probably no transenvelope efflux: metal resistance in the Cuban *Serratia marcescens* strain C-1. Microb Ecol. 2007;53:123–33. 17186148 10.1007/s00248-006-9152-7

[bib117] Martins VV, Zanetti MOB, Pitondo-Silva A et al. Aquatic environments polluted with antibiotics and heavy metals: a human health hazard. Environ Sci Pollut Res. 2014;21:5873–8. 10.1007/s11356-014-2509-4 24448880

[bib118] Mašlaňová I, Stříbná S, Doškař J et al. Efficient plasmid transduction to *Staphylococcus aureus* strains insensitive to the lytic action of transducing phage. FEMS Microbiol Lett. 2016;363:27609232. 10.1093/femsle/fnw211 27609232

[bib119] Mata MT, Baquero F, Perez-Diaz JC. A multidrug efflux transporter in *Listeria monocytogenes*. FEMS Microbiol Lett. 2000;187:185–8. 10856655 10.1111/j.1574-6968.2000.tb09158.x

[bib121] Mazhar SH, Li X, Rashid A et al. Co-selection of antibiotic resistance genes, and mobile genetic elements in the presence of heavy metals in poultry farm environments. Sci Total Environ. 2021;755:142702. 33049532 10.1016/j.scitotenv.2020.142702

[bib122] McArthur AG, Waglechner N, Nizam F et al. The comprehensive antibiotic resistance database. Antimicrobial Agents Chemother. 2013;57:3348–57. 10.1128/AAC.00419-13 PMC3697360 23650175

[bib124] Mindlin S, Minakhin L, Petrova M et al. Present-day mercury resistance transposons are common in bacteria preserved in permafrost grounds since the Upper Pleistocene. Res Microbiol. 2005;156:994–1004. 16084067 10.1016/j.resmic.2005.05.011

[bib126] Monchy S, Benotmane MA, Wattiez R et al. Transcriptomic and proteomic analyses of the pMOL30-encoded copper resistance in *Cupriavidus metallidurans* strain CH34. Microbiology. 2006;152:1765–76. 16735739 10.1099/mic.0.28593-0

[bib127] Morar M, Pengelly K, Koteva K et al. Mechanism and diversity of the erythromycin esterase family of enzymes. Biochemistry. 2012;51:1740–51. 22303981 10.1021/bi201790u

[bib128] Moura A, Soares M, Pereira C et al. INTEGRALL: a database and search engine for integrons, integrases and gene cassettes. Bioinformatics. 2009;25:1096–8. 19228805 10.1093/bioinformatics/btp105

[bib130] Murray AK, Zhang L, Snape J et al. Comparing the selective and co-selective effects of different antimicrobials in bacterial communities. Int J Antimicrob Agents. 2019;53:767–73. 30885807 10.1016/j.ijantimicag.2019.03.001 PMC6546120

[bib131] Murray CJL, Ikuta KS, Sharara F et al. Global burden of bacterial antimicrobial resistance in 2019: a systematic analysis. Lancet North Am Ed. 2022;399:629–55. 10.1016/S0140-6736(21)02724-0 PMC8841637 35065702

[bib132] Naik MM, Pandey A, Dubey SK. Biological characterization of lead-enhanced exopolysaccharide produced by a lead resistant *Enterobacter cloacae* strain P2B. Biodegradation. 2012;23:775–83. 22544353 10.1007/s10532-012-9552-y

[bib134] Nguyen CC, Hugie CN, Kile ML et al. Association between heavy metals and antibiotic-resistant human pathogens in environmental reservoirs: a review. Front Environ Sci Eng. 2019;13:46.

[bib136] Nies DH . Microbial heavy-metal resistance. Appl Microbiol Biotechnol. 1999;51:730–50. 10422221 10.1007/s002530051457

[bib135] Nies DH . The cobalt, zinc, and cadmium efflux system CzcABC from *Alcaligenes eutrophus* functions as a cation-proton antibporter in *Eschericia coli*. J Bacteriol. 1995;177:2707–12. 7751279 10.1128/jb.177.10.2707-2712.1995 PMC176940

[bib137] Nishino K, Nikaido E, Yamaguchi A. Regulation of multidrug efflux systems involved in multidrug and metal resistance of *Salmonella enterica* serovar typhimurium. J Bacteriol. 2007;189:9066–75. 17933888 10.1128/JB.01045-07 PMC2168627

[bib138] Nishino K, Yamaguchi A. Analysis of a complete library of putative drug transporter genes in *Escherichia coli*. J Bacteriol. 2001;183:5803–12. 11566977 10.1128/JB.183.20.5803-5812.2001 PMC99656

[bib140] Olaniran AO, Balgobind A, Pillay B. Bioavailability of heavy metals in soil: impact on microbial biodegradation of organic compounds and possible improvement strategies. Int J Mol Sci. 2013;14:10197–228. 23676353 10.3390/ijms140510197 PMC3676836

[bib142] Pal C, Asiani K, Arya S et al. Metal resistance and its association with antibiotic resistance. In: Poole RK (ed.), Microbiology of Metal Ions. Vol. 70. London: Academic Press Ltd-Elsevier Science Ltd, 2017, 261–313. 10.1016/bs.ampbs.2017.02.001 28528649

[bib143] Pal C, Bengtsson-Palme J, Kristiansson E et al. Co-occurrence of resistance genes to antibiotics, biocides and metals reveals novel insights into their co-selection potential. BMC Genomics. 2015;16:14. 26576951 10.1186/s12864-015-2153-5 PMC4650350

[bib144] Pal C, Bengtsson-Palme J, Kristiansson E et al. The structure and diversity of human, animal and environmental resistomes. Microbiome. 2016;4:54. 27717408 10.1186/s40168-016-0199-5 PMC5055678

[bib145] Pal C, Bengtsson-Palme J, Rensing C et al. BacMet: antibacterial biocide and metal resistance genes database. Nucleic Acids Res. 2014;42:D737–743. 24304895 10.1093/nar/gkt1252 PMC3965030

[bib146] Palm M, Fransson A, Hultén J et al. The effect of heavy metals on conjugation efficiency of an F-plasmid in *Escherichia coli*. Antibiotics. 2022;11:1123. 36009992 10.3390/antibiotics11081123 PMC9404890

[bib147] Pansegrau W, Lanka E, Barth PT et al. Complete nucleotide sequence of Birmingham IncP-alpha plasmids: compilation and comparative analysis. J Mol Biol. 1994;239:623–63. 8014987 10.1006/jmbi.1994.1404

[bib148] Pawlowski AC, Stogios PJ, Koteva K et al. The evolution of substrate discrimination in macrolide antibiotic resistance enzymes. Nat Commun. 2018;9:112. 29317655 10.1038/s41467-017-02680-0 PMC5760710

[bib149] Peltier E, Vincent J, Finn C et al. Zinc-induced antibiotic resistance in activated sludge bioreactors. Water Res. 2010;44:3829–36. 20537675 10.1016/j.watres.2010.04.041

[bib151] Perez MF, Kurth D, Farias ME et al. First report on the plasmidome from a high-altitude lake of the Andean Puna. Front Microbiol. 2020;11:15. 32655530 10.3389/fmicb.2020.01343 PMC7324554

[bib152] Perron K, Caille O, Rossier C et al. CzcR-CzcS, a two-component system involved in heavy metal and carbapenem resistance in *Pseudomonas aeruginosa*. J Biol Chem. 2004;279:8761–8. 14679195 10.1074/jbc.M312080200

[bib153] Petrova M, Gorlenko Z, Mindlin S. Tn*5045*, a novel integron-containing antibiotic and chromate resistance transposon isolated from a permafrost bacterium. Res Microbiol. 2011;162:337–45. 21262357 10.1016/j.resmic.2011.01.003

[bib155] Poole K . Efflux-mediated antimicrobial resistance. J Antimicrob Chemother. 2005;56:20–51. 15914491 10.1093/jac/dki171

[bib156] Poulain AJ, Aris-Brosou S, Blais JM et al. Microbial DNA records historical delivery of anthropogenic mercury. ISME J. 2015;9:2541–50. 26057844 10.1038/ismej.2015.86 PMC4817628

[bib157] Pu Q, Fan XT, Li H et al. Cadmium enhances conjugative plasmid transfer to a fresh water microbial community. Environ Pollut. 2021;268:115903. 33120155 10.1016/j.envpol.2020.115903

[bib159] Rensing C, Grass G. *Escherichia coli* mechanisms of copper homeostasis in a changing environment. FEMS Microbiol Rev. 2003;27:197–213. 12829268 10.1016/S0168-6445(03)00049-4

[bib160] Resqu . The resqu database. Gothenburg, 2023.

[bib161] Richmond MH, John M. Co-transduction by a staphylococcal phage of the genes genes responsible for penicillinase synthesis and resistance to mercury salts. Nature. 1964;202:1360–1. 14210993 10.1038/2021360a0

[bib162] Roman VL, Merlin C, Virta MPJ et al. EpicPCR 2.0: technical and methodological improvement of a cutting-edge single-cell genomic approach. Microorganisms. 2021;9:1649. 34442728 10.3390/microorganisms9081649 PMC8399275

[bib163] Rosano GL, Ceccarelli EA. Recombinant protein expression in *Escherichia coli*: advances and challenges. Front Microbiol. 2014;5:172. 24860555 10.3389/fmicb.2014.00172 PMC4029002

[bib164] Rosewarne CP, Pettigrove V, Stokes HW et al. Class 1 integrons in benthic bacterial communities: abundance, association with Tn*402*-like transposition modules and evidence for coselection with heavy-metal resistance. FEMS Microbiol Ecol. 2010;72:35–46. 20132306 10.1111/j.1574-6941.2009.00823.x

[bib165] Rowe CL, Hopkins WA, Congdon JD. Ecotoxicological implications of aquatic disposal of coal combustion residues in the United States: a review. Environ Monit Assess. 2002;80:207–76. 12503897 10.1023/a:1021127120575

[bib168] Schmidt T, Schlegel HG. Combined nickel-cobalt-cadmium resistance encoded by the *ncc* locus of *Alcaligenes xylosoxidans* 31A. J Bacteriol. 1994;176:7045–54. 7961470 10.1128/jb.176.22.7045-7054.1994 PMC197079

[bib169] Schulz-Zunkel C, Krueger F. Trace metal dynamics in floodplain soils of the River Elbe: a review. J Environ Qual. 2009;38:1349–62. 19465710 10.2134/jeq2008.0299

[bib170] Schwarz S, Cardoso M. Nucleotide sequence and phylogeny of a chloramphenicol acetyltransferase encoded by the plasmid pSCS7 from *Staphylococcus aureus*. Antimicrob Agents Chemother. 1991;35:1551–6. 1929326 10.1128/aac.35.8.1551 PMC245217

[bib171] Seiler C, Berendonk TU. Heavy metal driven co-selection of antibiotic resistance in soil and water bodies impacted by agriculture and aquaculture. Front Microbiol. 2012;3:10. 23248620 10.3389/fmicb.2012.00399 PMC3522115

[bib172] Sharma N, Kumari R, Thakur M et al. Molecular dissemination of emerging antibiotic, biocide, and metal co-resistomes in the Himalayan hot springs. J Environ Manage. 2022;307:114569. 35091250 10.1016/j.jenvman.2022.114569

[bib173] Shaw KJ, Rather PN, Hare RS et al. Molecular genetics of aminoglycoside resistance genes and familial relationships of the aminoglycoside-modifying enzymes. Microb Rev. 1993;57:138–63. 10.1128/mr.57.1.138-163.1993 PMC372903 8385262

[bib174] Sheldon PJ, Johnson DA, August PR et al. Characterization of a mitomycin-binding drug resistance mechanism from the producing organism, *Streptomyces lavendulae*. J Bacteriol. 1997;179:1796–804. 9045843 10.1128/jb.179.5.1796-1804.1997 PMC178896

[bib175] Sheldon PJ, Mao YQ, He M et al. Mitomycin resistance in *Streptomyces lavendulae* includes a novel drug-binding-protein-dependent export system. J Bacteriol. 1999;181:2507–12. 10198016 10.1128/jb.181.8.2507-2512.1999 PMC93678

[bib176] Shen Z, Zhang HM, Gao QQ et al. Increased plasmid copy number contributes to the elevated carbapenem resistance in OXA-232-producing *Klebsiella pneumoniae*. Microb Drug Resist. 2020;26:561–8. 31895640 10.1089/mdr.2018.0407

[bib178] Shi K, Cao M, Li C et al. Efflux proteins MacAB confer resistance to arsenite and penicillin/macrolide-type antibiotics in *Agrobacterium tumefaciens* 5A. World J Microbiol Biotechnol. 2019;35:115. 31332542 10.1007/s11274-019-2689-7

[bib179] Siddiqui MT, Mondal AH, Gogry FA et al. Plasmid-mediated ampicillin, quinolone, and heavy metal co-resistance among ESBL-producing isolates from the Yamuna River, New Delhi, India. Antibiotics. 2020;9:826. 33227950 10.3390/antibiotics9110826 PMC7699290

[bib180] Siguier P, Perochon J, Lestrade L et al. ISfinder: the reference centre for bacterial insertion sequences. Nucleic Acids Res. 2006;34:D32–6. 16381877 10.1093/nar/gkj014 PMC1347377

[bib181] Silva I, Tacão M, Henriques I. Selection of antibiotic resistance by metals in a riverine bacterial community. Chemosphere. 2021;263:127936. 33297016 10.1016/j.chemosphere.2020.127936

[bib182] Silveira E, Freitas AR, Antunes P et al. Co-transfer of resistance to high concentrations of copper and first-line antibiotics among *Enterococcus* from different origins (humans, animals, the environment and foods) and clonal lineages. J Antimicrob Chemother. 2014;69:899–906. 24343895 10.1093/jac/dkt479

[bib183] Skandalis N, Maeusli M, Papafotis D et al. Environmental spread of antibiotic resistance. Antibiotics. 2021;10:640. 34071771 10.3390/antibiotics10060640 PMC8226744

[bib184] Sköld O . Resistance to trimethoprim and sulfonamides. Vet Res. 2001;32:261–73. 11432417 10.1051/vetres:2001123

[bib185] Song JX, Rensing C, Holm PE et al. Comparison of metals and tetracycline as selective agents for development of tetracycline resistant bacterial communities in agricultural soil. Environ Sci Technol. 2017;51:3040–7. 28198616 10.1021/acs.est.6b05342

[bib186] Song Y, Yu P, Li B et al. The mosaic accessory gene structures of the SXT/R391-like integrative and conjugative elements derived from *Vibrio* spp. isolated from aquatic products and environment in the Yangtze River Estuary, China. BMC Microbiol. 2013;13:214. 24074349 10.1186/1471-2180-13-214 PMC3850215

[bib187] Spencer SJ, Tamminen MV, Preheim SP et al. Massively parallel sequencing of single cells by epicPCR links functional genes with phylogenetic markers. ISME J. 2016;10:427–36. 26394010 10.1038/ismej.2015.124 PMC4737934

[bib188] Squadrone S . Water environments: metal-tolerant and antibiotic-resistant bacteria. Environ Monit Assess. 2020;192:238. 32173770 10.1007/s10661-020-8191-8

[bib189] Staehlin BM, Gibbons JG, Rokas A et al. Evolution of a heavy metal homeostasis/resistance island reflects increasing copper stress in Enterobacteria. Genome Biol Evol. 2016;8:811–26. 26893455 10.1093/gbe/evw031 PMC4824010

[bib190] Stalder T, Press MO, Sullivan S et al. Linking the resistome and plasmidome to the microbiome. ISME J. 2019;13:2437–46. 31147603 10.1038/s41396-019-0446-4 PMC6776055

[bib191] Strahilevitz J, Jacoby GA, Hooper DC et al. Plasmid-mediated quinolone resistance: a multifaceted threat. Clin Microbiol Rev. 2009;22:664–89. 19822894 10.1128/CMR.00016-09 PMC2772364

[bib192] Sui Q, Zhang J, Chen M et al. Fate of microbial pollutants and evolution of antibiotic resistance in three types of soil amended with swine slurry. Environ Pollut. 2019;245:353–62. 30448505 10.1016/j.envpol.2018.11.003

[bib193] Sun FL, Xu ZT, Fan LL. Response of heavy metal and antibiotic resistance genes and related microorganisms to different heavy metals in activated sludge. J Environ Manage. 2021;300:113754. 34543965 10.1016/j.jenvman.2021.113754

[bib194] Swaine DJ . Trace-elements in coal and their dispersal during combustion. Fuel Process Technol. 1994;39:121–37.

[bib195] Takahashi H, Oshima T, Hobman JL et al. The dynamic balance of import and export of zinc in *Escherichia coli* suggests a heterogeneous population response to stress. J R Soc, Interface. 2015;12:20150069. 25808337 10.1098/rsif.2015.0069 PMC4424684

[bib196] Tan Y, Cao X, Chen S et al. Antibiotic and heavy metal resistance genes in sewage sludge survive during aerobic composting. Sci Total Environ. 2023;866:161386. 36608829 10.1016/j.scitotenv.2023.161386

[bib198] Thomas JC, Oladeinde A, Kieran TJ et al. Co-occurrence of antibiotic, biocide, and heavy metal resistance genes in bacteria from metal and radionuclide contaminated soils at the Savannah River Site. Microb Biotechnol. 2020;13:1179–200. 32363769 10.1111/1751-7915.13578 PMC7264878

[bib200] Timms AR, Steingrimsdottir H, Lehmann AR et al. Mutant sequences in the *rpsL* gene of *Escherichia coli* B/r: mechanistic implications for spontaneous and ultraviolet light mutagenesis. Mol Gen Genetics. 1992;232:89–96. 10.1007/BF00299141 1552908

[bib202] van Hoek AH, Mevius D, Guerra B et al. Acquired antibiotic resistance genes: an overview. Front Microbiol. 2011;2:203. 22046172 10.3389/fmicb.2011.00203 PMC3202223

[bib204] Vats P, Kaur UJ, Rishi P. Heavy metal-induced selection and proliferation of antibiotic resistance: a review. J Appl Microbiol. 2022;132:4058–76. 35170159 10.1111/jam.15492

[bib205] Vignaroli C, Pasquaroli S, Citterio B et al. Antibiotic and heavy metal resistance in enterococci from coastal marine sediment. Environ Pollut. 2018;237:406–13. 29502003 10.1016/j.envpol.2018.02.073

[bib207] Wales AD, Davies RH. Co-selection of resistance to antibiotics, biocides and heavy metals, and its relevance to foodborne pathogens. Antibiotics. 2015;4:567–604. 27025641 10.3390/antibiotics4040567 PMC4790313

[bib208] Wang D, Fierke CA. The BaeSR regulon is involved in defense against zinc toxicity in *E. coli*. Metallomics. 2013;5:372–83. 23446818 10.1039/c3mt20217h PMC3657296

[bib210] Wang Q, Liu L, Hou ZL et al. Heavy metal copper accelerates the conjugative transfer of antibiotic resistance genes in freshwater microcosms. Sci Total Environ. 2020;717:8. 10.1016/j.scitotenv.2020.137055 32065888

[bib212] Wang Q, Xu Y, Liu L et al. The prevalence of ampicillin-resistant opportunistic pathogenic bacteria undergoing selective stress of heavy metal pollutants in the Xiangjiang River, China. Environ Pollut. 2021a;268:115362. 33035873 10.1016/j.envpol.2020.115362

[bib213] Wang X, Lan B, Fei H et al. Heavy metal could drive co-selection of antibiotic resistance in terrestrial subsurface soils. J Hazard Mater. 2021b;411:124848. 33858075 10.1016/j.jhazmat.2020.124848

[bib214] White DG, Goldman JD, Demple B et al. Role of the *acrAB* locus in organic solvent tolerance mediated by expression of *marA, soxS*, or *robA* in *Escherichia coli*. J Bacteriol. 1997;179:6122–6. 9324261 10.1128/jb.179.19.6122-6126.1997 PMC179517

[bib215] Xu Y, Wang X, Tan L et al. Metal impacts on the persistence and proliferation of beta-lactam resistance genes in Xiangjiang River, China. Environ Sci Pollut Res. 2019;26:25208–17. 10.1007/s11356-019-05698-7 31256402

[bib216] Xu Y, Xu J, Mao D et al. Effect of the selective pressure of sub-lethal level of heavy metals on the fate and distribution of ARGs in the catchment scale. Environ Pollut. 2017;220:900–8. 27876226 10.1016/j.envpol.2016.10.074

[bib217] Xu YB, Xu JX, Chen JL et al. Antioxidative responses of *Pseudomonas fluorescens* YZ2 to simultaneous exposure of Zn and Cefradine. Ecotoxicol. 2015a;24:1788–97. 10.1007/s10646-015-1516-7 26141733

[bib218] Xu YB, Zhou Y, Ruan JJ et al. Endogenous nitric oxide in *Pseudomonas fluorescens* ZY2 as mediator against the combined exposure to zinc and cefradine. Ecotoxicol. 2015b;24:835–43. 10.1007/s10646-015-1428-6 25678231

[bib220] Yan C, Wang F, Liu H et al. Deciphering the toxic effects of metals in gold mining area: microbial community tolerance mechanism and change of antibiotic resistance genes. Environ Res. 2020;189:109869. 32678731 10.1016/j.envres.2020.109869

[bib221] Yang F, Zhang FL, Li HP et al. Contribution of environmental factors on the distribution of antibiotic resistance genes in agricultural soil. Eur J Soil Biol. 2021;102:103269.

[bib222] Yu MF, Shu B, Li Z et al. Co-selective pressure of cadmium and doxycycline on the antibiotic and heavy metal resistance genes in ditch wetlands. Front Microbiol. 2022;13:820920. 35250936 10.3389/fmicb.2022.820920 PMC8895241

[bib223] Yu Z, Gunn L, Wall P et al. Antimicrobial resistance and its association with tolerance to heavy metals in agriculture production. Food Microbiol. 2017;64:23–32. 28213031 10.1016/j.fm.2016.12.009

[bib224] Yuan QB, Zhai YF, Mao BY et al. Antibiotic resistance genes and *int*I1 prevalence in a swine wastewater treatment plant and correlation with metal resistance, bacterial community and wastewater parameters. Ecotoxicol Environ Saf. 2018;161:251–9. 29886312 10.1016/j.ecoenv.2018.05.049

[bib225] Yue Z, Zhang J, Zhou Z et al. Pollution characteristics of livestock faeces and the key driver of the spread of antibiotic resistance genes. J Hazard Mater. 2020;409:124957. 33418295 10.1016/j.jhazmat.2020.124957

[bib226] Zhang J, Liu J, Wang Y et al. Profiles and drivers of antibiotic resistance genes distribution in one-stage and two-stage sludge anaerobic digestion based on microwave-H_2_O_2_ pretreatment. Bioresour Technol. 2017;241:573–81. 28601775 10.1016/j.biortech.2017.05.157

[bib227] Zhang J, Lu T, Shen P et al. The role of substrate types and substrate microbial community on the fate of antibiotic resistance genes during anaerobic digestion. Chemosphere. 2019a;229:461–70. 31091487 10.1016/j.chemosphere.2019.05.036

[bib234] Zhang S, Wang Y, Song H et al. Copper nanoparticles and copper ions promote horizontal transfer of plasmid-mediated multi-antibiotic resistance genes across bacterial genera. Environ Int. 2019b;129:478–87. 31158594 10.1016/j.envint.2019.05.054

[bib228] Zhang J, Sui Q, Tong J et al. Sludge bio-drying: effective to reduce both antibiotic resistance genes and mobile genetic elements. Water Res. 2016;106:62–70. 27697685 10.1016/j.watres.2016.09.055

[bib231] Zhang M, Chen L, Ye C et al. Co-selection of antibiotic resistance via copper shock loading on bacteria from a drinking water bio-filter. Environ Pollut. 2018a;233:132–41. 29059628 10.1016/j.envpol.2017.09.084

[bib237] Zhang Y, Gu AZ, Cen T et al. Sub-inhibitory concentrations of heavy metals facilitate the horizontal transfer of plasmid-mediated antibiotic resistance genes in water environment. Environ Pollut. 2018b;237:74–82. 29477117 10.1016/j.envpol.2018.01.032

[bib232] Zhang Q-Q, Ying G-G, Pan C-G et al. Comprehensive evaluation of antibiotics emission and fate in the river basins of China: source analysis, multimedia modeling, and linkage to bacterial resistance. Environ Sci Technol. 2015;49:6772–82. 25961663 10.1021/acs.est.5b00729

[bib238] Zhao WX, Wang B, Yu G. Antibiotic resistance genes in China: occurrence, risk, and correlation among different parameters. Environ Sci Pollut Res. 2018;25:21467–82. 10.1007/s11356-018-2507-z 29948704

[bib239] Zhao X, Shen JP, Zhang LM et al. Arsenic and cadmium as predominant factors shaping the distribution patterns of antibiotic resistance genes in polluted paddy soils. J Hazard Mater. 2020;389:121838. 31848095 10.1016/j.jhazmat.2019.121838

[bib240] Zhao Y, Cocerva T, Cox S et al. Evidence for co-selection of antibiotic resistance genes and mobile genetic elements in metal polluted urban soils. Sci Total Environ. 2019;656:512–20. 30529954 10.1016/j.scitotenv.2018.11.372

[bib242] Zhao YF, Gao JF, Wang ZQ et al. Responses of bacterial communities and resistance genes on microplastics to antibiotics and heavy metals in sewage environment. J Hazard Mater. 2021;402:13. 10.1016/j.jhazmat.2020.123550 33254740

[bib246] Zhong QM, Cruz-Paredes C, Zhang SR et al. Can heavy metal pollution induce bacterial resistance to heavy metals and antibiotics in soils from an ancient land-mine?. J Hazard Mater. 2021;411:124962. 33440279 10.1016/j.jhazmat.2020.124962

[bib247] Zhou Q, Wang M, Zhong X et al. Dissemination of resistance genes in duck/fish polyculture ponds in Guangdong Province: correlations between Cu and Zn and antibiotic resistance genes. Environ Sci Pollut Res Int. 2019;26:8182–93. 30697656 10.1007/s11356-018-04065-2

[bib249] Zhou Y, Niu L, Zhu S et al. Occurrence, abundance, and distribution of sulfonamide and tetracycline resistance genes in agricultural soils across China. Sci Total Environ. 2017;599-600:1977–83. 28558428 10.1016/j.scitotenv.2017.05.152

