# Supplementary material for: Unravelling the mechanisms of antibiotic and heavy metal resistance co-selection in environmental bacteria
Source: FEMS Microbiol Rev. 2024 Jun 19;48(4):fuae017. doi: 10.1093/femsre/fuae017 (PMC11253441; doi:10.1093/femsre/fuae017)
Supplement: fuae017_Supplemental_File [file fuae017_supplemental_file.docx]

# **SUPPLEMENTARY DATA**

**Supplementary Table 1: Summary of previous studies on co-selection of antibiotic and metal microbial resistance.** Published scientific papers in English from years 2010 to current were retrieved with keywords “antibiotic”, and “heavy metal”, or “zinc”, or “Zn”, or “cadmium”, or “Cd”, or “copper” or “Cu”, and “resist*”, and “co-select*”, or “co-resist*”, or “cross-resist*” and “bacteria”. Experimental papers that dealt with clinical or animal microbiomes or manure were excluded. ↑ indicates a significant (p<0.05) positive correlation between abundances, unless diversity or resistance (^R^) is stated. ↓ indicates a significant negative correlation. ↑↑ indicates a significant experimentally verified response. Abx^R^-HM^R^ indicates observed dual phenotype. Relative abundances and standard ion valencies unless stated otherwise.

| Source | Metals | Antibiotics | MRGs/ARGs | Findings- co-selection | Findings- MGEs | Ref. |
| --- | --- | --- | --- | --- | --- | --- |
| Ice core | Co, Cu, Fe, Hg, Ni, Zn | AMP, CHL, ERY, MET, STR, TET, VAN |  | HM^R^ ↑ Abx^R^. |  | (Sherpa *et al.*, 2020) |
| Ice core | Cu, Hg, Ni, Zn | AMP, CHL, KAN, NAL, PEN, STR, TET |  | All isolates Abx^R^-HM^R^ to every combination. | 47% isolates possess plasmids. | (Ball *et al.*, 2014) |
| Freshwater-urban | As^3+^, Cd, Co, Cr^6+^, Cu, Hg, Mo, Ni, Pb, Zn | AMP, CHL, CIP, GEN, PMB, STR |  | CHL^R^ ↑ Cd^R^, Cr^R^, Cu^R^, Hg^R^, Ni^R^, Pb^R^. AMP^R^, PMB^R^, STR^R^ ↑ As^R^, Co^R^. |  | (Ghosh *et al.*, 2021) |
| Freshwater-urban | As, Cd, Cu, Pb |  | *erm*(B), *strA*, *strB* *sul1*, *sul2*, *tet*(G), *tet*(M), *tet*(Q), *vanA*, *vanR* | Cd, Pb ↑ *sul2*. Cu ↑ *strB*. |  | (Rajasekar *et al.*, 2022) |
| Freshwater-urban/ *Acinetobacter johnsonii* | As, Cd, Cr, Cu, Pb, Zn | 27 Abx | Resistome | Zn, Cu^R^ ↑ AMP^R^, CAZ^R^. Zn^R^ ↑ CAZ^R^, GEN^R^, NOR^R^, SLX^R^. AMP, CTX ↑ Cu^R^. NOR, ROX, GEN^R^, IPM^R^, NOR^R^, SLX^R^ ↑ Cd^R^. Pb^R^ ↑ AMP^R^. | No ARGs found in prophages. | (Jia *et al.*, 2021) |
| Freshwater-urban/ *Aeromonas* spp. | Cd, Cr, Cu, Pb, Zn | 21 Abx | 4 MRGs, 23 ARGs | HM ↑ *bla_CTX-M_*, *bla_SHV_*, *mexA*, *mexF*, *sul1*. | HGT of *mexA*, *mexF*. | (Jia *et al.*, 2022) |
| Freshwater-urban/*Pseudomonas putida* | Cd | AMP, KAN, TET |  | Cd increased HGT of ARGs. | Cd increased RP4 plasmid conjugation. | (Pu *et al.*, 2021) |
| Freshwater- aquaculture | As, Cd, Cr, Cu, Pb, Se, Zn | CHL, CHT, CIP, DOX, ENR, ERY, FLO, NOR, OXY, ROX, SLM, SLX, SUD, TET, TMP | *catA1*, *cmlA*, erm(F), *erm*(T), *floR*, *qnrA qnrB*, *qnrS*, *sul1*, *sul2*, *tet*(B), *tet*(G), *tet*(X) | As ↑ ARGs except *erm*(T), *tet*(G). Cu ↑ *catA1, erm*(F), *qnrA*, *qnrS*, *tet*(B), *tet*(X). Cr ↑ *cmlA*, *erm*(T), *sul1*. Se ↑ *floR*, *sul1*, *sul2*, *tet*(G). | As, Cr, Se ↑ *intI1*. | (Wang *et al.*, 2022) |
| Freshwater-industrial | Cu, Fe, Mn, Zn | AMP, AMX, CIP, ERY, GEN, KAN, NAL, NEO, NOR, PMB, TET |  | HM ↑ Abx^R^. |  | (Chandra & Sankhwar, 2011) |
| Freshwater-industrial | Cd, Cr, Cu, Mn, Ni, Pb, Zn |  | *aadA, bla_TEM_, cfr, cmlA, ereA, erm*(A), *fexA, floR, qnrD, qnrS, sul1, sul2, tetB*(P), *tet*(C), *tet*(G), *tet*(H), *tet*(O), *tet*(W) | HM ↑ ARGs. Zn ↑ 12 ARGs. Ni ↑ 10 ARGs. Cr, Mn ↑ 7 ARGs. Cu ↑ 5 ARGs. Pb, Cd ↑ 4 ARGs. | MGEs ↑ ARGs. | (Zou *et al.*, 2021) |
| Freshwater-industrial | Cu, Fe, Pb, Zn | AMP, AMX, CHL, ERY, STR, TET, TMP |  | Majority of isolates Abx^R^-HM^R^. |  | (Bosch *et al.*, 2022) |
| Freshwater-industrial |  |  | Resistome | MRGs ↓ ARGs. Genera found with ARGs and MRGs. | MRGs, ARGs ↑ MGEs. | (Huang *et al.*, 2023) |
| Freshwater-industrial/ *Pseudomonas* sp., *Lysinibacillus* sp., *E. coli* | Cr, Cu | AMP, AMX, CEF, NEO, STR, SXT, TET, TOB, VAN | *chrB*, *pcoA, pcoR* | *Pseudomonas* Cr^R^, Cu^R^, AMX^R^, AMP^R^, CEF^R^, STR^R^, VAN^R^. | *Lysinibacillius* Cu^R^, STR^R^ plasmid linked. | (Chihomvu *et al.*, 2015) |
| Freshwater-industrial/*E. coli* | As, Cd, Cu, Hg, Zn | AMP, CAR, CHL, KAN, RIF, TET | Resistome | Abx^R^ but not HM^R^ abundant near mine. |  | (Arsene-Ploetze *et al.*, 2018) |
| Freshwater-hydrothermal |  |  | Resistome | *Geobacillus* containing *czcD*, *hmrR*, *merR* and β-lactam, fluoroquinolone ARGs. | No plasmids detected. | (Najar *et al.*, 2020) |
| Freshwater-hydrothermal | Ag, As^3+^, Ba, Cd, Co, Cu, Fe^3+^, Hg, Mn, Ni, Pb, Zn | AMP, CHL, ERY, KAN, TET, VAN | Resistome | Cd, Zn, Cu, Fe, Cr, Te, Se MRGs ↑ RIF, VAN, Macrolide ARGs. Co, Ni, As, Sb, Hg MRGs ↑ FOF, TMP, TET, CST, Aminoglycoside ARGs. Cu, W, Mo MRGs ↑ TET, CST, VAN, Aminoglycoside ARGs. As, Sb, Hg MRGs ↑ VAN, RIF ARGs. | MRGs and ARGs plasmid linked. | (Sharma *et al.*, 2022) |
| Freshwater-various | Cd, Cu, Hg, Ni, Zn | AMP, AMX, ATM, CAZ, CTX, CXM, KAN, OXA, TET, VAN |  | Majority of isolates VAN^R^, AMP^R^, AMC^R^ - Ni^R^. |  | (Ciftci Turetken *et al.*, 2019) |
| Freshwater-various | Ag, As, Cu, Pb |  | 54 ARG families | Cu, Pb ↑ ARG diversity. | Cu, Pb ↑ ARG *intI* cassettes. | (Drudge *et al.*, 2012) |
| Freshwater-various | Ag, Cd, Co, Cr, Cu, Fe^3+^, Hg, Mn, Ni, Pb, Sb, Sn, Sr, Zn | 26 Abx |  | Minority of isolates HM^R^-Abx^R^. |  | (Icgen & Yilmaz, 2014) |
| Freshwater-various | As, Cd, Co, Cr, Cu, Hg, Pb, Zn |  | 12 MRGs, 19 ARGs | As, *copA* ↑ *sul3*. Hg ↑ *tet*(A). Zn ↑ *qnrA*, *bla_CMY_*. *pcoA* ↑ *ereB*, *qnrS*. *czrC* ↑ *qnrB*, *qnrS*, *sul3*, *tet*(M), *tet*(X). *merA*, *merP* ↑ *bla_CTX-M_*. | *intI1*, *tnpA* ↑ ARGs. | (Zhang *et al.*, 2021) |
| Freshwater-various/ *P. aeruginosa* | Cd, Cu, Hg, Zn | 21 Abx | *copA*, *copB*, *czcA*, *merA*, *tet*(A) | *Pseudomonas* containing *tet*(A), *copA*, *copB*, *czcA*, *merA*. | MRGs and *tet*(A) co-transferred via conjugation. | (Martins *et al.*, 2014) |
| Freshwater-various/*Enterococcus* spp. | Cu | AMP, CHL, CIP, ERY, GEN, NIT, Q-D, STR, TEC, TET, VAN | *copB*, *cueO*, *tcrB*, *aac(6’)-Ie-aph(2”)-Ia*, *aadE*, *erm*(A), *erm*(B), *erm*(C), *mco*, *pbp5*, *tet*(K), *tet*(L), *tet*(M), *tet*(O), *tet*(S), *vanA*, *vanB*, *vanC1*, *vanC2* | *cueO*, *tcrB* ↑ Abx^R^. | Conjugative transfer of *cueO*, *tcrB*. | (Silveira *et al.*, 2014) |
| Freshwater and sediment- urban | As, Cd, Cu, Hg, Pb, Zn |  | 26 ARGs | HM ↑ ARG in sediment but ↓ in water. |  | (Zhao *et al.*, 2022b) |
| Freshwater and sediment-various | Cd, Cu, Hg, Ni, Zn | CHT, CIP, DOX, ENR, ERY, OFX, OXY, ROX, SCP, SLM, SLX, SMM, SUD, TET, TMP | 9 MRGs, 15 ARGs | HM ↑ ARGs, HM most important factor. Cu, Hg, Zn ↑ *sul3*. Cu, Hg ↑ *tet*(A). Ni ↑ *tet*(M), *tet*(W). Hg ↑ *qepA*. Zn, *czcD* ↑ *qnrA*, *qnrB*. Zn ↑ *qnrD*. Cu ↑ *erm*(B). *copB*, *merR* ↑ *tet*(A), *tet*(W). | Cu, Zn ↑ conjugation. *czcD*, *qnrA, qnrB* on one plasmid, while *copB*, *merR*, *tet*(A), *and tet*(W) on another plasmid. | (Xu *et al.*, 2017) |
| Freshwater and sediment-various | Cd, Cu, Hg, Pb, Zn |  | *bla_AMP-C_*, *bla_CMY-2_*, *bla_DHA_*, *bla_GES_*, *bla_OXA-1,_ bla_OXA-2_*, *bla_OXA-10_, bla_SHV_*, *bla_TEM_*, *bla_VIM_* | HM most important factor on ARG abundance. HM ↑ *bla_AMP-C_*, *bla_SHV_*, *bla_TEM_*. Zn, Cu ↑ *bla* ARGs. Hg ↑ *bla_CMY-2_*, *bla_TEM_*. Cu, Pb, Zn ↑ *bla_OXA-1_*. Cd ↑ *bla_SHV_*. Cu, Ni, Pb, Zn ↑ *bla_AMP-C_*, *bla_DHA_*. | High *intI1* abundance. | (Xu *et al.*, 2019) |
| Freshwater and sediment-various | Cd, Co, Cr, Cu, Ni, Pb, Zn | CHL, CIP, ERY, ESBL, MEM, TET | 11 MRGs, 26 ARGs | Cu, Cr, Ni, Co ↑ ESBL^R^, CIP^R^, TET^R^. Co, Cr, Ni ↑ Mer^R^, CHL^R^, ERY^R^, *qnrS*, *tet*(A), *tet*(M), *tet*(Q), *tet*(W), *ereA*, *mefA*. Co, Ni ↑ *bla_VIM_*, *cmlA*. Cr ↑ *bla_CTX-M_*, *bla_TEM_*, *mphA*. Cu ↑ *qnrS*, *tet*(Q), *tet*(W), *ereA*, *mefA*. Pb ↑ *bla_AIM_*. Cu ↓ *mphA*. Co ↓ *bla_NDM-1_*. Co, Ni ↓ *qnrA*. Zn ↓ *bla_OXA-48_*, *bla_IMP_*. | Co, Cr, Ni, *chrA*, most ARGs ↑ *intI1*. *zntA*, *mefA*, *qnrS*, *tet*(A), *tet*(Q), *tet*(W) ↑ *intI2*. | (Gupta *et al.*, 2023) |
| Freshwater, sediment and soil-aquaculture | Cu, Zn |  | 3 MRGs, 23 ARGs | Cu ↑ 10 ARGs, not MRGs. Zn ↑ 17 ARGs, not MRGs. |  | (Zhou *et al.*, 2019) |
| Freshwater, sediment and soil-industrial | Cd, Co, Cr, Hg, Ni | AMC, AMX, APC, CFX, CHL, CIP, ERY, GEN, MTZ, OFX, PEF, SPX, STR, SXT |  | All isolates HM^R^-Abx^R^. |  | (Oyetibo *et al.*, 2010) |
| Freshwater sediment-industrial | Ba, Co, Cr, Cu, Fe, Ga, Mn, Ni, Pb, Sr, Ta, Ti, Zn | CXM, OXA, TMP |  | Zn ↑ CXM^R^, OXA^R^, TMP^R^. | *intI1* ↑ CXM^R^, Zn^R^. | (Dickinson *et al.*, 2019) |
| Freshwater sediment-industrial, urban | As, Cd, Cu, Ni, Pb, Zn |  | 85 ARGs | No correlation between HMs and ARGs. | *intI1* ↑ ARGs. | (Agramont *et al.*, 2020) |
| Freshwater sediment-various | Cd, Co, Cr, Cu, Ni, Pb, Zn |  | 11 MRGs, 26 ARGs | HM explained 83% resistance gene distribution. Co, Ni ↑ *bla_VIM_*, *cmlA*, *ereA*, *mefA*, *qnrS*, *tet*(A), *tet*(M), *tet*(Q), *tet*(S). Cr ↑ *cmlA*, *ereA*, *mefA*, *qnrS*, *tet*(A), *tet*(M), *tet*(Q), *tet*(W). Cu ↑ *ereA*, *mefA*, *tet*(Q), *tet*(W). Co, Cr, Ni ↓ *tet*(O). Zn ↓ *bla_OXA-48_*. Co ↓ *erm*(B). | Co, Cr, Cu, Ni ↑ *intI1*. | (Gupta *et al.*, 2022) |
| Freshwater sediment-various | Cd, Co, Cr, Cu, Fe, Mn, Ni, Pb, V, Zn |  | Resistome | Cd, Cu, Pb, Zn ↑ ARG abundance and diversity. |  | (Li *et al.*, 2020) |
| Freshwater sediment-various | Cd, Cr, Cu, Hg, Ni, Pb, Zn |  | *aadA11* | *aadA11* linked with chromate MRG. | Cu, Hg, Pb, Zn ↑ *intI1*. Tn*402*-like linked with *aadA11* and chromate MRG. | (Rosewarne *et al.*, 2010) |
| Freshwater sediment-various | Cu, Zn |  | *bla_AMP-C_*, *bla_GES_*, *bla_OXA-2_*, *bla_TEM_* | Zn ↑ *bla_AMP-C_*, *bla_OXA-2_*, *bla_TEM_*. Cu ↑ *bla_GES_*, *bla_OXA-2_*, *bla_TEM_*. | Cu, Zn ↑ conjugation. | (Wang *et al.*, 2021a) |
| Wastewater |  |  | *arsB*, *czcA*, *bla_CTX-M_*, *bla_TEM_*, *erm*(B), *qnrS*, *sul2*, *tet*(A) | *sul2* ↑ *czcA*, *arsB*. | *intI1* ↑ *arsB*, *czcA*, *sul2*. | (Di Cesare *et al.*, 2016) |
| Wastewater | Cd, Cr, Cu, Ni, Pb, Zn | CHL, CIP, ERY, OXY, SLX, SUL, TET | *ereA*, *ereB*, *erm*(A), *erm*(B), *erm*(C), *mefA/mefE*, *msrA/msrB* | Zn, Pb ↑ *ereB*, *erm*(B), *mefA/mefE*. |  | (Gao *et al.*, 2015) |
| Wastewater |  |  | Resistome | MRGs ↑ ARGs. |  | (Murray *et al.*, 2019) |
| Wastewater |  |  | *copA*, *czcA*, *mcr−1*, *mphB*, *qnrD*, *sul1*, *tet*(A) | *mcr-1* ↑ *copA*. *mphB* ↑ *czcA*. *sul1* ↓ *copA*. | *intI1* ↑ *czcA*, *mphB*. | (Yuan *et al.*, 2018) |
| Wastewater/ *Acinetobacter baumanii,* *Enterococcus faecalis, E. coli*, *P. putida* | Cd, Cr, Cu, Hg | AMP, CHL, CTX, FOX, GEN, IPM, KAN, NAL, PIP, TET |  | Cd^R^, Cr^R^, Hg^R^ ↑ all Abx^R^ except CTX in *E. coli.* Cd^R^, Cr^R^, Cu^R^ ↑ AMP^R^, FOX^R^ in *A. baumanii*, *P. putida*. | All HM^R^, AMP^R^, CHL^R^, FOX^R^, TET^R^ plasmid linked. | (Mandal *et al.*, 2020) |
| Wastewater | Cd, Cr, Pb, Zn | AMK, AMP, CEF, DOX, GEN, KAN, NAL |  | Majority of isolates HM^R^- Abx^R^. |  | (Yamina *et al.*, 2012) |
| Wastewater |  |  | Resistome | ARGs ↑ MRGs at 1/3 sites. | MRGs ↑ plasmids. | (Di Cesare *et al.*, 2022) |
| Landfill leachate | Cr, Cu, Ni, Zn |  | *bla_EBC_, bla_FOX_, sul1, sul2, tet*(A), *tet*(B), *tet*(C), *tet*(L), *tet*(M), *tet*(O), *tet*(Q), *tet*(S), *tet*(W), *tet*(X) | Cr, Ni, Zn ↑ *sul2*. Cu ↑ *tet*(C), *sul1*. Ni, Zn ↓ *tet*(W), *bla_FOX_*. | Cr, Ni, Zn ↑ *intI1*. | (Zhang *et al.*, 2016b) |
| Landfill leachate | As, Cd, Co, Cr, Cu, Fe, Mn, Ni, Pb, Zn |  | 283 ARGs | HM explained 2% of ARG distribution. All HMs except Cu, Mn ↑ ARGs. |  | (Huang *et al.*, 2022) |
| Wastewater and sludge | As, Co, Cr, Ni, Pb, Zn | CIP, NOR, OFX, OXY, PEF, SLX, SMX, SUT, TET | *aac(6’)-Ib-cr*, *bla_OXA_*, *bla_SHV_*, *bla_TEM_*, *qepA*, *sul1*, *sul2*, *tet*(A), *tet*(M) | HM ↑ ARGs more than tetracyclines, sulfonamides. Cr, Zn ↑ all ARGs. Ni, Pb ↑ all ARGs but *tet*(A). As ↑ all ARGs except *qepA*. | All HMs except Co, all ARGs ↑ *intI1*, *intI2*. | (Hubeny *et al.*, 2021) |
| Wastewater and sludge |  |  | *arsC*, *czcA*, *erm*(B)*, erm*(F)*, mcr-1, merA*, *pcoA, sul1, sul2, tet*(G), *tet*(M), *tet*(X) | MRGs 4^th^ highest factor on ARGs. *czcA* ↑ *sul1*, *sul2*, *tet*(G) in water. *arsC*, *czcA*, *merA* ↑ ARGs. *pcoA* ↑ *tet*(M) in sludge. | *intI1* ↑ *arsC*, *czcA*, *merA*. | (Zhang *et al.*, 2018b) |
| Wastewater and soil-industrial | Cd, Cr^3+, 6+^, Cu, Hg Ni, Zn | AMP, AMX, CHL, DOX, GEN, KAN, MET, STR, SXT, TET |  | HM^R^ isolates not Abx^R^. |  | (Alam *et al.*, 2011) |
| Wastewater sludge |  |  | 3 MRGs, 21 ARGs | *copA* ↑ *qnrD*, *tet*(E), *tet*(Z). *pcoA* ↑ *aac(6’)-lb-cr*, *bla_TEM_*, *sul1*, *sul2*, *tet*(E), *tet*(G), *tet*(Z). *tcrB* ↑ *tet*(M), *qnrD*. | *intI1* ↑ ARGs. | (Jang *et al.*, 2018) |
| Wastewater sludge | Co, Cr, Cu, Mn, Ni |  | Resistome | Ni, Co MRGs ↑ *blaR1*, *erm*. | MRGs IS linked. | (Cai *et al.*, 2019) |
| Wastewater sludge |  |  | Resistome | *“Candidatus* Competibacter denitrificans” contained *cmx* and *corR*. | ARGs, MRGs plasmid, transposon and integron linked. | (Li *et al.*, 2022) |
| Marine boat hull | Cu, Zn | GEN, TET | Resistome | Zn^R^, Cu^R^ ↑ TET^R^. | HM ↑ *intI*, IS. | (Flach *et al.*, 2017) |
| Marine water | Cd, Cr, Cu, Mn, Pb | AMK, AMP,  CFZ, CHL, CXM, FEP, GEN, IPM, KAN, MEM, NAL, NIT, STR, SXT, TET |  | HM^R^ ↑ AMP^R^, CFZ^R^, NIT^R^, STR^R^. |  | (Matyar, 2012) |
| Marine water and sediment | Cd, Cr, Cu, Hg, Pb, Zn | AMC,  AMK, AMP, AMX, AZM, CFX, CHL, CIP, CST, CXM, ERY, GEN, IPM, NAL, OFX, SLX, STD, SXT, TET, TMP |  | ERY^R^ ↑ Cr^R^, Cu^R^. | Tn*21* ↑ *merA*. *intI1* genes ↓ HM^R^. | (Chenia & Jacobs, 2017) |
| Marine water and sediment/*Pseudomonas* spp. | Cd, Cr, Cu, Fe, Ni, Pb, Zn | AMP, AMX, CHL. CIP, ERY, GEN, MET, PEN, TET, VAN |  | Majority of *Pseudomonas* HM^R^-Abx^R^. |  | (Vignesh *et al.*, 2016) |
| Marine water and sediment-hydrothermal | As^3+,5+^, Cd, Co, Cr, Cu, Sb, U, Zn | AMP, CPC, ERY, GEN, KAN, NAL, PMB, RIF, TET, TMP, VAN |  | All HM^R^ and Abx^R^ combinations observed except Abx^R^-Co^R^, ERY^R^-U^R^, RIF^R^-U^R^, GEN^R^-HM^R^. | β-lactam, fluoroquinolone, MDR ARGs, Cu, Co, Zn, Cd MRGs on one plasmid. | (Farias *et al.*, 2015) |
| Marine sediment | Cd, Cu, Hg, Zn | AMP, CHL, KAN, STR |  | No correlation between HM^R^ and Abx^R^. |  | (Lo Giudice *et al.*, 2013) |
| Estuarine and marine sediment/*Enterococcus* spp. | Cd, Cu, Hg | AMP, CHL, CIP, ERY, Q-D, STR, TET, VAN | *cadA*, *merA*, *merB*, *tcrB*, *ant(6)-I*, *blaZ*, *erm*(A), *erm*(B), *mef*, *qnr*, *tet*(L), *tet*(M), *tet*(O), *vatD* | Cd^R^ ↑ ERY^R^. Cd^R^, Cu^R^ ↑ Q-D^R^. |  | (Vignaroli *et al.*, 2018) |
| Estuarine sediment | Cd, Co, Mn, Zn | AMP, CHL, KAN, NAL, STR, TET | Resistome (ARG only) | Majority of isolates HM^R^-Abx^R^. Strain contained multiple ARGs and MRGs. |  | (Vasconcelos *et al.*, 2022) |
| Estuarine sediment- industrial/ *P. aeruginosa* | As, Cr, Cu, Hg, Ni, Zn | 21 Abx | Resistome | Isolate HM^R^ to all except Zn and Abx^R^ to half. ARGs and *cop*, *mer* identified*.* | MDR efflux pumps and *mer* linked to genomic island. | (Teixeira *et al.*, 2016) |
| Estuarine sediment-aquaculture | Cr, Cu, Hg, Pb, Zn |  | 87 ARGs | HM contamination increases ARG abundance and diversity. Cu, Cr ↑ *aac(6’)-Ib-cr*, *blaIMP-5*, *blaIMP-12*, qnrS. | *intI1* not correlated with ARG abundance. | (Zhao *et al.*, 2017) |
|  |  |  |  |  |  |  |
|  |  |  |  |  |  |  |
| Sediment and soil-agricultural | As, Cd, Cr, Cu, Hg, Pb, Zn | CHL, OXY, SLM, SLX, SUD, TET | *sulA*, *sul1*, *sul2*, *sul3*, *tetB*(P), *tet*(M), *tet*(O), *tet*(W) | HM ↑ ARGs stronger than Abx ↑ ARGs. Cu, Zn ↑ *sulA*, *sul3*. Hg ↑ *sulA*. As ↑ *tetB*(P). |  | (Ji *et al.*, 2012) |
| Soil-high altitude |  |  | Resistome | ARGs/MRGs abundant. | ARGs and MRGs plasmid linked. | (Perez *et al.*, 2020) |
| Soil-subsurface | As, Cr, Cu, Ni, Pb, Zn |  | 283 ARGs | High HM explained 29% ARG abundance and diversity. MRGs ↑ ARGs abundance and diversity. | Integrons and transposase ↑ ARGs abundance and diversity. | (Wang *et al.*, 2021b) |
| Soil-urban | As, Cd, Cr, Cu, Ni, Pb, Se, V, Zn |  | *erm*(F), *sul1*, *sul2*, *tet*(A), *tet*(W) | As, Cu, V ↑ *sul1*, *sul2*, *tet*(W). Pb ↑ *sul1*. Zn ↑ *sul2*, *tet*(W). | As, ARGs ↑ *intI1*. | (Hung *et al.*, 2022) |
| Soil-urban | As, Co, Cu, Hg, Mn, Ni, Pb, Se, U, V, Zn |  | 25 ARGs | Mn, Pb, U ↑ *bla_TEM_*. U ↑ *bla_CTX_*. Pb ↑ *bla_OXA_*. As ↑ *bla_SHV_*. Hg ↑ *tet*(A), *tet*(E), *tet*(G). Co ↑ *tet*(M). Ni, V ↓ *bla_SHV_*. Mn, Ni, Pb, Se, V ↓ *tet*(A), *tet*(E), *tet*(G). Se ↓ *tet*(K), *tet*(L), *tet*(M), *tet*(O), *tet*(S). |  | (Knapp *et al.*, 2017) |
| Soil-urban | As, Cd, Co, Cr, Cu, Hg, Ni, Pb, Zn |  | 285 ARGs | HM ↑ ARGs. 24 HM ↑ Abx combinations. | HM toxicity, ARGs ↑ MGEs. | (Zhao *et al.*, 2019) |
| Soil-industrial | As, Cd, Cr, Cu, Ni, Pb, Zn |  | 13 MRGs, 28 ARGs | MRG ↑ ARGs. Zn, Cu ↑ *tetB*(P), *tet*(W). Cd, Pb, Zn ↑ *sul2*. Cr ↑ *tet*(M), *tetB*(P). As ↑ *tetA*(P). Zn ↓ *tet*(S). As ↓ *ereA*. Pb ↓ *tet*(A), *tet*(K), *tet*(S), *tet*(X), *tet*(Q), *ereA*, *bla_CTX-M_*, *bla_SHV_*, *copA*, *pcoA* ↑ 10 ARGs. *copB* *↑* 2 ARGs*. pbrT* ↑ *sul2*. | Cu ↑ *tnpA*. Pb ↓ *tnpA*. *intI1*, *tnpA* ↑ *copA*, *copB*, *pcoA*, many ARGs. | (Chen *et al.*, 2019) |
| Soil-industrial | Cd, Cr, Cu, Ni, Pb | AMP, CHL, KAN, PEN, STR, TET |  | All isolates HM^R^-Abx^R^. |  | (Kumar *et al.*, 2021) |
| Soil-industrial | Cd, Cu, Ni, Pb, Zn | AMP, KAN, TET |  | Majority of isolates HM^R^-Abx^R^. |  | (Piotrowska-Seget *et al.*, 2012) |
| Soil-industrial | As, Co, Cr, Cu, Ni, Pb, Sr, U, Zn |  | Resistome (ARG only) | Higher ARG abundance at HM contaminated sites. |  | (Thomas *et al.*, 2020) |
| Soil-industrial | Cu, Ni, Pb, Zn | AMP, CHL, KAN, TET |  | Ni^R^, Cu^R^ ↑ AMP^R^, CHL^R^. |  | (Timkova *et al.*, 2020) |
| Soil-industrial | As, Cd, Cr, Cu, Hg, Mn, Ni, Pb, Zn |  | *aacC1*, *bla_OKP_*, *cepA*, *cfxA*, *mphB*, *msrA*, *pbp5*, *penA*, *sulA*, *sul1*, *sul2*, *tet*(A), *tet*(B), *tet*(C), *tet*(M), *tet*(O), *tet*(Q), *tet*(W) | Ni, Mn ↑ *tet*(O). Cu, Zn ↑ *sul1*. Cd, Cr ↓ *sul1*. |  | (Yan *et al.*, 2020) |
| Soil-industrial | As, Cd, Cr, Cu, Hg, Ni, Pb, Zn | CHL, NOR, ROX, SUD, TET | 310 ARGs | HM ↑ ARGs stronger than Abx ↑ ARGs. HMs ↑ 30 ARGs. HMs ↓ 25 ARGs. | Ni ↑ *tnpA-01*. Ni ↓ *tnpA-*03. Cr ↓ *tnpA-03*, *tnpA-06.* MGEs not correlated with ARGs. | (Yang *et al.*, 2021) |
| Soil-industrial | Cu, Pb, Zn | TET, VAN |  | VAN^R^- HM^R^. |  | (Zhong *et al.*, 2021) |
| Soil-industrial | Cd, Pb, Zn | AMP, CHL, KAN, TET | *blaZ*, *mecA* | HM^R^ and Abx^R^ not correlated. |  | (Nosalova *et al.*, 2022) |
| Soil-industrial | Cd, Cu, Fe, Ni, Pb, Zn | AMP, CHL, KAN, TET |  | AMP^R^ ↑ Zn^R^, Cu^R^, Ni^R^. CHL^R^ ↑ Cu^R^, Pb^R^, Fe^R^. KAN^R^ ↑ Zn^R^, Pb^R^, Fe^R^, Cd^R^. TET^R^ ↑ Zn^R^, Cu^R^, Ni^R^, Pb^R^. |  | (Lachka *et al.*, 2023) |
| Soil-industrial /*Bacillus thuringiensis* | Cd, Co, Cr^6+^, Cu, Ni, Pb, Zn | AMP, CHL, ERY, FOF, LIN, NIT, RIF, TET | Resistome | All HM^R^ -all Abx^R^ except CHL, ERY. Genome contains 134 ARGs and 75 MRGs. | 11 ARGs plasmid linked. No MRGs plasmid linked. | (Zuo *et al.*, 2020) |
| Soil-agricultural | As, Cd, Cu, Mn, Ni, Pb, Zn | AMP, DOX, ENR, OFX, OXY, SUD | *aac(6’)-Ib-cr*, *bla_CTX-M_*, *erm*(B), *sul1*, *tet*(M) | Cu ↑ *aac(6’)-Ib-cr*, *bla_CTX-M_*, *tet*(M). |  | (Deng *et al.*, 2020) |
| Soil-agricultural | Cu |  | 17 MRGs, 16 ARGs | *copA* ↑ *tet*(B), *tet*(C), *tet*(D). *copA*, *copC* ↑ *bla_PSE_*_,_ *bla_TEM_*. *zntA* ↑ *tet*(C). |  | (Glibota *et al.*, 2020) |
| Soil-agricultural | Cu |  | 285 ARGs | Cu 2^nd^ most important factor on ARGs. Cu ↑ ARGs. | MGEs ↑ ARGs. | (Hu *et al.*, 2016) |
| Soil-agricultural | Cr, Cu, Ni, Pb, Zn |  | *aadA1*, *tet*(Q), *tet*(W) | Cd, Cr, Cu, Ni, Pb, Zn ↓ *tet*(Q). | Cr, Cu, Ni, Pb, Zn ↑ *korB*. Mn ↓ *korB*. | (Jechalke *et al.*, 2015) |
| Soil-agricultural | As, Cd, Cr, Cu, Ni, Pb, Zn | 49 Abx | 283 ARGs | HM ↑ ARGs stronger than Abx ↑ ARGs. As, Cd, Cu, Pb, Zn ↑ ARGs. Cd ↑ *acrR*, *bl2be_shv2/2bl2_len*, *ceoA*, *ereA*, *tet*(32), *tet*(A), *tet*(R), *vanTE*, *yceL/mdtH*. As ↑ *aacA1*, *bacA*, *erm*(35), *tet*(36), *tet*(H), *tet*(T). Pb ↑ *vanTE*, *pncA*. Cu ↑ *aph3iiia*, *erm*(F), *ceoA*. Zn ↑ *bl2be_shv2/2bl2_len*. Cd ↓ *msrA*. Cu ↓ *bl2be_shv2/2bl2_len*. | Integron/transposon genes ↑ most ARGs. | (Mazhar *et al.*, 2021) |
| Soil-agricultural | Cu, Pb, Zn |  | 38 ARGs | Cu, Pb, Zn ↑ *erm*(B), *erm*(F), *sul1*, *sul2*, *tetB*(P), *tet*(G), *tet*(O), *tet*(W). Cu ↑ *tet*(L), *tet*(Z). | Cu, Pb, Zn, ARGs ↑ *intI1*. | (Peng *et al.*, 2017) |
| Soil-agricultural | Cd, Cr, Cu, Mn, Zn | AMP, CTX, ERY, TET | *bla_TEM_*, *ereA*, *erm*(B), *erm*(F), *tet*(G), *tet*(M), *tet*(X) | HM ↑ *erm*(F), *tet*(G), *tet*(M), *tet*(X). | HM not correlated to *intI1*. | (Sui *et al.*, 2019) |
| Soil-agricultural | Cd, Cu, Pb, Zn |  | 285 ARGs | Pb ↑ ARGs. Zn ↓ ARGs. | MGEs ↑ ARG. | (Wang *et al.*, 2020a) |
| Soil-agricultural | As, Cd, Cr, Cu, Mn, Ni, Pb, Zn |  | *arsC*, *czcA*, *merA*, *pcoA*, *bla_TEM_, ereA, erm*(B)*, erm*(F)*, tet*(G), *tet*(M), *tet*(X) | HM ↑ ARGs. MRG ↑ Abx^R^. | ARGs ↑ *intI1*. | (Zhang *et al.*, 2018a) |
| Soil-agricultural | As, Cd |  | 285 ARGs | As, Cd ↑ ARGs. | MGEs ↑ ARGs. | (Zhao *et al.*, 2020) |
| Soil-agricultural | Cd, Cr, Cu, Pb, Zn |  | *aadA5, erm*(B)*, erm*(F)*, strA*, *sul1, sul2, tet*(O), *tet*(W) | Zn ↑ all ARGs except *tet*. Pb ↑ *erm*(B)*.* | All HMs except Cu ↑ *intI1*, MGEs. | (Zheng *et al.*, 2020) |
| Soil-agricultural | Cr, Cu, Hg, Zn |  | *sul1, sul2, tetB*(P), *tet*(M), *tet*(O), *tet*(Q), *tet*(T), *tet*(W) | Cu, Hg ↑ *sul1*, *sul2*. |  | (Zhou *et al.*, 2017) |
| Soil- agricultural | As, Cd | AMP, AMX, AZM, CHL, DOX, ERY, GEN, LVX, OFX |  | Abx^R^- all HM^R^. |  | (Mokni-Tlili *et al.*, 2022) |
| Soil-agricultural | Cd, Hg, Zn | AMP, CAR, CHL, ERY, TET | *cadA*, *czcA* | HM ↑ Abx^R^. *cadA*, *czcA ↑* Abx^R^. | *cadA*, *czcA* transferred via conjugation. | (Heydari *et al.*, 2022) |
| Soil-agricultural/*E. coli* | Ag^2+^, As, Cd, Co, Cr^2+^, Cu, Fe^3+^, Hg, Mn, Ni, Pb, Se, Te, Zn | 33 Abx | Resistome | Abx^R^- all HM^R^ except Ag^2+^. Many ARGs and MRGs in same genome. |  | (Furlan *et al.*, 2022) |
| Soil-agricultural, marine water | Cd, Cu, Ni, Zn | CHL, RIF |  | Abx^R^-HM^R^. |  | (Mujahid *et al.*, 2014) |
| Soil- industrial and agricultural | Cd |  | Resistome (ARG only) | Cd ↑ ARGs. | MGEs ↑ ARGs. | (Cheng *et al.*, 2021) |
| Soil- industrial and agricultural | Cd, Cu, Fe, Hg, Mn, Ni, Pb, Zn | AMP, AMX, DOX, GEN, STR, TET, VAN |  | Hg^R^ ↑ AMX^R^, AMP^R^, STR^R^, TET^R^, VAN^R^. Ni^R^ ↑ AMX^R^, AMP^R^, VAN^R^. Zn^R^ ↑ AMX^R^, AMP^R^. Cu^R^ ↓ Abx^R^. |  | (Safari Sinegani & Younessi, 2017) |
| Soil-various | Cr, Cu, Fe, Ni, Pb |  | *bla_CTX-M_*, *bla_OXA_*, *bla_SHV_*, *bla_TEM_*, *erm*(B), *erm*(C), *erm*(E), *erm*(F), *tet*(B), *tet*(M), *tet*(Q), *tet*(W) | Cu ↑ *bla_OXA_*, *erm*(B), *erm*(F)*, tet*(M), *tet*(W). Cr ↑ *bla_CTX-M_*, *bla_OXA_*, *tet*(M). Ni ↑ *tet*(W). Fe, Ni, Pb ↑ *tet*(M). Fe, Pb, Zn ↓ *erm*(B). |  | (Knapp *et al.*, 2011) |
| Fly ash | Ag, As, Cd, Co, Cu, Fe, Hg, Mn, Pb, Zn | AMP, CHL, KAN, PEN, RIF, STR, TET |  | Abx^R^-HM^R^. |  | (Roychowdhury *et al.*, 2016) |
| Various environments/ *P. aeruginosa* | Cd, Cu, Hg, Cu, Zn | AMK, ATM, CAZ, CIP, CST, FEP, GEN, IPM, ISE, MEM, MIN, PEF, PIP, SXT, TIM, TIC, TOB, TZP | *copA*, *copB*, *czcA*, *merA* | Abx^R^ not correlated with HM^R^. |  | (Deredjian *et al.*, 2011) |
| Various environments/*E. coli* | Cd, Zn | AMX, CHL, CIP, GEN, PMB, TMP |  | Abx^R^ not correlated with HM^R^. |  | (Bischofberger *et al.*, 2020) |
| Freshwater microcosm | Cu^1+^ | 27 Abx |  | 0.1 mM Cu^1+^ ↑↑ Abx^R^. 0.5 mM Cu^1+^ ↓↓ Abx^R^. |  | (McArthur *et al.*, 2017) |
| Freshwater microcosm | Cu | ENR | Resistome | Cu ↑↑ ENR^R^, fluoroquinolone ARGs. |  | (Liu *et al.*, 2021) |
| Freshwater microcosm | Cu, Zn | CTX, IPM, KAN, TET | *bla_TEM_*, *bla_CTX-M_*, *tet*(A) | Cu ↑↑ CTX^R^, TET^R^. Zn ↑↑ CTX^R^, KAN^R^. | HM not correlated with *intI1*. | (Silva *et al.*, 2021) |
| Freshwater microcosm | Cd, Cu, Pb, Zn | AMP, CHT, CIP, KAN, ROX, SLM, TET |  | Cu ↑↑ Abx^R^. | Cu, Pb, Zn ↑↑ conjugation. | (Wang *et al.*, 2020b) |
| Freshwater microcosm | Cu | CAR, CHL, CIP, CLR, CPC, ERY, KAN, LIN, LVX, RIF, SUD, TET, VAN | 249 ARGs | Cu ↑↑ ARGs, all Abx^R^ except CIP^R^, LVX^R^, TET^R^. | Cu ↑↑ MGEs. | (Zhang *et al.*, 2018c) |
| Sediment microcosm | Cu, Ni, Zn |  | *tet*(B), *tet*(M), *tet*(O), *tet*(R), *tet*(X) | Cu, Ni, Zn ↑ *tetM*. Cu ↑ *tet*(B). | HM not correlated with *intI1*. | (Hu *et al.*, 2022) |
| Wastewater microcosm | Cu | CHL | *cmlA*, *floR*, *sul1*, *tet*(C) | Cu ↑↑ *cmlA*, *sul1*, *tet*(C). Cu ↓↓ *floR*. | Cu ↑↑ *intI1*. | (Ma *et al.*, 2019) |
| Wastewater microcosm | Cd | DOX | *czcA*, *czcB*, *czcC*, *czcD*, *czc*R, *czcS*, *tet*(A), *tet*(B), *tet*(C), *tet*(E), *tet*(G), *tet*(M), *tet*(O), *tet*(Q), *tet*(S), *tet*(W), *tet*(X), *tet*(Y) | DOX ↓↓ MRGs. *czcA*, *czcD ↑* all ARGs except *tet*(Y). | *intI1* ↑ *czcA*, most ARGs. | (Yu *et al.*, 2022) |
| Wastewater microcosm | Cu, Zn | AMP, TET | *chrB*, *copA*, *copB*, *czcA*, *nccA*, *pcoD*, *zntA*, *zraP*, *ampC, bla_CTX-M_, bla_TEM_, fabI, mexE, mexF, tet*(A), *tet*(E), *tet*(M), *tet*(W) | *copA ↑* ARGs*. bla_TEM_* ↑ *copB*, *chrB*, *pcoB*, *zntA. tet*(W) ↑ *chrB*, *pcoD*, *zntA*. *chrB* ↑ *tet*(A), *bla_CTX-M_.* | *tnpA* ↑ ARGs, MRGs. | (Zhao *et al.*, 2021) |
| Wastewater microcosm | Cu | AMP | 8 MRGs, 22 ARGs | Cu ↑↑ *tet*(M), *sul1*, *sul2*, *mexE*. Many ARG ↑ MRG combinations. | *tnpA-04*↑ 7 ARGs, 2 MRGs. IS*613* ↑ 3 ARGs, 2 MRGs. | (Zhao *et al.*, 2022a) |
| Wastewater sludge microcosm | Zn | CIP, OXY, TYL |  | Zn ↑↑ TYL^R^. |  | (Peltier *et al.*, 2010) |
| Wastewater sludge microcosm | As, Cd, Cu, Hg, Pb, Zn |  | *dfrA1*, *sul1*, *tet*(A), *tet*(R) | As, Hg, Pb ↑↑ ARGs. | As, Hg, Pb ↑↑ conjugation. Cd, Cu, Zn ↓↓ conjugation | (Lin *et al.*, 2019) |
| Wastewater sludge microcosm | Cr, Cu |  | Resistome | Cr, Cu, Hg, Ni MRGs ↑ ARGs. Fe, Pb MRGs ↓ ARGs. | HM not correlated with MGEs. | (Sun *et al.*, 2021) |
| Wastewater sludge microcosm | As, Cd, Cr, Cu, Mn, Ni, Pb, Zn |  | *copA*, *pcoR*, *tcrB*, *erm*(B)*, erm*(F)*, sul1, sul2, tet*(A), *tet*(G), *tet*(M), *tet*(Q), *tet*(W), *tet*(X) | Cu ↑ *erm*(F), *sul2*, *tet*(X). As, Cd ↑ *tet*(M), *tet*(Q), *tet*(W). *pcoR*, *tcrB* ↑ *erm*(B), *tet*(M), *tet*(Q), *tet*(W). | MGEs ↑ *pcoR*, *tcrB*. | (Zhang *et al.*, 2016a) |
| Wastewater sludge microcosm |  |  | *copA, czcA*, *pcoA*, *bla_OXA-1_*, *bla_TEM_*, *ereA*, *erm*(B), *erm*(F), *mefA/E*, *sul1*, *sul2*, *tet*(G), *tet*(M), *tet*(X) | MRGs ↑ *bla_TEM_*, *sul1*, *sul2*, *tet*(G). MRGs ↓ *erm*(F). | *ereA* ↑ *intI1*. | (Zhang *et al.*, 2017) |
| Wastewater sludge microcosm |  | 25 Abx | *arsC*, *czcA*, *merA*, *pcoA*, *bla_CTX-M_, bla_TEM_, ereA, erm*(B)*, erm*(F)*, sul1, sul2, mcr-1, mefA, tet*(G), *tet*(M), *tet*(X) | MRGs ↑ ARGs greater than Abx ↑ ARGs. *pcoA*, *czcA* ↑ *bla_TEM_, mcr-1*. *merA ↑ ereA,* *sul1, tet*(G). | *intI1* ↑ *bla_CTX-M_*, *ereA*, *tet*(G), *sul1*. | (Zhang *et al.*, 2019) |
| Wastewater sludge microcosm | Cu |  | *pcoD*, *tcrB*, *erm*(A)*, erm*(B)*, qnrS, tet*(A), *tet*(B), *tet*(O), *tet*(T) | Cu ↑↑ *erm*(A). Cu ↑↑ *erm*(B) ↑ *tcrB*. | Cu ↑↑ *intI1*, *intl2* ↑ *tcrB*. | (Zhou *et al.*, 2021) |
| Wastewater sludge microcosm | As, Cd, Cr, Cu, Hg, Mn, Ni, Pb, Zn |  | 56 MRGs, 186 ARGs | *arsD* ↑ 14 ARGs, *czcD* ↑ 11 ARGs. *copB*, *pcoB* ↑ 5 ARGs. *zntA*, *zntB* ↑ 12 ARGs. *vanB* ↑ 6 MRGs. | 18 MGEs ↑ ARGs and MRGS. *intI1* ↑ 29 ARGs, 5 MRGs. | (Tan *et al.*, 2023) |
| Soil microcosm | Cd, Hg, Zn | AMP, CAR, CHL, ERY, TET |  | HM ↑↑ Abx^R^ ↑ HM^R^. |  | (Heydari *et al.*, 2023) |
| Soil microcosm | Cu |  | 285 ARGs | Cu ↑↑ ARG. | Transposases, integrases ↑ ARGs. | (Kang *et al.*, 2018) |
| Soil microcosm | Cu, Zn | TET |  | Cu, Zn ↑↑ TET^R^. |  | (Song *et al.*, 2017) |
| Soil microcosm | Zn |  | Resistome (ARG only) | Zn ↑↑ ARGs. | Zn ↑↑ integrons, IS. | (Tongyi *et al.*, 2020) |
| Agricultural waste microcosm | Cu |  | *copA*, *cueO*, *cusA*, *pcoA*, *tcrB*, *bla_CTX_*, *bla_VIM_*, *drfA7*, *erm*(F), *erm*(Q), *erm*(X), *qnrA*, *qnrS*, *sul1*, *sul2*, *tet*(C), *tet*(G), *tet*(M), *tet*(X) | Cu ↑↑ ARGs. MRGs ↑ ARG diversity. | Cu ↑↑ MGEs. | (Zhang *et al.*, 2022) |
| Genome database |  |  | Resistome | β-lactam, BAC ARGs ↑ As, Cu, Zn MRGs. KAS ARGs ↑ Cu MRGs. PMB ARGs ↑ Zn MRGs. FOF ARGs ↑ As MRGs. | β-lactam, BAC ARGs, Cu, Hg, Zn MRGs plasmid linked. | (Li *et al.*, 2017) |
| Genome database |  |  | Resistome | MRG and ARG co-occur in 17% of genomes. Cd, Hg, Zn MRGs ↑ ARG. | ARG and MRGs co-occur in 5% of plasmids. Hg MRGs ↑ *intI1*, transposase. | (Pal *et al.*, 2015) |
| Genome database |  |  | Resistome | Industrially contaminated environments ↑ resistome abundance and diversity. ARGs not correlated with MRGs. | External environments greater MGE abundance and diversity than human or animal microbiome. | (Pal *et al.*, 2016) |

HM=heavy metals, Abx= antibiotics, MDR=Multidrug resistant, IS=insertion sequence, AMK=amikacin, AMP=ampicillin, AMX=amoxicillin, AMC=amoxicillin-clavulanic acid, APC= ampicillin-cloxacillin, ATM=aztreonam, AZM=azithromycin, BAC=bacitracin, CAR=carbenicillin, CAZ=ceftazidime, CEF=cephalothin, CFX=ceftriaxome, CFZ= cefazolin, CHL=chloramphenicol, CHT=chlortetracycline, CIP=ciprofloxacin, CLR=clarithromycin, CPC=cephalosporin C, CST=colistin, CTX=cefotaxime, CXM=cefuroxime, DOX=doxycycline, ENR=enrofloxacin, ERY=erythromycin, FEP=cefepime, FLO=florfenicol, FOF=fosfomycin, FOX=cefoxitin, GEN=gentamicin, IPM=imipenem, ISE=isepamicin, KAN=kanamycin, KAS= kasugamycin, LIN=lincomycin,LVX=levofloxacin, MEM=meropenen, MET=methicillin, MIN=minocycline, MTZ=metronidazole, NAL=nalidixic acid, NEO=neomycin, NIT=nitrofurantoin, NOR=norfloxacin, OFX=ofloxacin, OXA=oxacillin, OXY=oxytetracycline, PEF=pefloxacin, PEN=penicillin-G, PIP=piperacillin, PMB=polymyxin B, Q-D=quinopristin-dalfopristin, RIF=rifampicin, ROX=roxithromycin, SCP= sulfachlorpyridazine, SLM=sulfamethazine, SLX=sulfamethoxazole, SMM=sulfamonomethoxine, SMX= sulfadimethoxine, SPX=sparfloxacin, STD=sulfatriad, STR=streptomycin, SUD=sulfadiazine, SUL= sulfonamide, SUT=sulfathiazole, SXT= sulfamethoxazole-trimethoprim, TEC=teicoplanin, TET=tetracycline, TIC=ticarcillin, TIM=ticarcillin-clavulanic acid, TMP=trimethoprim, TOB=tobramycin, TYL=tylosin, TZP- piperacillin-tazobactam, VAN=vancomycin.

**Supplementary references**

Agramont J, Gutierrez-Cortez S, Joffre E*, et al.* Fecal pollution drives antibiotic resistance and class 1 integron abundance in aquatic environments of the Bolivian Andes impacted by mining and wastewater. *Microorganisms* 2020; **8**: 1122.

Alam MZ, Ahmad S, Malik A. Prevalence of heavy metal resistance in bacteria isolated from tannery effluents and affected soil. *Environ Monit Assess* 2011; **178**: 281-291.

Arsene-Ploetze F, Chiboub O, Lievremont D*, et al.* Adaptation in toxic environments: comparative genomics of loci carrying antibiotic resistance genes derived from acid mine drainage waters. *Environ Sci Pollut Res Int* 2018; **25**: 1470-1483.

Ball MM, Gomez W, Magallanes X*, et al.* Bacteria recovered from a high-altitude, tropical glacier in Venezuelan Andes. *World J Microbiol Biotechnol* 2014; **30**: 931-941.

Bischofberger AM, Baumgartner M, Pfrunder-Cardozo KR*, et al.* Associations between sensitivity to antibiotics, disinfectants and heavy metals in natural, clinical and laboratory isolates of *Escherichia coli*. *Environ Microbiol* 2020; **22**: 2664-2679.

Bosch J, Bezuidenhout C, Coertze R*, et al.* Metal- and antibiotic-resistant heterotrophic plate count bacteria from a gold mine impacted river: the Mooi River system, South Africa. *Environ Sci Pollut Res Int* 2022.

Cai X, Zheng X, Zhang D*, et al.* Microbial characterization of heavy metal resistant bacterial strains isolated from an electroplating wastewater treatment plant. *Ecotoxicol Environ Saf* 2019; **181**: 472-480.

Chandra R, Sankhwar M. Influence of lignin, pentachlorophenol and heavy metal on antibiotic resistance of pathogenic bacteria isolated from pulp paper mill effluent contaminated river water. *J Environ Biol* 2011; **32**: 739-745.

Chen J, Li J, Zhang H*, et al.* Bacterial heavy-metal and antibiotic resistance genes in a copper tailing dam area in Northern China. *Front Microbiol* 2019; **10**.

Cheng JH, Tang XY, Guan Z*, et al.* Occurrence of antibiotic resistome in farmland soils near phosphorus chemical industrial area. *Sci Total Environ* 2021; **796**: 149053.

Chenia HY, Jacobs A. Antimicrobial resistance, heavy metal resistance and integron content in bacteria isolated from a South African tilapia aquaculture system. *Dis Aquat Organ* 2017; **126**: 199-209.

Chihomvu P, Stegmann P, Pillay M. Characterization and structure prediction of partial length protein sequences of *pcoA*, *pcoR* and *chrB* genes from heavy metal resistant bacteria from the Klip River, South Africa. *Int J Mol Sci* 2015; **16**: 7352-7374.

Ciftci Turetken PS, Altug G, Cardak M*, et al.* Bacteriological quality, heavy metal and antibiotic resistance in Sapanca Lake, Turkey. *Environ Monit Assess* 2019; **191**: 469.

Deng W, Zhang A, Chen S*, et al.* Heavy metals, antibiotics and nutrients affect the bacterial community and resistance genes in chicken manure composting and fertilized soil. *J Environ Manage* 2020; **257**: 109980.

Deredjian A, Colinon C, Brothier E*, et al.* Antibiotic and metal resistance among hospital and outdoor strains of *Pseudomonas aeruginosa*. *Res Microbiol* 2011; **162**: 689-700.

Di Cesare A, Eckert EM, D'Urso S*, et al.* Co-occurrence of integrase 1, antibiotic and heavy metal resistance genes in municipal wastewater treatment plants. *Water Res* 2016; **94**: 208-214.

Di Cesare A, Sabatino R, Yang Y*, et al.* Contribution of plasmidome, metal resistome and integrases to the persistence of the antibiotic resistome in aquatic environments. *Environ Pollut* 2022; **297**: 118774.

Dickinson AW, Power A, Hansen MG*, et al.* Heavy metal pollution and co-selection for antibiotic resistance: A microbial palaeontology approach. *Environ Int* 2019; **132**: 105117.

Drudge CN, Elliott AV, Plach JM*, et al.* Diversity of integron- and culture-associated antibiotic resistance genes in freshwater floc. *Appl Environ Microbiol* 2012; **78**: 4367-4372.

Farias P, Santo CE, Branco R*, et al.* Natural hot spots for gain of multiple resistances: arsenic and antibiotic resistances in heterotrophic, aerobic bacteria from marine hydrothermal vent fields. *Appl Environ Microbiol* 2015; **81**: 2534-2543.

Flach CF, Pal C, Svensson CJ*, et al.* Does antifouling paint select for antibiotic resistance? *Sci Total Environ* 2017; **590-591**: 461-468.

Furlan JPR, Gallo IFL, Stehling EG. Genomic characterization of multidrug-resistant extraintestinal pathogenic *Escherichia coli* isolated from grain culture soils. *Pedosphere* 2022; **32**: 495-502.

Gao P, He S, Huang S*, et al.* Impacts of coexisting antibiotics, antibacterial residues, and heavy metals on the occurrence of erythromycin resistance genes in urban wastewater. *Appl Microbiol Biotechnol* 2015; **99**: 3971-3980.

Ghosh S, Majumder S, Roychowdhury T. Impact of microbial multi-metal and broad spectrum antibiotic tolerance in urban SW (Adi Ganga, Kolkata) on adjacent groundwater: A future threat. *Groundw Sustain Dev* 2021; **14**: 100608.

Glibota N, Grande MJ, Galvez A*, et al.* Genetic determinants for metal tolerance and antimicrobial resistance detected in bacteria isolated from soils of olive tree farms. *Antibiotics* 2020; **9**: 476.

Gupta S, Graham DW, Sreekrishnan TR*, et al.* Effects of heavy metals pollution on the co-selection of metal and antibiotic resistance in urban rivers in UK and India. *Environ Pollut* 2022; **306**: 119326.

Gupta S, Graham DW, Sreekrishnan TR*, et al.* Heavy metal and antibiotic resistance in four Indian and UK rivers with different levels and types of water pollution. *Sci Total Environ* 2023; **857**: 159059.

Heydari A, Kim ND, Horswell J*, et al.* Co-selection of heavy metal and antibiotic resistance in soil bacteria from agricultural soils in New Zealand. *Sustainability* 2022; **14**: 1790.

Heydari A, Kim ND, Biggs PJ*, et al.* Co-selection of bacterial metal and antibiotic resistance in soil laboratory microcosms. *Antibiotics* 2023; **12**.

Hu HW, Wang JT, Li J*, et al.* Field‐based evidence for copper contamination induced changes of antibiotic resistance in agricultural soils. *Environ Microbiol* 2016; **18**: 3896-3909.

Hu X, Wu C, Shi H*, et al.* Potential threat of antibiotics resistance genes in bioleaching of heavy metals from sediment. *Sci Total Environ* 2022; **814**: 152750.

Huang F-Y, Zhou S-Y-D, Zhao Y*, et al.* Dissemination of antibiotic resistance genes from landfill leachate to groundwater. *J Hazard Mater* 2022; **440**: 129763.

Huang Q, Huang Y, Li B*, et al.* Metagenomic analysis characterizes resistomes of an acidic, multimetal(loid)-enriched coal source mine drainage treatment system. *J Hazard Mater* 2023; **448**: 130898.

Hubeny J, Harnisz M, Korzeniewska E*, et al.* Industrialization as a source of heavy metals and antibiotics which can enhance the antibiotic resistance in wastewater, sewage sludge and river water. *PLoS One* 2021; **16**: e0252691.

Hung WC, Rugh M, Feraud M*, et al.* Influence of soil characteristics and metal(loid)s on antibiotic resistance genes in green stormwater infrastructure in Southern California. *J Hazard Mater* 2022; **424**: 127469.

Icgen B, Yilmaz F. Co-occurrence of antibiotic and heavy metal resistance in Kızılırmak River isolates. *Bull Environ Contam Toxicol* 2014; **93**: 735-743.

Jang HM, Lee J, Choi S*, et al.* Response of antibiotic and heavy metal resistance genes to two different temperature sequences in anaerobic digestion of waste activated sludge. *Bioresour Technol* 2018; **267**: 303-310.

Jechalke S, Broszat M, Lang F*, et al.* Effects of 100 years wastewater irrigation on resistance genes, class 1 integrons and IncP-1 plasmids in Mexican soil. *Front Microbiol* 2015; **6**: 163.

Ji X, Shen Q, Liu F*, et al.* Antibiotic resistance gene abundances associated with antibiotics and heavy metals in animal manures and agricultural soils adjacent to feedlots in Shanghai; China. *J Hazard Mater* 2012; **235-236**: 178-185.

Jia J, Zhu ZL, Xue X*, et al.* Selective pressure governs the composition, antibiotic, and heavy metal resistance profiles of *Aeromonas* spp. isolated from Ba River in Northwest China. *Environ Sci Pollut Res* 2022.

Jia J, Guan YJ, Li XJ*, et al.* Phenotype profiles and adaptive preference of *Acinetobacter johnsonii* isolated from Ba River with different environmental backgrounds. *Environ Res* 2021; **196**: 10.

Kang W, Zhang YJ, Shi X*, et al.* Short-term copper exposure as a selection pressure for antibiotic resistance and metal resistance in an agricultural soil. *Environ Sci Pollut Res Int* 2018; **25**: 29314-29324.

Knapp CW, McCluskey SM, Singh BK*, et al.* Antibiotic resistance gene abundances correlate with metal and geochemical conditions in archived Scottish soils. *PLoS One* 2011; **6**: 6.

Knapp CW, Callan AC, Aitken B*, et al.* Relationship between antibiotic resistance genes and metals in residential soil samples from Western Australia. *Environ Sci Pollut Res Int* 2017; **24**: 2484-2494.

Kumar A, Tripti, Maleva M*, et al.* Synergistic effect of ACC deaminase producing *Pseudomonas* sp. TR15a and siderophore producing *Bacillus aerophilus* TR15c for enhanced growth and copper accumulation in *Helianthus annuus* L. *Chemosphere* 2021; **276**: 130038.

Lachka M, Soltisova K, Nosalova L*, et al.* Metal-containing landfills as a source of antibiotic tolerance. *Environ Monit Assess* 2023; **195**: 262.

Li D, Gao J, Dai H*, et al.* Higher spreading risk of antibacterial biocide and heavy metal resistance genes than antibiotic resistance genes in aerobic granular sludge. *Environ Res* 2022; **212**: 113356.

Li LG, Xia Y, Zhang T. Co-occurrence of antibiotic and metal resistance genes revealed in complete genome collection. *ISME J* 2017; **11**: 651-662.

Li Y, Chen H, Song L*, et al.* Effects on microbiomes and resistomes and the source-specific ecological risks of heavy metals in the sediments of an urban river. *J Hazard Mater* 2020; **409**: 124472.

Lin H, Jiang LT, Li B*, et al.* Screening and evaluation of heavy metals facilitating antibiotic resistance gene transfer in a sludge bacterial community. *Sci Total Environ* 2019; **695**: 133862.

Liu CC, Yan HC, Sun Y*, et al.* Contribution of enrofloxacin and Cu2+ to the antibiotic resistance of bacterial community in a river biofilm. *Environ Pollut* 2021; **291**: 118156.

Lo Giudice A, Casella P, Bruni V*, et al.* Response of bacterial isolates from Antarctic shallow sediments towards heavy metals, antibiotics and polychlorinated biphenyls. *Ecotoxicology* 2013; **22**: 240-250.

Ma X, Guo N, Ren S*, et al.* Response of antibiotic resistance to the co-existence of chloramphenicol and copper during bio-electrochemical treatment of antibiotic-containing wastewater. *Environ Int* 2019; **126**: 127-133.

Mandal M, Das SN, Mandal S. Principal component analysis exploring the association between antibiotic resistance and heavy metal tolerance of plasmid-bearing sewage wastewater bacteria of clinical relevance. *Access Microbiol* 2020; **2**: acmi000095.

Martins VV, Zanetti MOB, Pitondo-Silva A*, et al.* Aquatic environments polluted with antibiotics and heavy metals: A human health hazard. *Environ Sci Pollut Res Int* 2014; **21**: 5873-5878.

Matyar F. Antibiotic and heavy metal resistance in bacteria isolated from the Eastern Mediterranean Sea coast. *Bull Environ Contam Toxicol* 2012; **89**: 551-556.

Mazhar SH, Li X, Rashid A*, et al.* Co-selection of antibiotic resistance genes, and mobile genetic elements in the presence of heavy metals in poultry farm environments. *Sci Total Environ* 2021; **755**: 142702.

McArthur JV, Dicks CA, Bryan AL, Jr.*, et al.* The effects of low-level ionizing radiation and copper exposure on the incidence of antibiotic resistance in lentic biofilm bacteria. *Environ Pollut* 2017; **228**: 390-397.

Mokni-Tlili S, Hechmi S, Ouzari HI*, et al.* Co-occurrence of antibiotic and metal resistance in long-term sewage sludge-amended soils: influence of application rates and pedo-climatic conditions. *Environ Sci Pollut Res Int* 2022; 1-17.

Mujahid TY, Siddiqui K, Ahmed R*, et al.* Isolation and partial characterization of phosphate solubilizing bacteria isolated from soil and marine samples. *Pak J Pharm Sci* 2014; **27**: 1483-1490.

Murray AK, Zhang L, Snape J*, et al.* Comparing the selective and co-selective effects of different antimicrobials in bacterial communities. *Int J Antimicrob Agents* 2019; **53**: 767-773.

Najar IN, Sherpa MT, Das S*, et al.* Diversity analysis and metagenomic insights into antibiotic and metal resistance among Himalayan hot spring bacteriobiome insinuating inherent environmental baseline levels of antibiotic and metal tolerance. *J Glob Antimicrob Resist* 2020; **21**: 342-352.

Nosalova L, Willner J, Fornalczyk A*, et al.* Diversity, heavy metals, and antibiotic resistance in culturable heterotrophic bacteria isolated from former lead–silver–zinc mine heap in Tarnowskie Gory (Silesia, Poland). *Arch Microbiol* 2022; **205**: 26.

Oyetibo GO, Ilori MO, Adebusoye SA*, et al.* Bacteria with dual resistance to elevated concentrations of heavy metals and antibiotics in Nigerian contaminated systems. *Environ Monit Assess* 2010; **168**: 305-314.

Pal C, Bengtsson-Palme J, Kristiansson E*, et al.* Co-occurrence of resistance genes to antibiotics, biocides and metals reveals novel insights into their co-selection potential. *BMC Genomics* 2015; **16**: 14.

Pal C, Bengtsson-Palme J, Kristiansson E*, et al.* The structure and diversity of human, animal and environmental resistomes. *Microbiome* 2016; **4**: 54.

Peltier E, Vincent J, Finn C*, et al.* Zinc-induced antibiotic resistance in activated sludge bioreactors. *Water Res* 2010; **44**: 3829-3836.

Peng S, Feng Y, Wang Y*, et al.* Prevalence of antibiotic resistance genes in soils after continually applied with different manure for 30 years. *J Hazard Mater* 2017; **340**: 16-25.

Perez MF, Kurth D, Farias ME*, et al.* First report on the plasmidome from a high-altitude lake of the Andean Puna. *Front Microbiol* 2020; **11**: 15.

Piotrowska-Seget Z, Besciak G, Bernas T*, et al.* GFP-tagged multimetal-tolerant bacteria and their detection in the rhizosphere of white mustard. *Ann Microbiol* 2012; **62**: 559-567.

Pu Q, Fan XT, Li H*, et al.* Cadmium enhances conjugative plasmid transfer to a fresh water microbial community. *Environ Pollut* 2021; **268**: 115903.

Rajasekar A, Qiu M, Wang B*, et al.* Relationship between water quality, heavy metals and antibiotic resistance genes among three freshwater lakes. *Environ Monit Assess* 2022; **194**: 64.

Rosewarne CP, Pettigrove V, Stokes HW*, et al.* Class 1 integrons in benthic bacterial communities: abundance, association with Tn*402*-like transposition modules and evidence for coselection with heavy-metal resistance. *FEMS Microbiol Ecol* 2010; **72**: 35-46.

Roychowdhury R, Mukherjee P, Roy M. Identification of chromium resistant bacteria from dry fly ash sample of Mejia MTPS Thermal Power Plant, West Bengal, India. *Bull Environ Contam Toxicol* 2016; **96**: 210-216.

Safari Sinegani AA, Younessi N. Antibiotic resistance of bacteria isolated from heavy metal-polluted soils with different land uses. *J Glob Antimicrob Resist* 2017; **10**: 247-255.

Sharma N, Kumari R, Thakur M*, et al.* Molecular dissemination of emerging antibiotic, biocide, and metal co-resistomes in the Himalayan hot springs. *J Environ Manage* 2022; **307**: 114569.

Sherpa MT, Najar IN, Das S*, et al.* Distribution of antibiotic and metal resistance genes in two glaciers of North Sikkim, India. *Ecotoxicol Environ Saf* 2020; **203**: 111037.

Silva I, Tacão M, Henriques I. Selection of antibiotic resistance by metals in a riverine bacterial community. *Chemosphere* 2021; **263**: 127936.

Silveira E, Freitas AR, Antunes P*, et al.* Co-transfer of resistance to high concentrations of copper and first-line antibiotics among *Enterococcus* from different origins (humans, animals, the environment and foods) and clonal lineages. *J Antimicrob Chemother* 2014; **69**: 899-906.

Song JX, Rensing C, Holm PE*, et al.* Comparison of metals and tetracycline as selective agents for development of tetracycline resistant bacterial communities in agricultural soil. *Environ Sci Technol* 2017; **51**: 3040-3047.

Sui Q, Zhang J, Chen M*, et al.* Fate of microbial pollutants and evolution of antibiotic resistance in three types of soil amended with swine slurry. *Environ Pollut* 2019; **245**: 353-362.

Sun FL, Xu ZT, Fan LL. Response of heavy metal and antibiotic resistance genes and related microorganisms to different heavy metals in activated sludge. *J Environ Manage* 2021; **300**: 113754.

Tan Y, Cao X, Chen S*, et al.* Antibiotic and heavy metal resistance genes in sewage sludge survive during aerobic composting. *Sci Total Environ* 2023; **866**: 161386.

Teixeira P, Tacão M, Alves A*, et al.* Antibiotic and metal resistance in a ST395 *Pseudomonas aeruginosa* environmental isolate: A genomics approach. *Mar Pollut Bull* 2016; **110**: 75-81.

Thomas JC, Oladeinde A, Kieran TJ*, et al.* Co-occurrence of antibiotic, biocide, and heavy metal resistance genes in bacteria from metal and radionuclide contaminated soils at the Savannah River Site. *Microb Biotechnol* 2020; **13**: 1179-1200.

Timkova I, Lachka M, Kiskova J*, et al.* High frequency of antibiotic tolerance in deep subsurface heterotrophic cultivable bacteria from the Rozalia Gold Mine, Slovakia. *Environ Sci Pollut Res Int* 2020; **27**: 44036-44044.

Tongyi Y, Yanpeng L, Xingang W*, et al.* Co-selection for antibiotic resistance genes is induced in a soil amended with zinc. *Soil Use Manage* 2020; **36**: 328-337.

Vasconcelos ALS, Andreote FD, Defalco T*, et al.* *Mucilaginibacter sp.* strain metal(loid) and antibiotic resistance isolated from estuarine soil contaminated mine tailing from the Fundão Dam. *Genes* 2022; **13**: 174.

Vignaroli C, Pasquaroli S, Citterio B*, et al.* Antibiotic and heavy metal resistance in enterococci from coastal marine sediment. *Environ Pollut* 2018; **237**: 406-413.

Vignesh S, Dahms HU, Muthukumar K*, et al.* Biomonitoring along the tropical southern Indian coast with multiple biomarkers. *PLoS One* 2016; **11**: e0154105.

Wang L, Wang J, Wang J*, et al.* Soil types influence the characteristic of antibiotic resistance genes in greenhouse soil with long-term manure application. *J Hazard Mater* 2020a; **392**: 122334.

Wang Q, Liu L, Hou ZL*, et al.* Heavy metal copper accelerates the conjugative transfer of antibiotic resistance genes in freshwater microcosms. *Sci Total Environ* 2020b; **717**: 8.

Wang Q, Xu Y, Liu L*, et al.* The prevalence of ampicillin-resistant opportunistic pathogenic bacteria undergoing selective stress of heavy metal pollutants in the Xiangjiang River, China. *Environ Pollut* 2021a; **268**: 115362.

Wang Q, Mao C, Lei L*, et al.* Antibiotic resistance genes and their links with bacteria and environmental factors in three predominant freshwater aquaculture modes. *Ecotoxicol Environ Saf* 2022; **241**: 113832.

Wang X, Lan B, Fei H*, et al.* Heavy metal could drive co-selection of antibiotic resistance in terrestrial subsurface soils. *J Hazard Mater* 2021b; **411**: 124848.

Xu Y, Xu J, Mao D*, et al.* Effect of the selective pressure of sub-lethal level of heavy metals on the fate and distribution of ARGs in the catchment scale. *Environ Pollut* 2017; **220**: 900-908.

Xu Y, Wang X, Tan L*, et al.* Metal impacts on the persistence and proliferation of beta-lactam resistance genes in Xiangjiang River, China. *Environ Sci Pollut Res Int* 2019; **26**: 25208-25217.

Yamina B, Tahar B, Marie Laure F. Isolation and screening of heavy metal resistant bacteria from wastewater: a study of heavy metal co-resistance and antibiotics resistance. *Water Sci Technol* 2012; **66**: 2041-2048.

Yan C, Wang F, Liu H*, et al.* Deciphering the toxic effects of metals in gold mining area: Microbial community tolerance mechanism and change of antibiotic resistance genes. *Environ Res* 2020; **189**: 109869.

Yang F, Zhang FL, Li HP*, et al.* Contribution of environmental factors on the distribution of antibiotic resistance genes in agricultural soil. *Eur J Soil Biol* 2021; **102**: 103269.

Yu MF, Shu B, Li Z*, et al.* Co-selective pressure of cadmium and doxycycline on the antibiotic and heavy metal resistance genes in ditch wetlands. *Front Microbiol* 2022; **13**: 820920.

Yuan QB, Zhai YF, Mao BY*, et al.* Antibiotic resistance genes and *int*I1 prevalence in a swine wastewater treatment plant and correlation with metal resistance, bacterial community and wastewater parameters. *Ecotoxicol Environ Saf* 2018; **161**: 251-259.

Zhang J, Sui Q, Tong J*, et al.* Sludge bio-drying: Effective to reduce both antibiotic resistance genes and mobile genetic elements. *Water Res* 2016a; **106**: 62-70.

Zhang J, Sui Q, Tong J*, et al.* Soil types influence the fate of antibiotic-resistant bacteria and antibiotic resistance genes following the land application of sludge composts. *Environ Int* 2018a; **118**: 34-43.

Zhang J, Yang M, Zhong H*, et al.* Deciphering the factors influencing the discrepant fate of antibiotic resistance genes in sludge and water phases during municipal wastewater treatment. *Bioresour Technol* 2018b; **265**: 310-319.

Zhang J, Lu T, Shen P*, et al.* The role of substrate types and substrate microbial community on the fate of antibiotic resistance genes during anaerobic digestion. *Chemosphere* 2019; **229**: 461-470.

Zhang J, Liu J, Wang Y*, et al.* Profiles and drivers of antibiotic resistance genes distribution in one-stage and two-stage sludge anaerobic digestion based on microwave-H_2_O_2_ pretreatment. *Bioresour Technol* 2017; **241**: 573-581.

Zhang M, Chen L, Ye C*, et al.* Co-selection of antibiotic resistance via copper shock loading on bacteria from a drinking water bio-filter. *Environ Pollut* 2018c; **233**: 132-141.

Zhang R, Li J, Zhou L*, et al.* Effect of graphene and graphene oxide on antibiotic resistance genes during copper-contained swine manure anaerobic digestion. *Environ Sci Pollut Res Int* 2022.

Zhang W, Suyamud B, Lohwacharin J*, et al.* Large-scale pattern of resistance genes and bacterial community in the tap water along the middle and low reaches of the Yangtze River. *Ecotoxicol Environ Saf* 2021; **208**: 111517.

Zhang XH, Xu YB, He XL*, et al.* Occurrence of antibiotic resistance genes in landfill leachate treatment plant and its effluent-receiving soil and surface water. *Environ Pollut* 2016b; **218**: 1255-1261.

Zhao X, Shen JP, Zhang LM*, et al.* Arsenic and cadmium as predominant factors shaping the distribution patterns of antibiotic resistance genes in polluted paddy soils. *J Hazard Mater* 2020; **389**: 121838.

Zhao Y, Cocerva T, Cox S*, et al.* Evidence for co-selection of antibiotic resistance genes and mobile genetic elements in metal polluted urban soils. *Sci Total Environ* 2019; **656**: 512-520.

Zhao Y, Gao J, Wang Z*, et al.* Distinct bacterial communities and resistance genes enriched by triclocarban-contaminated polyethylene microplastics in antibiotics and heavy metals polluted sewage environment. *Sci Total Environ* 2022a; **839**: 156330.

Zhao YF, Gao JF, Wang ZQ*, et al.* Responses of bacterial communities and resistance genes on microplastics to antibiotics and heavy metals in sewage environment. *J Hazard Mater* 2021; **402**: 13.

Zhao Z, Wang J, Han Y*, et al.* Nutrients, heavy metals and microbial communities co-driven distribution of antibiotic resistance genes in adjacent environment of mariculture. *Environ Pollut* 2017; **220**: 909-918.

Zhao Z, Li C, Jiang L*, et al.* Occurrence and distribution of antibiotic resistant bacteria and genes in the Fuhe urban river and its driving mechanism. *Sci Total Environ* 2022b; **825**: 153950.

Zheng H, Wang R, Zhang Q*, et al.* Pyroligneous acid mitigated dissemination of antibiotic resistance genes in soil. *Environ Int* 2020; **145**: 106158.

Zhong QM, Cruz-Paredes C, Zhang SR*, et al.* Can heavy metal pollution induce bacterial resistance to heavy metals and antibiotics in soils from an ancient land-mine? *J Hazard Mater* 2021; **411**: 124962.

Zhou Q, Zhou T, Feng F*, et al.* The response of copper resistance genes, antibiotic resistance genes, and intl1/2 to copper addition during anaerobic digestion in laboratory. *Ecotoxicol Environ Saf* 2021; **210**: 111822.

Zhou Q, Wang M, Zhong X*, et al.* Dissemination of resistance genes in duck/fish polyculture ponds in Guangdong Province: correlations between Cu and Zn and antibiotic resistance genes. *Environ Sci Pollut Res Int* 2019; **26**: 8182-8193.

Zhou Y, Niu L, Zhu S*, et al.* Occurrence, abundance, and distribution of sulfonamide and tetracycline resistance genes in agricultural soils across China. *Sci Total Environ* 2017; **599-600**: 1977-1983.

Zou HY, He LY, Gao FZ*, et al.* Antibiotic resistance genes in surface water and groundwater from mining affected environments. *Sci Total Environ* 2021; **772**: 145516.

Zuo W, Li J, Zheng J*, et al.* Whole genome sequencing of a multidrug-resistant *Bacillus thuringiensis* HM-311 obtained from the radiation and heavy metal-polluted soil. *J Glob Antimicrob Resist* 2020; **21**: 275-277.
